# Local convergence of the boundary element method on polyhedral domains

**DOI:** 10.1007/s00211-018-0975-1

**Published:** 2018-06-29

**Authors:** Markus Faustmann, Jens Markus Melenk

**Affiliations:** Institute of Analysis and Scientific Computing (Inst. E 101), TU Wien, Wiedner Hauptstraße 8-10, 1040 Vienna, Austria

**Keywords:** 65N38

## Abstract

The local behavior of the lowest order boundary element method on quasi-uniform meshes for Symm’s integral equation and the stabilized hyper-singular integral equation on polygonal/polyhedral Lipschitz domains is analyzed. We prove local *a priori* estimates in $L^2$ for Symm’s integral equation and in $H^1$ for the hyper-singular equation. The local rate of convergence is limited by the local regularity of the sought solution and the sum of the rates given by the global regularity and additional regularity provided by the shift theorem for a dual problem.

## Introduction

The boundary element method (BEM) for the discretization of boundary integral equations is an established numerical method for solving partial differential equations on (un)bounded domains. As an energy projection method, the Galerkin BEM is, like the finite element method (FEM), (quasi-)optimal in some global norm. However, often the quantity of interest is not the error on the whole domain, but rather a local error on part of the computational domain. For the FEM, the analysis of local errors goes back at least to [18]; advanced versions can be found in [10, 30]. For the Poisson problem, the local error estimates typically have the form1.1where *u* is the exact solution, $u_h$ the finite element approximation from a space $X_h$ of piecewise polynomials, and $B_0 \Subset B_1$ are open subsets of $\varOmega$ with $R{:}{=}\text {dist}(B_0,\partial B_1)$. Thus, the local error in the energy norm is bounded by the local best approximation on a larger domain and the error in the weaker $L^2$-norm. The local best approximation allows for convergence rates limited only by the local regularity; the $L^2$-error is typically controlled with a duality argument and limited by the regularity of the dual problem as well as the global regularity of the solution. Therefore, if the solution is smoother locally, we can expect better rates of convergence for the local error.

Significantly fewer works study the local behavior of the BEM. The case of smooth two dimensional curves is treated in [5, 21, 28], in [27] three dimensional screen problems are studied, and [14] discusses local error estimates on polygons. [19, 20] provide estimates in the $L^{\infty }$-norm on smooth domains. Local error estimators for the BEM are presented in [23]. However, for the case of piecewise smooth geometries such as polygonal and polyhedral domains, sharp local error estimates that exploit the maximal (local) regularity of the solution are not available. Moreover, the analyses of [14, 21, 27, 28] are tailored to the energy norm and do not provide optimal local estimates in stronger norms, whereas [5] imposes additional global regularity.

In this article, we obtain sharp local error estimates for lowest order discretizations on quasi-uniform meshes for Symm’s integral equation in the $L^2$-norm and for the (stabilized) hyper-singular integral equation in the $H^1$-seminorm on polygonal/polyhedral domains. Structurally, the local estimates are similar to (1.1): The local error is bounded by a local best approximation error and a global error in a weaker norm. More precisely, our local convergence rates depend only on the local regularity and the sum of the rates given by the global regularity and the additional regularity of the dual problem on polygonal/polyhedral domains. Numerical examples show the sharpness of our analysis. As discussed in Remark 2.5 below, our results improve [21, 27, 28] as estimates in $L^2$ (for Symm’s equation) and $H^1$ (for the hyper-singular equation) are obtained there from local energy norm estimates with the aid of inverse estimates, thereby leading to a loss of $h^{-1/2}$. In contrast, we avoid using an inverse inequality to go from the energy norm to a stronger norm.

The paper is structured as follows. We start with some notations and then present the main results for both Symm’s integral equation and the hyper-singular integral equation in Sect. 2. In Sects. 3 and 4 we are concerned with the proofs of these results. First, some technical preliminaries that exploit the additional regularity on piecewise smooth geometries to prove some improved *a priori* estimates for solutions of Poisson’s equation as well as for the boundary integral operators are presented. Then, we prove the main results, first for Symm’s equation, then for the stabilized hyper-singular equation. In principle, the proofs take ideas from [30], but important modifications of the arguments are necessary due to the nonlocal character of the integral operators. As in [30] a key ingredient are interior regularity estimates, which were provided recently in [11, 12], and to exploit some additional smoothing properties of commutators that arise in a localization step. Finally, Sect. 5 provides numerical examples that underline the sharpness of our theoretical local *a priori* estimates.

### Notation on norms

For domains $\omega \subset {\mathbb {R}}^d$, we define the integer order Sobolev spaces $H^k(\omega )$, $k \in {\mathbb {N}}_0$, in the standard way [15, p. 73ff]. The fractional Sobolev spaces $H^{k + s}(\omega )$, $k \in {\mathbb {N}}_0$, $s \in (0,1)$ are defined by the Slobodeckii norm as described in [15, p. 73ff]. For open sets $\omega = \cup _{i=1}^m \omega _i$ consisting of finitely many components of connectedness $\omega _i$, the Sobolev spaces $H^{k+s}(\omega )$ are understood in a piecewise way with norm $\Vert u\Vert ^2_{H^{k+s}(\omega )}= \sum _i \Vert u\Vert ^2_{H^{k+s}(\omega _i)}$. The spaces ${\widetilde{H}}^s(\omega )$, $s \ge 0$, consist of those function whose zero extension to ${\mathbb {R}}^d$ is in $H^s({\mathbb {R}}^d)$. The spaces $H^{-s}(\omega )$, $s \ge 0$, are taken to be the dual space of ${\widetilde{H}}^s(\omega )$. We will make use of the fact that for bounded Lipschitz domains $\omega$1.2For Lipschitz domains $\varOmega \subset {\mathbb {R}}^d$ with boundary $\varGamma {:}{=} \partial \varOmega$, we define Sobolev spaces $H^s(\varGamma )$ with $s \in [0,1]$ as described in [15, p. 96ff] using local charts. For $s > 1$, we *define* the spaces $H^s(\varGamma )$ in a non-standard way: $H^s(\varGamma )$ consists of those functions that have a lifting to $H^{1/2+s}({\mathbb {R}}^d)$, and we define the norm $\Vert \cdot \Vert _{H^s(\varGamma )}$ by

1.3 $$\begin{aligned} \left\| u \right\| _{H^{s}(\varGamma )}&:= \inf \{ \left\| v \right\| _{H^{1/2+s}({\mathbb {R}}^d)}\,:\, v\in H^{1/2+s}({\mathbb {R}}^d), v|_{\varGamma }=u \}. \end{aligned}$$

Correspondingly, for $s>1$ there is a lifting operator

1.4 $$\begin{aligned} {\mathcal {L}}:H^{s}(\varGamma )\rightarrow H^{1/2+s}({\mathbb {R}}^d) \end{aligned}$$

with the lifting property $({\mathcal {L}} u)|_{\varGamma } = u$, which is bounded by definition of the norm (1.3). The spaces $H^{-s}(\varGamma )$, $s \ge 0$, are the duals of $H^s(\varGamma )$. Their norm is defined as

$$\begin{aligned} \Vert u\Vert _{H^{-s}(\varGamma )}{:}{=} \sup _{v \in H^s(\varGamma )} \frac{\langle u,v\rangle }{\Vert v\Vert _{H^s(\varGamma )}}. \end{aligned}$$

#### Remark 1.1

(equivalent norm definitions)

(i) For $s > 1$ an equivalent definition of the norm $\Vert \cdot \Vert _{H^s(\varGamma )}$ in (1.3) would be to replace $\Vert \cdot \Vert _{H^{s+1/2}({\mathbb R}^d)}$ with $\Vert \cdot \Vert _{H^{s+1/2}(\varOmega )}$, i.e.,
  $$\begin{aligned} \left\| u \right\| _{H^{s}(\varGamma )} := \inf \{ \left\| v \right\| _{H^{1/2+s}(\varOmega )}\,:\, v\in H^{1/2+s}(\varOmega ), v|_{\varGamma }=u \}. \end{aligned}$$
  This follows from the existence of the universal extension operator $E:L^2(\varOmega ) \rightarrow L^2({\mathbb {R}}^d)$ described in [25, Chap. VI.3], which asserts that *E* is also a bounded linear operator $H^{k}(\varOmega ) \rightarrow H^k({\mathbb {R}}^d)$ for any $k \ge 0$.
(ii) The trace operator $\gamma _0: H^{s+1/2}({\mathbb {R}}^d) \rightarrow H^{s}(\varGamma )$ is a continuous operator for $0< s < 1$ (cf. [15, Thm. 3.38], [22, Thm. 2.6.8], [17, Thm. 2.3]). [22, Thm. 2.6.11] (cf. also [15, Thm. 3.37], [17, Lem. 2.6]) assert the existence of a continuous lifting ${\mathcal {L}}$ in the range $0< s < 1$ as well so that (1.3) is an equivalent norm for $0< s < 1$ as well.
(iii) For polygonal (in 2D) and polyhedral (in 3D) Lipschitz domains the spaces $H^{s}(\varGamma )$ in the range $s \in (1,3/2)$ can be characterized alternatively as follows: Let $\varGamma _i$, $i=1,\ldots ,N$, be the affine pieces of $\varGamma$, which may be identified with an interval (for the 2D case) or a polygon (for the 3D case). Then
  1.5 $$\begin{aligned} u \in H^s(\varGamma ) \quad \Longleftrightarrow \quad u|_{\varGamma _i} \in H^s(\varGamma _i) \quad \forall i \in \{1,\ldots ,N\} \quad \text{ and } u \in C^0(\varGamma ). \end{aligned}$$
  The equivalence (1.5) gives rise to yet another norm equivalence for the space $H^s(\varGamma )$, namely, $\Vert u\Vert _{H^s(\varGamma )} \sim \sum _{i=1}^N \Vert u\Vert _{H^s(\varGamma _i)}$. The condition $u \in C^0(\varGamma )$ is a *compatibility* condition. More generally, for $s > 3/2$ similar, more complicated compatibility conditions can be formulated to describe the space $H^s(\varGamma )$ in terms of piecewise Sobolev spaces. $\square$

We will also need local norms on the boundary. For an open subset $\varGamma _0 \subset \varGamma$ and $s \ge 0$, we define local negative norms by1.6In the following, we write $\gamma _0^\mathrm{int}$ for the interior trace operator, i.e., the trace operator from the inside of the domain and $\gamma _0^\mathrm{ext}$ for the exterior trace operator. For the jump of the trace of a function *u* we use the notation $[\gamma _0 u] {:}{=} \gamma _0^\mathrm{ext} u-\gamma _0^\mathrm{int} u$. In order to shorten notation, we write $\gamma _0$ for the trace, if the interior and exterior trace are equal, i.e., $[\gamma _0 u]=0$. We denote the interior and exterior conormal derivative by $\gamma _1^\mathrm{int} u {:}{=} \gamma _0^\mathrm{int}\nabla u \cdot n$, $\gamma _1^\mathrm{ext} u {:}{=} \gamma _0^\mathrm{ext}\nabla u \cdot n$, where *n* denotes the normal vector pointing into ${\mathbb R}^d {\setminus } \varOmega$. The jump of the normal derivative across the boundary is defined by $[\partial _n u]{:}{=} \gamma _1^\mathrm{ext} u- \gamma _1^\mathrm{int} u$, and we write $\partial _n u$ for the normal derivative if $[\partial _n u] = 0$.

We will call axis-parallel squares/cubes “boxes”.

## Main results

We study bounded Lipschitz domains $\varOmega \subset {\mathbb {R}}^d$, $d \ge 2$ with *polygonal/polyhedral boundary* $\varGamma :=\partial \varOmega$.

### Symm’s integral equation

The elliptic shift theorem for the Dirichlet problem is valid in a range that is larger than for general Lipschitz domains. We characterize this extended range by a parameter $\alpha _D \in (0,1/2)$ that will pervade most of the estimates of the present work. It is defined by the following assumption:

#### Assumption 2.1

$\varOmega \subset {\mathbb {R}}^d$, $d\ge 2$, is a bounded Lipschitz domain whose boundary consists of finitely many affine pieces (i.e., $\varOmega$ is the intersection of finitely many half-spaces). $R_\varOmega > 0$ is such that the open ball $B_{R_{\varOmega }}(0) \subset {\mathbb {R}}^d$ of radius $R_\varOmega$ that is centered at the origin contains ${\overline{\varOmega }}$. The parameter $\alpha _D \in (0,1/2)$ is such that for every $\varepsilon \in (0,\alpha _D]$ there is $C_\varepsilon > 0$ such that the *a priori* bound

2.1 $$\begin{aligned} \left\| T f \right\| _{H^{3/2+\varepsilon }(B_{R_{\varOmega }}(0)\backslash \varGamma )}\le C_\varepsilon \left\| f \right\| _{H^{-1/2+\varepsilon }(B_{R_{\varOmega }}(0)\backslash \varGamma )} \qquad \forall f \in H^{-1/2+\varepsilon }(B_{R_\varOmega }(0){\setminus }\varGamma ) \end{aligned}$$

holds, where $u{:}{=} Tf \in H^1(B_{R_\varOmega }(0){\setminus }\varGamma )$ denotes the solution of

2.2 $$\begin{aligned} -\varDelta u = f \quad \text{ in } \quad B_{R_\varOmega }(0){\setminus }\varGamma , \quad \gamma _0 u = 0 \quad \text{ on } \quad \varGamma \cup \partial B_{R_\varOmega }(0). \end{aligned}$$

Recall that the norms $\left\| \cdot \right\| _{H^{s}(B_{R_{\varOmega }}(0)\backslash \varGamma )}$, $s>0$ are understood as the sum of the norm on $\varOmega$ and $B_{R_{\varOmega }}(0)\backslash \overline{\varOmega }$, i.e.,

$$\begin{aligned} \left\| u \right\| _{H^{s}(B_{R_{\varOmega }}(0)\backslash \varGamma )}^2{:}{=}\left\| u \right\| _{H^{s} (\varOmega )}^2+ \left\| u \right\| _{H^{s}(B_{R_{\varOmega }}(0)\backslash \overline{\varOmega })}^2. \end{aligned}$$

#### Remark 2.2

The condition on the parameter $\alpha _D$ in Assumption 2.1 can be described in terms of two Dirichlet problems, one posed on $\varOmega$ and one posed on $B_{R_\varOmega }(0) {\setminus } {\overline{\varOmega }}$. For each of these two domains, a shift theorem is valid, and $\alpha _D$ is determined by the more stringent of the two conditions. It is worth stressing that the type of boundary condition on $\partial B_{R_{\varOmega }}(0)$ is not essential in view of the smoothness of $\partial B_{R_{\varOmega }}(0)$ and .

In the case $d = 2$ the parameter $\alpha _D$ is determined by the extremal angles of the polygon $\varOmega$. Specifically, let $0< \omega _j < 2 \pi$, $j=1,\ldots ,J$, be the interior angles of the polygon $\varOmega$. Then, Assumption 2.1 is valid for any $\alpha _D >0$ that satisfies

$$\begin{aligned} \frac{1}{2}< \frac{1}{2}+\alpha _D< \min _{j=1,\ldots ,J} \min \left\{ \frac{\pi }{\omega _j}, \frac{\pi }{2\pi -\omega _j}\right\} < 1. \end{aligned}$$

(Note that $\omega _j \ne \pi$ for all *j* so that the right inequality is indeed strict.) $\square$

We consider Symm’s integral equation in its weak form: Given $f \in H^{1/2}(\varGamma )$ find $\phi \in H^{-1/2}(\varGamma )$ such that

2.3 $$\begin{aligned} \left\langle V\phi ,\psi \right\rangle _{L^2(\varGamma )} = \left\langle f,\psi \right\rangle _{L^2(\varGamma )} \quad \forall \psi \in H^{-1/2}(\varGamma ). \end{aligned}$$

Here, the single-layer operator *V* is given by

$$\begin{aligned} V\phi (x) = \int _{\varGamma }G(x,y)\phi (y) ds_y, \quad x \in \varGamma , \end{aligned}$$

where, with the surface measure $|S^{d-1}|$ of the Euclidean sphere in ${\mathbb {R}}^d$, we set

2.4 $$\begin{aligned} G(x,y) = {\left\{ \begin{array}{ll} -\frac{1}{|S^1|}\,\log |x-y|,&{}\quad \text {for }d=2,\\ +\frac{1}{|S^{d-1}|}\,|x-y|^{-(d-2)}, &{}\quad \text {for }d\ge 3. \end{array}\right. } \end{aligned}$$

The single layer operator *V* is a bounded linear operator in $L(H^{-1/2+s}(\varGamma ),H^{1/2+s}(\varGamma ))$ for $\left| s \right| \le \frac{1}{2}$, [22, Thm. 3.1.16]. It is elliptic for $s=0$ with the usual proviso for $d=2$ that , which we may assume by scaling.

Let $\mathcal {T}_h = \{T_1,\dots ,T_N\}$ be a quasi-uniform, regular and $\gamma$-shape regular triangulation of the boundary $\varGamma$ with mesh-width . By $S^{0,0}(\mathcal {T}_h){:}{=}\{u \in L^2(\varGamma ):u|_{T_j}\; \text {is constant} \,\forall T_j \in \mathcal {T}_h\}$ we denote the space of piecewise constant functions on the mesh $\mathcal {T}_h$. The Galerkin formulation of (2.3) reads: Find $\phi _h \in S^{0,0}(\mathcal {T}_h)$ such that

2.5 $$\begin{aligned} \left\langle V\phi _h,\psi _h \right\rangle _{L^2(\varGamma )} = \left\langle f,\psi _h \right\rangle _{L^2(\varGamma )} \quad \forall \psi _h \in S^{0,0}(\mathcal {T}_h). \end{aligned}$$

The following theorem is one of the main results of this paper. It estimates the Galerkin error in the $L^2$-norm on a subdomain by the local best approximation error in $L^2$ on a slightly larger subdomain and the global error in a weaker norm.

#### Theorem 2.3

Let Assumption 2.1 hold and let $\mathcal {T}_h$ be a quasi-uniform, $\gamma$-shape regular triangulation. Let $\phi \in H^{-1/2}(\varGamma )$ and $\phi _h \in S^{0,0}(\mathcal {T}_h)$ satisfy the Galerkin orthogonality condition

2.6 $$\begin{aligned} \langle V( \phi - \phi _h),\psi _h\rangle _{L^2(\varGamma )}= 0 \qquad \forall \psi _h \in S^{0,0}(\mathcal {T}_h). \end{aligned}$$

Let $\varGamma _0$, ${\widehat{\varGamma }}$ be open subsets of $\varGamma$ with $\varGamma _0\subset {\widehat{\varGamma }} \subsetneq \varGamma$ and . Let *h* be such that $C_{\alpha _D}\frac{h}{R}\le \frac{1}{12}$ with a fixed constant $C_{\alpha _D}$ depending only on $\alpha _D$. Assume that $\phi \in L^{2}({\widehat{\varGamma }})$. Then, we have

$$\begin{aligned} \left\| \phi -\phi _h \right\| _{L^{2}(\varGamma _0)} \le C\left( \inf _{\chi _h\in S^{0,0}(\mathcal {T}_h)}\left\| \phi -\chi _h \right\| _{L^{2}({\widehat{\varGamma }})} + \left\| \phi -\phi _h \right\| _{H^{-1-\alpha _D}(\varGamma )}\right) . \end{aligned}$$

The constant $C>0$ depends only on $\varGamma ,\varGamma _0,{\widehat{\varGamma }},d,R,$ and the $\gamma$-shape regularity of $\mathcal {T}_h$.

If we additionally assume higher local regularity as well as some (low) global regularity of the solution $\phi$, this local estimate implies that the local error converges faster than the global error, which is stated in the following corollary.

#### Corollary 2.4

Let the assumptions of Theorem 2.3 be fulfilled. Let $\widetilde{\varGamma }\subset \varGamma$ be a subset with ${\widehat{\varGamma }}\subsetneq \widetilde{\varGamma }$ and . Additionally, assume $\phi \in H^{-1/2+\alpha }(\varGamma ) \cap H^{\beta }(\widetilde{\varGamma })$ with $\alpha \ge 0$, $\beta \in [0,1]$. Then, we have

$$\begin{aligned} \left\| \phi -\phi _h \right\| _{L^{2}(\varGamma _0)} \le C h^{\min \{1/2+\alpha +\alpha _D,\beta \}} \end{aligned}$$

with a constant $C>0$ depending only on $\varGamma ,\varGamma _0,{\widehat{\varGamma }},\widetilde{\varGamma },d,R,\alpha ,\beta$, and the $\gamma$-shape regularity of $\mathcal {T}_h$.

In the results of [18, 30] singularities far from the domain of interest still have a weak influence on the local convergence of the FEM. Corollary 2.4 shows that this is similar in the BEM: The *a priori* estimate shows the effect of singularities of the solution (represented by $\alpha$) and those induced by the geometry (represented by $\alpha _D$) affect the local convergence.

#### Remark 2.5

In comparison to [27], Corollary 2.4 gives a better result for the rate of convergence of the local error in the case where the convergence is limited by the global error in the weaker norm. More precisely, for the case $\phi \in H^{1/2}(\widetilde{\varGamma }) \cap L^2(\varGamma )$, [27] obtains the local rate of 1 / 2, which coincides with our local rate. However, if $\phi \in H^{1}(\widetilde{\varGamma })$, we obtain rate 1 in the $L^2$-norm, whereas the rate in [27] remains 1 / 2. $\square$

#### Remark 2.6

Even for smooth functions *f*, the solution $\phi$ of (2.3) is, in general, not better than $H^{\alpha }(\varGamma )$ with $\alpha = \frac{1}{2} + \alpha _D$. Recall from Remark 2.2 that $\alpha _D$ is determined by the mapping properties for *both* the interior and the exterior Dirichlet problem. A special situation therefore arises if Symm’s integral equation is obtained from reformulating an interior (or exterior) Dirichlet problem. To be specific, consider again the case $d = 2$ of a polygon $\varOmega$ with interior angles $\omega _j$, $j=1,\ldots ,J$. We rewrite the boundary value problem $-\varDelta u = 0$ in $\varOmega$ with $u|_\varGamma = g$ as the integral equation

$$\begin{aligned} V \phi = \left( \frac{1}{2} + K\right) g \end{aligned}$$

for the unknown function $\phi = \partial _n u$ with the double layer operator *K* defined by

$$\begin{aligned} K\phi (x){:}{=}\int _{\varGamma }\partial _{n_y} G(x,y)\phi (y)ds_y. \end{aligned}$$

Then, $\phi \in H^\alpha (\varGamma )$ for any $\alpha$ with $\alpha < 1/2 + \min _j \frac{\pi }{\omega _j}$. $\square$

### The hyper-singular integral equation

For the Neumann problem, we assume an extended shift theorem as well.

#### Assumption 2.7

$\varOmega \subset {\mathbb {R}}^d$, $d\ge 2$, is a bounded Lipschitz domain whose boundary consists of finitely many affine pieces (i.e., $\varOmega$ is the intersection of finitely many half-spaces). $R_\varOmega > 0$ is such that the open ball $B_{R_{\varOmega }}(0) \subset {\mathbb {R}}^d$ contains ${\overline{\varOmega }}$. The parameter $\alpha _N \in (0,\alpha _D]$, where $\alpha _D$ is the parameter from Assumption 2.1, is such that for every $\varepsilon \in (0,\alpha _N]$ there is $C_\varepsilon > 0$ such that for all $f \in H^{-1/2+\varepsilon }(B_{R_\varOmega }(0){\setminus }\varGamma )$ and $g \in H^{\varepsilon }(\varGamma )$ with $\int _{\varOmega }f +\int _{\varGamma } g = 0$ the *a priori* bound

2.7 $$\begin{aligned} \left\| T f \right\| _{H^{3/2+\varepsilon }(B_{R_{\varOmega }}(0)\backslash \varGamma )}\le C_\varepsilon \left( \left\| f \right\| _{H^{-1/2+\varepsilon }(B_{R_{\varOmega }}(0)\backslash \varGamma )} + \left\| g \right\| _{H^{\varepsilon }(\varGamma )} \right) \end{aligned}$$

holds, where $u{:}{=} Tf \in H^1(B_{R_\varOmega }(0){\setminus }\varGamma )$ denotes the solution of

$$\begin{aligned} -\varDelta u&= f \quad \text{ in } \varOmega , \qquad&\gamma _1^\mathrm{int} u = g \quad \text{ on } \varGamma ,&\qquad \left\langle u,1 \right\rangle _{L^2(\varOmega )} = 0,\\ -\varDelta u&= f \quad \hbox {in } B_{R_{\varOmega }}(0)\backslash \overline{\varOmega }, \qquad&\gamma _1^\mathrm{ext} u = g \quad \hbox {on }\varGamma ,&\qquad \gamma _0^\mathrm{int} u = 0 \quad \hbox {on } \partial B_{R_{\varOmega }}(0). \end{aligned}$$

The condition on the parameter $\alpha _N$ again can be described in terms of two problems, a pure Neumann problem posed in $\varOmega$, for which we need a compatibility condition, and a mixed Dirichlet–Neumann problem posed on $B_{R_\varOmega }(0) \backslash \overline{\varOmega }$, which is uniquely solvable without the need to impose a solvability condition for *f*, *g*.

The parameter $\alpha _N$ again depends only on the geometry and the corners/edges that induce singularities. In fact, on polygonal domains, i.e., $d=2$, $\alpha _D = \alpha _N$, see, e.g., [9].

Studying the inhomogeneous Neumann boundary value problem $-\varDelta u = 0$, $\partial _n u = g$, leads to the boundary integral equation of finding $\varphi \in H^{1/2}(\varGamma )$ such that $W\varphi = f$ with $f \in H^{-1/2}(\varGamma )$ satisfying the compatibility condition $\left\langle f,1 \right\rangle _{L^2(\varGamma )} = 0$, and the hyper-singular integral operator $W \in L(H^{1/2}(\varGamma ),H^{-1/2}(\varGamma ))$ defined by

$$\begin{aligned} W\varphi (x) = -\partial _{n_x}\int _{\varGamma }\partial _{n_y}G(x,y)\varphi (y) ds_y, \quad x \in \varGamma . \end{aligned}$$

We additionally assume that $\varGamma$ is connected, so that the hyper-singular integral operator has a kernel of dimension one consisting of the constant functions. Therefore, the boundary integral equation is not uniquely solvable. Employing the constraint $\left\langle \varphi ,1 \right\rangle _{L^2(\varGamma )} = 0$ leads to the stabilized variational formulation

2.8 $$\begin{aligned} \left\langle W\varphi ,\psi \right\rangle _{L^2(\varGamma )} + \left\langle \varphi ,1 \right\rangle _{L^2(\varGamma )}\left\langle \psi ,1 \right\rangle _{L^2(\varGamma )} = \left\langle f,\psi \right\rangle _{L^2(\varGamma )} \quad \forall \psi \in H^{1/2}(\varGamma ), \end{aligned}$$

which has a unique solution $\varphi \in H^{1/2}(\varGamma )$, see, e.g., [26]. For the Galerkin discretization we employ lowest order test and trial functions in $S^{1,1}(\mathcal {T}_h){:}{=}\{u \in H^1(\varGamma ):u|_{T_j} \in \mathcal {P}_1 \,\forall T_j \in \mathcal {T}_h\}$, which leads to the discrete variational problem of finding $\psi _h \in S^{1,1}(\mathcal {T}_h)$ such that

2.9 $$\begin{aligned} \left\langle W\varphi _h,\psi _h \right\rangle _{L^2(\varGamma )} + \left\langle \varphi _h,1 \right\rangle _{L^2(\varGamma )}\left\langle \psi _h,1 \right\rangle _{L^2(\varGamma )} = \left\langle f,\psi _h \right\rangle _{L^2(\varGamma )} \quad \forall \psi _h \in S^{1,1}(\mathcal {T}_h). \end{aligned}$$

The following theorem is the analog of Theorem 2.3 for the hyper-singular integral equation. The local error in the $H^1$-seminorm is estimated by the local best approximation error and the global error in a weak norm.

#### Theorem 2.8

Let Assumption 2.7 hold and let $\mathcal {T}_h$ be a quasi-uniform, $\gamma$-shape regular triangulation. Let $\varphi \in H^{1/2}(\varGamma )$ and $\varphi _h \in S^{1,1}(\mathcal {T}_h)$ satisfy the Galerkin orthogonality condition

2.10 $$\begin{aligned} \langle W( \varphi - \varphi _h),\psi _h\rangle _{L^2(\varGamma )} + \left\langle \varphi -\varphi _h,1 \right\rangle _{L^2(\varGamma )}\left\langle \psi _h,1 \right\rangle _{L^2(\varGamma )}= 0 \qquad \forall \psi _h \in S^{1,1}(\mathcal {T}_h). \end{aligned}$$

Let $\varGamma _0$, ${\widehat{\varGamma }}$ be open subsets of $\varGamma$ with $\varGamma _0\subset {\widehat{\varGamma }} \subsetneq \varGamma$ and . Let *h* be such that $C_{\alpha _N}\frac{h}{R}\le \frac{1}{12}$ with a fixed constant $C_{\alpha _N}$ depending only on $\alpha _N$. Assume that $\varphi \in H^{1}({\widehat{\varGamma }})$. Then, we have

$$\begin{aligned} \left\| \varphi -\varphi _h \right\| _{H^{1}(\varGamma _0)} \le C \left( \inf _{\chi _h\in S^{1,1}(\mathcal {T}_h)} \left\| \varphi - \chi _h \right\| _{H^{1}({\widehat{\varGamma }})} + \left\| \varphi -\varphi _h \right\| _{H^{-\alpha _N}(\varGamma )}\right) . \end{aligned}$$

The constant $C>0$ depends only on $\varGamma ,\varGamma _0,{\widehat{\varGamma }},d,R,$ and the $\gamma$-shape regularity of $\mathcal {T}_h$.

Again, assuming additional regularity, the local estimate of Theorem 2.8 leads to a higher rate of local convergence of the BEM for the stabilized hyper-singular integral equation.

#### Corollary 2.9

Let the assumptions of Theorem 2.8 be fulfilled. Let $\widetilde{\varGamma }\subset \varGamma$ be a subset with ${\widehat{\varGamma }}\subsetneq \widetilde{\varGamma }$, . Additionally, assume $\varphi \in H^{1/2+\alpha }(\varGamma ) \cap H^{1+\beta }(\widetilde{\varGamma })$ with $\alpha \ge 0$, $\beta \in [0,1]$. Then, we have

$$\begin{aligned} \left\| \varphi -\varphi _h \right\| _{H^{1}(\varGamma _0)} \le C h^{\min \{1/2+\alpha +\alpha _N,\beta \}} \end{aligned}$$

with a constant $C>0$ depending only on $\varGamma ,\varGamma _0,{\widehat{\varGamma }},\widetilde{\varGamma },d,R,\alpha ,\beta$, and the $\gamma$-shape regularity of $\mathcal {T}_h$.

## Shift theorems

The following two sections are dedicated to the proofs of Theorem 2.3 and Corollary 2.4 for Symm’s integral equation as well as Theorem 2.8 and Corollary 2.9 for the hyper-singular integral equation. We start with some technical results that are direct consequences of the assumed shift theorems from Assumption 2.1 for the Dirichlet problem and Assumption 2.7 for the Neumann problem. The shift theorem of Assumption 2.1 implies the following shift theorem for Dirichlet problems:

### Lemma 3.1

Let the shift theorem from Assumption 2.1 hold and let *u* be the solution of the inhomogeneous Dirichlet problem $-\varDelta u = 0$ in $B_{R_{\varOmega }}(0)\backslash \varGamma$, $\gamma _0 u = g$ on $\varGamma \cup \partial B_{R_{\varOmega }}(0)$ for some $g\in H^{1/2}(\varGamma \cup \partial B_{R_{\varOmega }}(0))$.

(i) There is a constant $C>0$ depending only on $\varOmega$ and $\alpha _D$ such that
  3.1 $$\begin{aligned} \left\| u \right\| _{H^{1/2-\alpha _D}(B_{R_{\varOmega }}(0)\backslash \varGamma )}\le C \left\| g \right\| _{H^{-\alpha _D}(\varGamma \cup \partial B_{R_{\varOmega }}(0))}. \end{aligned}$$
(ii) Let $\varepsilon \in (0,\alpha _D]$ and $B\subset B' \subset B_{R_{\varOmega }}(0)$ be nested subdomains with . Let $\eta \in C^{\infty }({\mathbb {R}}^d)$ be a cut-off function satisfying $\eta \equiv 1$ on $B\cap \varGamma$, , and  for $k \in \{0,1,2\}$. Assume $\eta g\in H^{1+\varepsilon }(\varGamma )$. Then
  3.2 $$\begin{aligned} \left\| u \right\| _{H^{3/2+\varepsilon }(B\backslash \varGamma )} \le C\left( \left\| u \right\| _{H^1(B'\backslash \varGamma )} +\left\| \eta g \right\| _{H^{1+\varepsilon }(\varGamma )}\right) . \end{aligned}$$
  Here, the constant $C>0$ additionally depends on .

### Proof:

*Proof of (i):* Let *v* solve $-\varDelta v = w$ in $B_{R_{\varOmega }}(0)\backslash \varGamma$, $\gamma _0 v = 0$ on $\varGamma \cup \partial B_{R_{\varOmega }}(0)$ for $w \in H^{-1/2+\alpha _D}(B_{R_{\varOmega }}(0)\backslash \varGamma )$. Then, in view of (1.2), we have

$$\begin{aligned} \left\| u \right\| _{H^{1/2-\alpha _D}(B_{R_{\varOmega }}(0)\backslash \varGamma )}= & {} \sup _{w \in H^{-1/2+\alpha _D}(B_{R_{\varOmega }}(0)\backslash \varGamma )} \frac{\left\langle u,w \right\rangle _{L^2(B_{R_{\varOmega }}(0)\backslash \varGamma )}}{\left\| w \right\| _{H^{-1/2+\alpha _D}(B_{R_{\varOmega }}(0)\backslash \varGamma )}} \\= & {} \sup _{w \in H^{-1/2+\alpha _D}(B_{R_{\varOmega }}(0)\backslash \varGamma )} \frac{-\left\langle u,\varDelta v \right\rangle _{L^2(B_{R_{\varOmega }}(0)\backslash \varGamma )}}{\left\| w \right\| _{H^{-1/2+\alpha _D}(B_{R_{\varOmega }}(0)\backslash \varGamma )}}. \end{aligned}$$

Integration by parts on $\varOmega$ and $B_{R_{\varOmega }}(0)\backslash \overline{\varOmega }$ and the boundary condition $\gamma _0 v = 0$ lead to

$$\begin{aligned} \left\langle u,\varDelta v \right\rangle _{L^2(B_{R_{\varOmega }}(0)\backslash \varGamma )}= & {} \left\langle \varDelta u,v \right\rangle _{L^2(B_{R_{\varOmega }}(0)\backslash \varGamma )} + \left\langle \gamma _0 u,[\partial _n v] \right\rangle _{L^2(\varGamma )} \\&+ \left\langle \gamma _0 u,\partial _n v \right\rangle _{L^2(\partial B_{R_{\varOmega }}(0))} \\= & {} \left\langle g,[\partial _n v] \right\rangle _{L^2(\varGamma )} + \left\langle g,\partial _n v \right\rangle _{L^2(\partial B_{R_{\varOmega }}(0))}. \end{aligned}$$

We split the polygonal/polyhedral boundary $\varGamma = \bigcup _{\ell =1}^m \overline{\varGamma _{\ell }}$ into its (smooth) faces $\varGamma _{\ell }$ and prolong each face $\varGamma _{\ell }$ to the hyperplane $\varGamma _{\ell }^{\infty }$, which decomposes ${\mathbb {R}}^d$ into two half spaces $\varOmega _{\ell }^{\pm }$. Let $\chi _{\ell } \in L^2(\varGamma )$ be the characteristic function for $\varGamma _{\ell }$. Since the normal vector on a face does not change, we may use the trace estimate (note: $0<\alpha _D < 1/2$) facewise, to estimate

3.3 $$\begin{aligned} \left\| [\partial _n v] \right\| _{H^{\alpha _D}(\varGamma )}\lesssim & {} \sum _{\ell =1}^m\left\| \chi _{\ell }[\nabla v \cdot n] \right\| _{H^{\alpha _D}(\varGamma _{\ell })} \lesssim \sum _{\ell =1}^m \left\| \nabla v \right\| _{H^{1/2+\alpha _D}(\varOmega _{\ell }^{\pm }\cap B_{R_{\varOmega }}(0))}\nonumber \\\lesssim & {} \left\| v \right\| _{H^{3/2+\alpha _D}(B_{R_{\varOmega }}(0)\backslash \varGamma )}. \end{aligned}$$

As the boundary $\partial B_{R_{\varOmega }}(0)$ is smooth, standard elliptic regularity yields $\left\| \partial _n v \right\| _{H^{\alpha _D}(\partial B_{R_{\varOmega }}(0))} \lesssim \left\| v \right\| _{H^{3/2+\alpha _D}(B_{R_{\varOmega }}(0)\backslash \varGamma )}$. This leads to

$$\begin{aligned}&\left\| u \right\| _{H^{1/2-\alpha _D}(B_{R_{\varOmega }}(0)\backslash \varGamma )} \lesssim \sup _{w \in H^{-1/2+\alpha _D}(B_{R_{\varOmega }}(0)\backslash \varGamma )} \frac{\left| \left\langle g,[\partial _n v] \right\rangle _{L^2(\varGamma )}+\left\langle g,\partial _n v \right\rangle _{L^2(\partial B_{R_{\varOmega }}(0))} \right| }{\left\| w \right\| _{H^{-1/2+\alpha _D}(B_{R_{\varOmega }}(0)\backslash \varGamma )}}\\&\qquad \quad \lesssim \sup _{w\in H^{-1/2+\alpha _D}(B_{R_{\varOmega }}(0)\backslash \varGamma )} \frac{\left\| g \right\| _{H^{-\alpha _D}(\varGamma \cup \partial B_{R_{\varOmega }}(0))}\left( \left\| [\partial _n v] \right\| _{H^{\alpha _D}(\varGamma )}+ \left\| \partial _n v \right\| _{H^{\alpha _D}(\partial B_{R_{\varOmega }}(0))}\right) }{\left\| w \right\| _{H^{-1/2+\alpha _D}(B_{R_{\varOmega }}(0)\backslash \varGamma )}} \\&\qquad \quad \lesssim \left\| g \right\| _{H^{-\alpha _D}(\varGamma \cup \partial B_{R_{\varOmega }}(0))} \sup _{w \in H^{-1/2+\alpha _D}(B_{R_{\varOmega }}(0)\backslash \varGamma )} \frac{\left\| v \right\| _{H^{3/2+\alpha _D}(B_{R_{\varOmega }}(0)\backslash \varGamma )}}{\left\| w \right\| _{H^{-1/2+\alpha _D}(B_{R_{\varOmega }}(0)\backslash \varGamma )}}\\&\qquad \quad {\mathop {\lesssim }\limits ^{\text {Ass.}~2.1}} \left\| g \right\| _{H^{-\alpha _D}(\varGamma \cup \partial B_{R_{\varOmega }}(0))}. \end{aligned}$$

*Proof of (ii):* With the lifting operator ${\mathcal {L}}:H^{1+\varepsilon }(\varGamma )\rightarrow H^{3/2+\varepsilon }(B_{R_{\varOmega }}(0)\backslash \varGamma )$ from (1.4), the function ${\widetilde{u}}{:}{=}\eta ^2 u - \eta {\mathcal {L}}(\eta g)$ satisfies

$$\begin{aligned} -\varDelta {\widetilde{u}}&= -4\eta \nabla \eta \cdot \nabla u - (\varDelta \eta ^2) u+ \varDelta (\eta {\mathcal {L}}(\eta g))&\; \text {in}\; B_{R_{\varOmega }}(0)\backslash \varGamma , \\ \gamma _0{\widetilde{u}}&= 0&\; \text {on}\; \varGamma \cup \partial B_{R_{\varOmega }}(0). \end{aligned}$$

With the shift theorem from Assumption 2.1 we get

$$\begin{aligned}&\left\| u \right\| _{H^{3/2+\varepsilon }(B\backslash \varGamma )} \le \left\| \eta ^2 u \right\| _{H^{3/2+\varepsilon }(B\backslash \varGamma )}\\&\quad \le \left\| {\widetilde{u}} \right\| _{H^{3/2+\varepsilon }(B_{R_{\varOmega }}(0)\backslash \varGamma )}+ \left\| \eta {\mathcal {L}}(\eta g) \right\| _{H^{3/2+\varepsilon }(B_{R_{\varOmega }}(0)\backslash \varGamma )} \\&\quad \lesssim \left\| 4\eta \nabla \eta \cdot \nabla u + (\varDelta \eta ^2) u \right\| _{L^2(B_{R_{\varOmega }}(0)\backslash \varGamma )} + \left\| \varDelta (\eta {\mathcal {L}}(\eta g)) \right\| _{H^{-1/2+\varepsilon }(B_{R_{\varOmega }}(0)\backslash \varGamma )} \\&\qquad \qquad \qquad \qquad \qquad \qquad \qquad \qquad \qquad \qquad \qquad + \left\| {\mathcal {L}}(\eta g) \right\| _{H^{3/2+\varepsilon }(B_{R_{\varOmega }}(0)\backslash \varGamma )} \\&\quad \lesssim \left\| u \right\| _{H^1(B'\backslash \varGamma )} +\quad \left\| {\mathcal {L}}(\eta g) \right\| _{H^{3/2+\varepsilon }(B_{R_{\varOmega }}(0)\backslash \varGamma )} \lesssim \left\| u \right\| _{H^1(B'\backslash \varGamma )} + \left\| \eta g \right\| _{H^{1+\varepsilon }(\varGamma )}, \end{aligned}$$

which proves the second statement. $\square$

The following lemma collects mapping properties of the single-layer operator *V* that exploits the present setting of piecewise smooth geometries:

### Lemma 3.2

Define the *single layer potential* ${\widetilde{V}}$ by

3.4 $$\begin{aligned} {\widetilde{V}}{\phi }(x) {:}{=} \int _{\varGamma }G(x,y){\phi }(y) ds_y, \qquad x \in {\mathbb {R}}^d {\setminus }\varGamma . \end{aligned}$$

(i) The single layer potential ${\widetilde{V}}$ is a bounded linear operator from $H^{-1/2+s}(\varGamma )$ to $H^{1+s}(B_{R_\varOmega }(0)\backslash \varGamma )$ for $-1/2\le s < 1$.
(ii) The single-layer operator *V* is a bounded linear operator from $H^{-1/2+s}(\varGamma )$ to $H^{1/2+s}(\varGamma )$ for $-1/2 \le s < 1$.
(iii) The adjoint double-layer operator $K'$ is a bounded linear operator from $H^{-1/2+s}(\varGamma )$ to $H^{-1/2+s}(\varGamma )$ for $-1/2 \le s < 1$.

### Proof:

*Proof of (i):* The case $s \in (-1/2,1/2)$ is shown in [22, Thm. 3.1.16], and for $s=-\frac{1}{2}$ we refer to [29]. For the case $s \in [1/2,1)$, we exploit that $\varGamma$ is piecewise smooth. We split the polygonal/polyhedral boundary $\varGamma = \bigcup _{\ell =1}^m \overline{\varGamma _{\ell }}$ into its (smooth) faces $\varGamma _{\ell }$. Let $\chi _{\ell } \in L^2(\varGamma )$ be the characteristic function for $\varGamma _{\ell }$. Then, for $\varphi \in H^{-1/2+s}(\varGamma )$, we have ${\widetilde{V}} \varphi =\sum _{\ell =1}^m{\widetilde{V}}(\chi _{\ell }\varphi )$. We prolong each face $\varGamma _{\ell }$ to the hyperplane $\varGamma _{\ell }^{\infty }$, which decomposes ${\mathbb {R}}^d$ into two half spaces $\varOmega _{\ell }^{\pm }$. Due to $s <1$, we have $\chi _{\ell }\varphi \in H^{-1/2+s}(\varGamma _{\ell }^{\infty })$. Since the half spaces $\varOmega _{\ell }^{\pm }$ have smooth boundaries, we may use the mapping properties of ${\widetilde{V}}$ on smooth geometries, see, e.g., [15, Thm. 6.13] to estimate

$$\begin{aligned} \left\| {\widetilde{V}} \varphi \right\| _{H^{1+s}(B_{R_\varOmega }(0){\setminus }\varGamma )}\lesssim & {} \sum _{\ell =1}^m\left\| {\widetilde{V}}(\chi _{\ell }\varphi ) \right\| _{H^{1+s} (\varOmega _{\ell }^{\pm })} \\\lesssim & {} \sum _{\ell =1}^m\left\| \chi _{\ell }\varphi \right\| _{H^{-1/2+s} (\varGamma _{\ell }^{\infty })}\lesssim \left\| \varphi \right\| _{H^{-1/2+s}(\varGamma )}. \end{aligned}$$

*Proof of (ii):* The case $-1/2 \le s \le 1/2$ is taken from [22, Thm. 3.1.16]. For $s \in (1/2,1)$ the result follows from part (i) and the definition of the norm $\Vert \cdot \Vert _{H^s(\varGamma )}$ given in (1.3).

*Proof of (iii):* The case $-1/2 \le s \le 1/2$ is taken from [22, Thm. 3.1.16]. With $K' = \partial _n {\widetilde{V}}-\frac{1}{2}\mathrm {Id}$ the case $s \in (1/2,1)$ follows from part (i) and a facewise trace estimate (3.3) since

$$\begin{aligned} \left\| \partial _n {\widetilde{V}}\varphi \right\| _{H^{-1/2+s}(\varGamma )} \lesssim \left\| {\widetilde{V}}\varphi \right\| _{H^{1+s}(\varOmega )} \lesssim \left\| \varphi \right\| _{H^{-1/2+s}(\varGamma )}. \end{aligned}$$

$$\square $$

In addition to the single layer operator *V*, we will need to understand localized versions of these operators, i.e., the properties of commutators. For a smooth cut-off function $\eta$, we define the commutators

3.5 $$\begin{aligned} (C_{\eta } \widehat{\phi })(x) {:}{=} (V(\eta \widehat{\phi }) - \eta V(\widehat{\phi }))(x)= & {} \int _{\varGamma }G(x,y)(\eta (x)-\eta (y))\widehat{\phi }(y) ds_y,\end{aligned}$$

3.6 $$\begin{aligned} (C_{\eta }^{\eta } \widehat{\phi })(x) {:}{=} (C_{\eta }(\eta \widehat{\phi }) - \eta C_{\eta }(\widehat{\phi }))(x)= & {} \int _{\varGamma }G(x,y)(\eta (x)-\eta (y))^2\widehat{\phi }(y) ds_y.\quad \end{aligned}$$

Since the singularity of the Green’s function at $x=y$ is smoothed by $\eta (x)-\eta (y)$, we expect that the commutators $C_{\eta }$, $C_{\eta }^{\eta }$ have better mapping properties than the single-layer operator; this is stated in the following lemma.

### Lemma 3.3

Let $\eta \in C^\infty _0({\mathbb {R}}^d)$ be fixed.

(i) Let $s\in (-1/2,1/2)$. The commutator $C_{\eta }$ can be extended in a unique way to a bounded linear operator $C_{\eta }:H^{-1+s}(\varGamma )\rightarrow H^{1+s}(\varGamma )$. The continuity constant depends only on $\Vert \eta \Vert _{W^{1,\infty }({\mathbb {R}}^d)}$, $\varOmega$, and *s*. Furthermore, the operator is skew-symmetric (with respect to the $L^2(\varGamma )$-inner product).
(ii) The commutator $C_{\eta }^{\eta }$ is a symmetric and continuous mapping $C_{\eta }^{\eta }:H^{-1-\alpha _D}(\varGamma ) \rightarrow H^{1+\alpha _D}(\varGamma )$. Here, the continuity constant depends only on $\Vert \eta \Vert _{W^{1,\infty }({\mathbb {R}}^d)}$, $\varOmega$, and the constants appearing in Assumption 2.1.

### Proof:

*Proof of (i):* *1. step:* We show the boundedness for the case $0< s < 1/2$. Let $\widehat{\phi } \in H^{-1+s}(\varGamma )$, and set

3.7 $$\begin{aligned} u{:}{=}{\widetilde{C}}_{\eta }\widehat{\phi }\, {:=}\, {\widetilde{V}}(\eta \widehat{\phi })-\eta {\widetilde{V}}(\widehat{\phi }). \end{aligned}$$

Since the volume potential ${\widetilde{V}} \widehat{\phi }$ is harmonic and in view of the jump relations $[\gamma _0 {\widetilde{V}}\phi ] = 0$, $[\partial _n {\widetilde{V}}\phi ] = -\phi$ satisfied by ${\widetilde{V}}$, cf. [22, Thm. 3.3.1], we have

$$\begin{aligned} \begin{array}{lll} &{}-\varDelta u = 2\nabla \eta \cdot \nabla {\widetilde{V}}\widehat{\phi } + \varDelta \eta {\widetilde{V}}\widehat{\phi } &{} \quad \text {in} \, {\mathbb {R}}^d\backslash \varGamma ,\\ &{}[\gamma _0 u] =0, \quad [\partial _n u] = 0 &{} \quad \text {on}\, \varGamma . \end{array} \end{aligned}$$

We may write $u = {\mathcal {N}}(2\nabla \eta \cdot \nabla {\widetilde{V}}\widehat{\phi } + \varDelta \eta {\widetilde{V}}\widehat{\phi })$ with the Newton potential

3.8 $$\begin{aligned} {\mathcal {N}}f(x){:}{=} \int _{{\mathbb {R}}^d}G(x,y)f(y)dy, \end{aligned}$$

since *u* and ${\mathcal {N}}(2\nabla \eta \cdot \nabla {\widetilde{V}}\widehat{\phi } + \varDelta \eta {\widetilde{V}}\widehat{\phi })$ have the same decay for $\left| x \right| \rightarrow \infty$. The mapping properties of the Newton potential (see, e.g., [22, Thm. 3.1.2]), as well as the mapping properties of ${\widetilde{V}}$ of Lemma 3.2, (i) provide

3.9 $$\begin{aligned} \left\| u \right\| _{H^{3/2+s}(B_{R_{\varOmega }}(0)\backslash \varGamma )}\lesssim & {} \left\| 2\nabla \eta \cdot \nabla {\widetilde{V}}\widehat{\phi } + \varDelta \eta {\widetilde{V}}\widehat{\phi } \right\| _{H^{-1/2+s}(B_{R_{\varOmega }}(0)\backslash \varGamma )} \nonumber \\\lesssim & {} \left\| {\widetilde{V}}\widehat{\phi } \right\| _{H^{1/2+s}(B_{R_{\varOmega }}(0)\backslash \varGamma )}\lesssim \left\| \widehat{\phi } \right\| _{H^{-1+s}(\varGamma )}. \end{aligned}$$

The definition of $C_{\eta }$ and the definition of the norm $\left\| \cdot \right\| _{H^{1+s}(\varGamma )}$ from (1.3) prove the mapping properties of $C_{\eta }$ for $0< s < 1/2$.

The mapping properties of the Newton potential ( see, e.g., [22, Thm. 3.1.2]) also lead to

3.10 $$\begin{aligned} \left\| {\widetilde{C}}_{\eta }\widehat{\phi } \right\| _{H^{1/2+s}(B_{R_{\varOmega }}(0)\backslash \varGamma )}=\left\| u \right\| _{H^{1/2+s}(B_{R_{\varOmega }}(0)\backslash \varGamma )} \lesssim \left\| {\widetilde{V}}\widehat{\phi } \right\| _{H^{-1/2+s}(B_{R_{\varOmega }}(0)\backslash \varGamma )}.\nonumber \\ \end{aligned}$$

*2. step:* Since *V* is symmetric, we have for arbitrary $\widehat{\phi }$, $\psi \in H^{-1/2}(\varGamma )$

$$\begin{aligned} \left\langle C_{\eta }\widehat{\phi },\psi \right\rangle = \left\langle V(\eta \widehat{\phi }) - \eta V(\widehat{\phi }),\psi \right\rangle = \left\langle \widehat{\phi },\eta V(\psi ) - V(\eta \psi ) \right\rangle = - \left\langle \widehat{\phi },C_{\eta }\psi \right\rangle . \end{aligned}$$

With the mapping property $C_{\eta }: H^{-1+s}(\varGamma ) \rightarrow H^{1+s}(\varGamma )$ for $0<s<1/2$, we see that the right-hand side of this equation extends to a bounded linear functional on $H^{-1-s}(\varGamma )$, which proves the mapping properties for the case $-1/2<s<0$.

A similar computation proves the symmetry of the commutator $C_{\eta }^{\eta }$ asserted in (ii).

*3. step:* We have $C_{\eta }: H^{-1+s}(\varGamma )\rightarrow H^{1+s}(\varGamma )$ for $s \in (-1/2,1/2)\backslash \{0\}$. An interpolation argument extends the boundedness to the remaining case $s=0$.

*Proof of (ii):* Let

3.11 $$\begin{aligned} v:=\widetilde{C_{\eta }^{\eta }}\widehat{\phi } := {\widetilde{C}}_{\eta }(\eta \widehat{\phi })-\eta {\widetilde{C}}_{\eta }\widehat{\phi }. \end{aligned}$$

Since

$$\begin{aligned} \varDelta {\widetilde{C}}_{\eta }(\eta \widehat{\phi })-\eta \varDelta {\widetilde{C}}_{\eta } \widehat{\phi } = -2\nabla \eta \cdot \nabla {\widetilde{C}}_{\eta }\widehat{\phi } - \varDelta \eta {\widetilde{C}}_{\eta }\widehat{\phi }-2\left| \nabla \eta \right| ^2{\widetilde{V}}\widehat{\phi }, \end{aligned}$$

the function *v* solves

3.12 $$\begin{aligned} -\varDelta v&= 4\nabla \eta \cdot \nabla {\widetilde{C}}_{\eta }\widehat{\phi } + 2\varDelta \eta {\widetilde{C}}_{\eta }\widehat{\phi } + 2\left| \nabla \eta \right| ^2{\widetilde{V}}\widehat{\phi }&\; \text {in}\, {\mathbb {R}}^d\backslash \varGamma ,\end{aligned}$$

3.13 $$\begin{aligned} {[\gamma _0 v]}&=0, \quad [\partial _n v] = 0&\; \text {on}\, \varGamma . \end{aligned}$$

Again, the function *v* and the Newton potential of the right-hand side of (3.12) have the same decay for $\left| x \right| \rightarrow \infty$, and the mapping properties of the Newton potential as well as the previous estimate (3.10) for ${\widetilde{C}}_{\eta }\widehat{\phi }$ provide

3.14 $$\begin{aligned} \begin{aligned}&\left\| v \right\| _{H^{3/2+\alpha _D}(B_{R_{\varOmega }}(0)\backslash \varGamma )} \lesssim \left\| 4\nabla \eta \cdot \nabla {\widetilde{C}}_{\eta }\widehat{\phi } + 2\varDelta \eta {\widetilde{C}}_{\eta }\widehat{\phi }+ 2\left| \nabla \eta \right| ^2{\widetilde{V}}\widehat{\phi } \right\| _{H^{-1/2+\alpha _D}(B_{R_{\varOmega }}(0)\backslash \varGamma )} \\ {}&\qquad \lesssim \left\| {\widetilde{C}}_{\eta }\widehat{\phi } \right\| _{H^{1/2+\alpha _D}(B_{R_{\varOmega }}(0)\backslash \varGamma )}+ \left\| {\widetilde{V}}\widehat{\phi } \right\| _{H^{-1/2+\alpha _D}(B_{R_{\varOmega }}(0)\backslash \varGamma )} \\&\quad {\mathop {\lesssim }\limits ^{(3.10)}} \left\| {\widetilde{V}}\widehat{\phi } \right\| _{H^{-1/2+\alpha _D}(B_{R_{\varOmega }}(0)\backslash \varGamma )} {\mathop {\lesssim }\limits ^{\alpha _D < 1/2}} \left\| {\widetilde{V}}\widehat{\phi } \right\| _{H^{1/2-\alpha _D}(B_{R_{\varOmega }}(0)\backslash \varGamma )}. \end{aligned} \end{aligned}$$

We apply Lemma 3.1 to ${\widetilde{V}}\widehat{\phi }$. Since , we have that ${\widetilde{V}}\widehat{\phi }$ is smooth on $\partial B_{R_{\varOmega }}(0)$, and we can estimate this term by an arbitrary negative norm of $\widehat{\phi }$ on $\varGamma$ to obtain

$$\begin{aligned}&\left\| {\widetilde{V}}\widehat{\phi } \right\| _{H^{1/2-\alpha _D}(B_{R_{\varOmega }}(0)\backslash \varGamma )} {\mathop {\lesssim }\limits ^{(3.1)}} \left\| \gamma _0 {\widetilde{V}}\widehat{\phi } \right\| _{H^{-\alpha _D}(\varGamma \cup \partial B_{R_{\varOmega }}(0))} \\&\quad \lesssim \left\| V\widehat{\phi } \right\| _{H^{-\alpha _D}(\varGamma )}+ \left\| \widehat{\phi } \right\| _{H^{-1-\alpha _D}(\varGamma )}. \end{aligned}$$

The mapping properties of *V* of Lemma 3.2, (ii) and the symmetry of *V* imply

$$\begin{aligned} \left\| V\widehat{\phi } \right\| _{H^{-\alpha _D}(\varGamma )}= & {} \sup _{w\in H^{\alpha _D}(\varGamma )} \frac{\left\langle V\widehat{\phi },w \right\rangle _{L^2(\varGamma )}}{\left\| w \right\| _{H^{\alpha _D}(\varGamma )}} = \sup _{w\in H^{\alpha _D}(\varGamma )} \frac{\left\langle \widehat{\phi },Vw \right\rangle _{L^2(\varGamma )}}{\left\| w \right\| _{H^{\alpha _D}(\varGamma )}} \\\lesssim & {} \sup _{w\in H^{\alpha _D}(\varGamma )} \frac{\left\| \widehat{\phi } \right\| _{H^{-1-\alpha _D}(\varGamma )}\left\| Vw \right\| _{H^{1+\alpha _D}(\varGamma )}}{\left\| w \right\| _{H^{\alpha _D}(\varGamma )}} \lesssim \left\| \widehat{\phi } \right\| _{H^{-1-\alpha _D}(\varGamma )}. \end{aligned}$$

Inserting this in (3.14) leads to $\left\| v \right\| _{H^{3/2+\alpha _D}(B_{R_{\varOmega }}(0)\backslash \varGamma )} \lesssim \left\| \widehat{\phi } \right\| _{H^{-1-\alpha _D}(\varGamma )},$ which, together with the definition of the $H^{1+\alpha _D}(\varGamma )$-norm in (1.3), proves the lemma. $\square$

The shift theorem for the Neumann problem from Assumption 2.7 implies the following shift theorem.

### Lemma 3.4

Let Assumption 2.7 be valid, and let *u* be the solution of the inhomogeneous problems

$$\begin{aligned} -\varDelta u&= 0 \quad \text{ in } \varOmega , \quad&\gamma _1^\mathrm{int} u = g_N \quad \text{ on } \varGamma ,&\qquad \left\langle u,1 \right\rangle _{L^2(\varOmega )} = 0,\\ -\varDelta u&= 0 \quad \text{ in } B_{R_{\varOmega }}(0)\backslash \overline{\varOmega }, \quad&\gamma _1^\mathrm{ext} u = g_N \quad \text{ on } \varGamma ,&\quad \gamma _0^\mathrm{int} u = g_D \quad \text{ on } \partial B_{R_{\varOmega }}(0), \end{aligned}$$

where $g_N\in H^{-1/2}(\varGamma )$ with $\left\langle g_N,1 \right\rangle _{L^2(\varGamma )} = 0$, and $g_D\in H^{1/2}(\partial B_{R_{\varOmega }}(0))$.

(i) There is a constant $C>0$ depending only on $\varOmega$ and $\alpha _N$ such that
  3.15 $$\begin{aligned} \left\| u \right\| _{H^{1/2-\alpha _N}(B_{R_{\varOmega }}(0)\backslash \varGamma )}\le C \left( \left\| g_N \right\| _{H^{-1-\alpha _N}(\varGamma )} + \left\| g_D \right\| _{H^{-\alpha _N}(\partial B_{R_{\varOmega }}(0))}\right) . \end{aligned}$$
(ii) Let $\varepsilon \in (0,\alpha _N]$. Let $B\subset B' \subset B_{R_{\varOmega }}(0)$ be nested subdomains with  and let $\eta \in C_0^{\infty }({\mathbb {R}}^d)$ satisfy $\eta \equiv 1$ on $B\cap \varGamma$, , and  for $k \in \{0,1,2\}$. Assume $\eta g_N\in H^{\varepsilon }(\varGamma )$. Then
  3.16 $$\begin{aligned} \left\| u \right\| _{H^{3/2+\varepsilon }(B\backslash \varGamma )} \le C\left( \left\| u \right\| _{H^1(B'\backslash \varGamma )} +\left\| \eta g_N \right\| _{H^{\varepsilon }(\varGamma )}\right) . \end{aligned}$$
  Here, the constant $C>0$ depends on $\varOmega , \alpha _N$, and .

### Proof:

*Proof of (i):* For $w \in H^{-1/2+\alpha _N}(B_{R_{\varOmega }}(0)\backslash \varGamma )$ and $\overline{w}:=\frac{1}{\left| \varOmega \right| }\left\langle w,1 \right\rangle _{L^2(\varOmega )}$, let *v* solve

$$\begin{aligned} -\varDelta v&= w-\overline{w} \quad \text{ in } \varOmega , \quad&\gamma _1^\mathrm{int} v = 0 \quad \text{ on } \varGamma ,&\qquad \left\langle v,1 \right\rangle _{L^2(\varOmega )} = 0,\\ -\varDelta v&= w \quad \text{ in } B_{R_{\varOmega }}(0)\backslash \overline{\varOmega }, \quad&\gamma _1^\mathrm{ext} v = 0 \quad \text{ on } \varGamma ,&\quad \gamma _0^\mathrm{int} v = 0 \quad \text{ on } \partial B_{R_{\varOmega }}(0). \end{aligned}$$

Then, with $\left\langle u,1 \right\rangle _{L^2(\varOmega )} = 0$, we have

$$\begin{aligned} \left\| u \right\| _{H^{1/2-\alpha _N}(B_{R_{\varOmega }}(0)\backslash \varGamma )}= & {} \sup _{w \in H^{-1/2+\alpha _N}(B_{R_{\varOmega }}(0)\backslash \varGamma )} \frac{\left\langle u,w \right\rangle _{L^2(B_{R_{\varOmega }}(0)\backslash \varGamma )}}{\left\| w \right\| _{H^{-1/2+\alpha _N}(B_{R_{\varOmega }}(0)\backslash \varGamma )}} \\= & {} \sup _{w \in H^{-1/2+\alpha _N}(B_{R_{\varOmega }}(0)\backslash \varGamma )} \frac{\left\langle u,w-\overline{w} \right\rangle _{L^2(\varOmega )}+\left\langle u,w \right\rangle _{L^2(B_{R_{\varOmega }}(0)\backslash \overline{\varOmega })}}{\left\| w \right\| _{H^{-1/2+\alpha _N}(B_{R_{\varOmega }}(0)\backslash \varGamma )}} \\= & {} \sup _{w \in H^{-1/2+\alpha _N}(B_{R_{\varOmega }}(0)\backslash \varGamma )} \frac{-\left\langle u,\varDelta v \right\rangle _{L^2(B_{R_{\varOmega }}(0)\backslash \varGamma )}}{\left\| w \right\| _{H^{-1/2+\alpha _N}(B_{R_{\varOmega }}(0)\backslash \varGamma )}}. \end{aligned}$$

Integration by parts on $\varOmega$ and $B_{R_{\varOmega }}(0)\backslash \overline{\varOmega }$ and the boundary conditions of *v* lead to

$$\begin{aligned} \left\langle u,\varDelta v \right\rangle _{L^2(B_{R_{\varOmega }}(0)\backslash \varGamma )}= & {} \left\langle \varDelta u,v \right\rangle _{L^2(B_{R_{\varOmega }}(0)\backslash \varGamma )} - \left\langle \partial _n u,[\gamma _0 v] \right\rangle _{L^2(\varGamma )} \\&+\left\langle \gamma _0 u,\partial _n v \right\rangle _{L^2(\partial B_{R_{\varOmega }}(0))} \\= & {} -\left\langle g_N,[\gamma _0 v] \right\rangle _{L^2(\varGamma )} + \left\langle g_D,\partial _n v \right\rangle _{L^2(\partial B_{R_{\varOmega }}(0))}. \end{aligned}$$

The definition of the norm (1.3) implies

$$\begin{aligned} \left\| \gamma _0^\mathrm{int} v \right\| _{H^{1+\alpha _N}(\varGamma )} \lesssim \left\| v \right\| _{H^{3/2+\alpha _N}(B_{R_{\varOmega }}(0)\backslash \varGamma )}, \end{aligned}$$

and the same estimate holds for $\gamma _0^\mathrm{ext} v$. Since $\partial B_{R_{\varOmega }}(0)$ is smooth, we may estimate with the trace inequality

$$\begin{aligned} \left\| \partial _n v \right\| _{H^{\alpha _N}(\partial B_{R_{\varOmega }}(0))} \lesssim \left\| v \right\| _{H^{3/2+\alpha _N}(B_{R_{\varOmega }}(0)\backslash \varGamma )}. \end{aligned}$$

This leads to

$$\begin{aligned}&\left\| u \right\| _{H^{1/2-\alpha _N}(B_{R_{\varOmega }}(0)\backslash \varGamma )} \lesssim \sup _{w \in H^{-1/2+\alpha _N}(B_{R_{\varOmega }}(0)\backslash \varGamma )} \frac{\left| \left\langle g_N,[\gamma _0 v] \right\rangle _{L^2(\varGamma )} - \left\langle g_D,\partial _n v \right\rangle _{L^2(\partial B_{R_{\varOmega }}(0))} \right| }{\left\| w \right\| _{H^{-1/2+\alpha _N}(B_{R_{\varOmega }}(0)\backslash \varGamma )}} \\&\qquad \quad \lesssim \sup _{w\in H^{-1/2+\alpha _N}(B_{R_{\varOmega }}(0) \backslash \varGamma )} \frac{\left\| g_N \right\| _{H^{-1-\alpha _N}(\varGamma )} \left\| [\gamma _0 v] \right\| _{H^{1+\alpha _N}(\varGamma )}+ \left\| g_D \right\| _{H^{-\alpha _N}(\partial B_{R_{\varOmega }}(0))} \left\| \partial _n v \right\| _{H^{\alpha _N}(\partial B_{R_{\varOmega }}(0))}}{\left\| w \right\| _{H^{-1/2+\alpha _N}(B_{R_{\varOmega }}(0)\backslash \varGamma )}}\\&\qquad \quad \lesssim \left( \left\| g_N \right\| _{H^{-1-\alpha _N}(\varGamma )}+\left\| g_D \right\| _{H^{-\alpha _N} (\partial B_{R_{\varOmega }}(0))}\right) \sup _{w \in H^{-1/2+\alpha _N}(B_{R_{\varOmega }}(0)\backslash \varGamma )} \frac{\left\| v \right\| _{H^{3/2+\alpha _N}(B_{R_{\varOmega }}(0)\backslash \varGamma )}}{\left\| w \right\| _{H^{-1/2+\alpha _N}(B_{R_{\varOmega }}(0)\backslash \varGamma )}}\\&\qquad \quad {\mathop {\lesssim }\limits ^{\text {Ass.}~2.7}} \left\| g_N \right\| _{H^{-1-\alpha _N}(\varGamma )}+\left\| g_D \right\| _{H^{-\alpha _N}(\partial B_{R_{\varOmega }}(0))}. \end{aligned}$$

*Proof of (ii):* Since $\eta \equiv 0$ on $\partial B_{R_{\varOmega }}(0)$, the function ${\widetilde{u}}{:}{=}\eta u$ satisfies

$$\begin{aligned} -\varDelta {\widetilde{u}}&= -2\nabla \eta \cdot \nabla u - (\varDelta \eta ) u&\; \text {in}\; B_{R_{\varOmega }}(0)\backslash \varGamma , \\ \gamma _1^\mathrm{int} {\widetilde{u}}&= (\partial _n \eta ) \gamma _0^\mathrm{int}u + \eta g_N&\; \text {on}\; \varGamma ,\\ \gamma _1^\mathrm{ext} {\widetilde{u}}&= (\partial _n \eta ) \gamma _0^\mathrm{ext}u + \eta g_N&\; \text {on}\; \varGamma , \\ \gamma _0^\mathrm{int} {\widetilde{u}}&= 0&\; \text {on}\; \partial B_{R_{\varOmega }}(0). \end{aligned}$$

The shift theorem from Assumption 2.7 and the trace inequality $\left\| (\partial _n \eta ) \gamma _0^\mathrm{int}u \right\| _{H^{1/2}(\varGamma )} \lesssim \left\| u \right\| _{H^1(B'\backslash \varGamma )}$ provide

$$\begin{aligned}&\left\| u \right\| _{H^{3/2+\varepsilon }(B\backslash \varGamma )} \le \left\| {\widetilde{u}} \right\| _{H^{3/2+\varepsilon }(B_{R_{\varOmega }}(0)\backslash \varGamma )} \\&\quad \lesssim \left\| 2\nabla \eta \cdot \nabla u + (\varDelta \eta ) u \right\| _{L^2(B_{R_{\varOmega }}(0)\backslash \varGamma )} + \left\| (\partial _n\eta ) \gamma _0^\mathrm{int} u \right\| _{H^{\varepsilon }(\varGamma )} \\&\qquad + \left\| (\partial _n\eta ) \gamma _0^\mathrm{ext} u \right\| _{H^{\varepsilon }(\varGamma )} +\quad \left\| \eta g_N \right\| _{H^{\varepsilon }(\varGamma )} \\&\quad \lesssim \left\| u \right\| _{H^1(B'\backslash \varGamma )} + \left\| \eta g_N \right\| _{H^{\varepsilon }(\varGamma )}, \end{aligned}$$

which proves the second statement. $\square$

The following lemma collects mapping properties of the double-layer operator *K* and the hyper-singular operator *W* that exploit the present setting of piecewise smooth geometries:

### Lemma 3.5

Define the *double layer potential* ${\widetilde{K}}$ by

3.17 $$\begin{aligned} {\widetilde{K}}{\varphi }(x) {:}{=} \int _{\varGamma }\partial _{n_y}G(x,y){\varphi }(y) ds_y, \qquad x \in {\mathbb {R}}^d {\setminus }\varGamma . \end{aligned}$$

(i) The double layer potential ${\widetilde{K}}$ is a bounded linear operator from $H^{1/2+s}(\varGamma )$ to $H^{1+s}(B_{R_\varOmega }(0)\backslash \varGamma )$ for $-1/2\le s \le 1/2+\alpha _N$.
(ii) The double layer operator *K* is a bounded linear operator from $H^{1/2+s}(\varGamma )$ to $H^{1/2+s}(\varGamma )$ for $-1/2 \le s \le 1/2+\alpha _N$.
(iii) The hyper singular operator *W* is a bounded linear operator from $H^{1/2+s}(\varGamma )$ to $H^{-1/2+s}(\varGamma )$ for $-1/2-\alpha _N \le s \le 1/2+\alpha _N$.

### Proof:

*Proof of (i):* With the mapping properties of the single layer potential ${\widetilde{V}}\in L(H^{-1/2+s}(\varGamma ),H^{1+s}(B_{R_\varOmega }(0)\backslash \varGamma ))$ from Lemma 3.2, the mapping properties of the solution operator of the Dirichlet problem from Assumption 2.1 ($T:H^{1/2+s}(\varGamma )\rightarrow H^{1+s}(B_{R_\varOmega }(0)\backslash \varGamma )$), and the assumption $\alpha _N \le \alpha _D$, the mapping properties of ${\widetilde{K}}$ follow from Green’s formula by expressing ${\widetilde{K}}$ in terms of ${\widetilde{V}}$, *T*, and the Newton potential ${\mathcal {N}}$. For details, we refer to [22, Thm. 3.1.16], where the case $s \in (-1/2,1/2)$ is shown.

*Proof of (ii):* The case $-1/2 \le s \le 1/2$ is taken from [22, Thm. 3.1.16]. For $s \in (1/2,1/2+\alpha _N]$ the result follows from part (i), the definition of the norm $\Vert \cdot \Vert _{H^{s+1/2}(\varGamma )}$ given in (1.3), and $K = \gamma _0^\mathrm{int}{\widetilde{K}}+\frac{1}{2}\mathrm {Id}$.

*Proof of (iii):* The case $-1/2 \le s \le 1/2$ is taken from [22, Thm. 3.1.16]. Since $W = -\partial _n {\widetilde{K}}$, we get with a facewise trace estimate as in the proof of Lemma 3.1, estimate (3.3), that

$$\begin{aligned} \left\| W\varphi \right\| _{H^{-1/2+s}(\varGamma )}=\left\| \partial _n {\widetilde{K}}\varphi \right\| _{H^{-1/2+s}(\varGamma )} \lesssim \left\| {\widetilde{K}}\varphi \right\| _{H^{1+s}(\varOmega )} \lesssim \left\| \varphi \right\| _{H^{1/2+s}(\varGamma )}, \end{aligned}$$

which finishes the proof for the case $s \in (1/2,1/2+\alpha _N]$. With the symmetry of *W*, the case $s \in [-1/2-\alpha _N,-1/2)$ follows. $\square$

For a smooth function $\eta$, we define the commutators

3.18 $$\begin{aligned} {\mathcal {C}}_{\eta } \widehat{\varphi }&{:}{=}&W(\eta \widehat{\varphi }) - \eta W \widehat{\varphi }, \end{aligned}$$

3.19 $$\begin{aligned} {\mathcal {C}}_{\eta }^{\eta }(\widehat{\varphi })&{:}{=}&{\mathcal {C}}_{\eta }(\eta \widehat{\varphi }) - \eta {\mathcal {C}}_{\eta }(\widehat{\varphi }) = W(\eta ^2\widehat{\varphi })-2\eta W(\eta \widehat{\varphi })+\eta ^2 W (\widehat{\varphi }). \end{aligned}$$

By the mapping properties of *W*, both operators map $H^{1/2}(\varGamma ) \rightarrow H^{-1/2}(\varGamma )$. However, ${\mathcal {C}}_{\eta }$ is in fact an operator of order 0 and ${\mathcal {C}}_{\eta }^{\eta }$ is an operator of positive order:

### Lemma 3.6

Fix $$\eta \in C_0^\infty ({\mathbb {R}}^d)$$.

(i) Let $s \in (-1/2,1/2)$. Then, the commutator ${\mathcal {C}}_\eta$ can be extended in a unique way to a bounded linear operator $H^s(\varGamma ) \rightarrow H^s(\varGamma )$, which satisfies
  3.20 $$\begin{aligned} \Vert {\mathcal C}_\eta \varphi \Vert _{H^{s}(\varGamma )} \le C \Vert \varphi \Vert _{H^s(\varGamma )} \qquad \forall \varphi \in H^s(\varGamma ). \end{aligned}$$
  The constant *C* depends only on $\Vert \eta \Vert _{W^{1,\infty }({\mathbb {R}}^d)}$, $\varOmega$, and *s*. Furthermore, the operator is skew-symmetric (with respect to the extended $L^2$-inner product).
(ii) The commutator ${\mathcal {C}}_{\eta }^{\eta }$ is a symmetric and continuous mapping ${\mathcal {C}}_{\eta }^{\eta }:H^{-\alpha _N}(\varGamma ) \rightarrow H^{\alpha _N}(\varGamma )$. The continuity constant depends only on $\Vert \eta \Vert _{W^{1,\infty }({\mathbb {R}}^d)}$, $\varOmega$, and the constants appearing in Assumption 2.7.

### Proof:

*Proof of (i):* *1. step:* We show (3.20) for the range $0< s < 1/2$. For $\varphi \in H^{1/2}(\varGamma )$, consider the operator

3.21 $$\begin{aligned} \widetilde{{\mathcal {C}}}_{\eta }\varphi {:}{=} {\widetilde{K}}(\eta \varphi )-\eta {\widetilde{K}}(\varphi ) - {\widetilde{V}}((\partial _n \eta ) \varphi ) \end{aligned}$$

with the single layer potential ${\widetilde{V}}$ and the double layer potential ${\widetilde{K}}$ from (3.17). Using the jump conditions $[\gamma _0 {\widetilde{V}}\phi ] = 0$, $[\partial _n {\widetilde{V}}\phi ] = -\phi$ for $\widetilde{V}$ and additionally the jump relations $[\gamma _0 {\widetilde{K}}\phi ] = \phi$, $[\partial _n {\widetilde{K}}\phi ] = 0$ satisfied by $\widetilde{K}$ from [22, Thm. 3.3.1], we observe that the function $u:= \widetilde{{\mathcal {C}}}_{\eta }\varphi$ solves

$$\begin{aligned} -\varDelta u&= 2\nabla \eta \cdot \nabla {\widetilde{K}}\varphi + (\varDelta \eta ) {\widetilde{K}}\varphi&\; \text {in} \; {\mathbb {R}}^d\backslash \varGamma , \\ [\gamma _0 u]&=0, \quad [\partial _n u] = -\partial _n \eta [\gamma _0 {\widetilde{K}}\varphi ] - [\partial _n {\widetilde{V}}(\partial _n \eta \varphi )] = 0&\; \text {on} \; \varGamma . \end{aligned}$$

The decay of *u* - the dominant part is the single-layer potential - and the Newton potential ${\mathcal {N}}(2\nabla \eta \cdot \nabla {\widetilde{K}}\varphi + (\varDelta \eta ) {\widetilde{K}}\varphi )$ for $\left| x \right| \rightarrow \infty$ are the same, which allows us to write $u={\mathcal {N}}(2\nabla \eta \cdot \nabla {\widetilde{K}}\varphi + (\varDelta \eta ) {\widetilde{K}}\varphi )$. With the mapping properties of the Newton potential and the standard mapping properties of ${\widetilde{K}}$ from [22, Thm. 3.1.16], we get

3.22 $$\begin{aligned} \left\| u \right\| _{H^{3/2+s}(B_{R_{\varOmega }}(0)\backslash \varGamma )}&\lesssim \left\| \nabla \eta \cdot \nabla {\widetilde{K}}\varphi + \varDelta \eta {\widetilde{K}}\varphi \right\| _{H^{-1/2+s}(B_{R_{\varOmega }}(0) \backslash \varGamma )}\nonumber \\&\lesssim \left\| {\widetilde{K}}\varphi \right\| _{H^{1/2+s}(B_{R_{\varOmega }}(0) \backslash \varGamma )} \lesssim \left\| \varphi \right\| _{H^{s}(\varGamma )}. \end{aligned}$$

The trace estimate applied facewise as in the proof of Lemma 3.1, and estimates (3.3), (3.22) lead to

3.23 $$\begin{aligned} \left\| \partial _n \widetilde{\mathcal C}_\eta \varphi \right\| _{H^s(\varGamma )} = \left\| \partial _n u \right\| _{H^{s}(\varGamma )} \lesssim \left\| u \right\| _{H^{3/2+s}(B_{R_{\varOmega }}(0)\backslash \varGamma )} \lesssim \left\| \varphi \right\| _{H^{s}(\varGamma )}. \end{aligned}$$

Similarly, we obtain with Lemma 3.2, (i)

3.24 $$\begin{aligned} \Vert \partial _n \widetilde{V} (\eta \varphi )\Vert _{H^{s}(\varGamma )} \lesssim \Vert \widetilde{V} (\eta \varphi )\Vert _{H^{3/2+s}(B_{R_{\varOmega }}(0)\backslash \varGamma )} \lesssim \Vert \eta \varphi \Vert _{H^{s}(\varGamma )}. \end{aligned}$$

Next, we identify $\partial _n \widetilde{\mathcal C}_\eta$. With $W = -\partial _n \widetilde{K}$, , and , we compute

$$\begin{aligned} \partial _n \widetilde{\mathcal C}_\eta \varphi = \eta W \varphi - W (\eta \varphi ) - K'((\partial _n \eta ) \varphi ) - (\partial _n \eta ) K \varphi . \end{aligned}$$

Recalling the mapping properties $K',K:H^s(\varGamma )\rightarrow H^s(\varGamma )$ and the relation , we get with the aid of (3.23), (3.24)

3.25 $$\begin{aligned} \Vert W(\eta \varphi ) - \eta W \varphi \Vert _{H^s(\varGamma )} \lesssim \Vert \varphi \Vert _{H^s(\varGamma )}. \end{aligned}$$

*2. step:* Since $H^{1/2}(\varGamma )$ is dense in $H^s(\varGamma )$, $s \in (0,1/2)$, the operator ${\mathcal C}_\eta$ can be extended (in a unique way) to a bounded linear operator $H^s(\varGamma ) \rightarrow H^s(\varGamma )$.

*3. step:* The operator ${\mathcal C}_\eta$ is skew-symmetric: The operator *W* maps $H^{1/2}(\varGamma ) \rightarrow H^{-1/2}(\varGamma )$ and is symmetric. The skew-symmetry of ${\mathcal C}_\eta$ then follows from a direct calculation.

*4. step:* The skew-symmetry of ${\mathcal C}_\eta$ allows us to extend (in a unique way) the operator as an operator $H^{-s}(\varGamma ) \rightarrow H^{-s}(\varGamma )$ for $0<s<1/2$ by the following argument: For $\varphi$, $\psi \in H^{1/2}(\varGamma )$ we compute

3.26 $$\begin{aligned} \langle {\mathcal C}_\eta \varphi , \psi \rangle = - \langle \varphi , {\mathcal C}_\eta \psi \rangle . \end{aligned}$$

Since ${\mathcal C}_\eta : H^{s}(\varGamma ) \rightarrow H^{s}(\varGamma )$ for $0< s < 1/2$, we see that $\varphi \mapsto \langle \varphi , {\mathcal C}_\eta \psi \rangle$ of the right-hand side of (3.26) extends to a bounded linear functional on $H^{-s}(\varGamma )$. Hence, ${\mathcal C}_\eta : H^{-s}(\varGamma ) \rightarrow H^{-s}(\varGamma )$ for $0< s < 1/2$.

*5. step:* We have ${\mathcal C}_\eta : H^s(\varGamma ) \rightarrow H^s(\varGamma )$ for $s \in (-1/2,1/2){\setminus } \{0\}$. An interpolation argument allows us to extend the boundedness to the remaining case $s = 0$.

*Proof of (ii):* Let $\widehat{\varphi } \in H^{-\alpha _N}(\varGamma )$. The argument leading to the first inequality in (3.22), i.e., the mapping properties of $\mathcal {N}$, also shows

3.27 $$\begin{aligned} \Vert \widetilde{\mathcal C}_\eta \widehat{\varphi }\Vert _{H^{1/2+\alpha _N}(B_{R_\varOmega }(0){\setminus }\varGamma )} \lesssim \Vert \widetilde{K} \widehat{\varphi }\Vert _{H^{-1/2+\alpha _N}(B_{R_\varOmega }(0){\setminus }\varGamma )}. \end{aligned}$$

Since

$$\begin{aligned} \varDelta \widetilde{{\mathcal {C}}}_{\eta }(\eta \widehat{\varphi })-\eta \varDelta \widetilde{{\mathcal {C}}}_{\eta } \widehat{\varphi } = -2\nabla \eta \cdot \nabla \widetilde{{\mathcal {C}}}_{\eta }\widehat{\varphi } - \varDelta \eta \widetilde{{\mathcal {C}}}_{\eta }\widehat{\varphi }-2\left| \nabla \eta \right| ^2{\widetilde{K}}\widehat{\varphi } -\varDelta (\eta {\widetilde{V}}(\partial _n \eta \widehat{\varphi })), \end{aligned}$$

the function $v:=\widetilde{{\mathcal {C}}}_{\eta }^{\eta }\widehat{\varphi } {:}{=} \widetilde{{\mathcal {C}}}_{\eta }(\eta \widehat{\varphi })-\eta \widetilde{{\mathcal {C}}}_{\eta }\widehat{\varphi }$ solves

$$\begin{aligned} -\varDelta v&= 4\nabla \eta \cdot \nabla \widetilde{{\mathcal {C}}}_{\eta }\widehat{\varphi } + 2\varDelta \eta \widetilde{{\mathcal {C}}}_{\eta }\widehat{\varphi } + 2\left| \nabla \eta \right| ^2{\widetilde{K}}\widehat{\varphi } + \varDelta (\eta {\widetilde{V}}(\partial _n \eta \widehat{\varphi }))&\; \text {in}\, {\mathbb {R}}^d\backslash \varGamma ,\\ [\gamma _0 v]&=0, \quad [\partial _n v] = 0&\; \text {on}\, \varGamma . \end{aligned}$$

Again, the decay of *v* and the Newton potential applied to the right-hand side of the equation are the same. Hence, the mapping properties of the Newton potential provide

3.28 $$\begin{aligned}&\left\| v \right\| _{H^{3/2+\alpha _N}(B_{R_{\varOmega }}(0)\backslash \varGamma )}\nonumber \\&\quad \lesssim \left\| 4\nabla \eta \cdot \nabla \widetilde{{\mathcal {C}}}_{\eta }\widehat{\varphi } + 2\varDelta \eta \widetilde{{\mathcal {C}}}_{\eta }\widehat{\varphi }+ 2\left| \nabla \eta \right| ^2{\widetilde{K}}\widehat{\varphi }+\varDelta (\eta {\widetilde{V}}(\partial _n \eta \widehat{\varphi })) \right\| _{H^{-1/2+\alpha _N}(B_{R_{\varOmega }}(0)\backslash \varGamma )} \nonumber \\&\quad \lesssim \left\| \widetilde{{\mathcal {C}}}_{\eta }\widehat{\varphi } \right\| _{H^{1/2 +\alpha _N}(B_{R_{\varOmega }}(0)\backslash \varGamma )}+ \left\| {\widetilde{K}}\widehat{\varphi } \right\| _{H^{-1/2+\alpha _N} (B_{R_{\varOmega }}(0)\backslash \varGamma )} \nonumber \\&\qquad +\left\| {\widetilde{V}}(\partial _n \eta \widehat{\varphi }) \right\| _{H^{1/2 +\alpha _N}(B_{R_{\varOmega }}(0)\backslash \varGamma )}\nonumber \\&\quad {\mathop {\lesssim }\limits ^{(3.27)}} \left\| {\widetilde{K}}\widehat{\varphi } \right\| _{H^{-1/2+\alpha _N}(B_{R_{\varOmega }} (0)\backslash \varGamma )} + \left\| \widehat{\varphi } \right\| _{H^{-1+\alpha _N}(\varGamma )} \nonumber \\&\quad {\mathop {\lesssim }\limits ^{\alpha _N < 1/2}} \left\| {\widetilde{K}}\widehat{\varphi } \right\| _{H^{1/2-\alpha _N}(B_{R_{\varOmega }} (0)\backslash \varGamma )}+ \left\| \widehat{\varphi } \right\| _{H^{-1+\alpha _N}(\varGamma )}. \end{aligned}$$

We apply Lemma 3.4 to ${\widetilde{K}}\widehat{\varphi }-\overline{{\widetilde{K}}\widehat{\varphi }},$ with $\overline{{\widetilde{K}}\widehat{\varphi }} {:}{=} \frac{1}{\left| \varOmega \right| }\left\langle {\widetilde{K}}\widehat{\varphi },1 \right\rangle _{L^2(\varOmega )}$. Since we assumed , we have that ${\widetilde{K}}\widehat{\varphi }$ is smooth on $\partial B_{R_{\varOmega }}(0)$, and we can estimate this term by an arbitrary negative norm of $\widehat{\varphi }$ on $\varGamma$ to obtainThe mean value $\overline{\widetilde{K}\widehat{\varphi }}$ can be estimated with $r^2 = \left| x \right| ^2$, the observation $\varDelta r^2 = 2d$, and integration by parts by

3.29 $$\begin{aligned} \left| \overline{{\widetilde{K}}\widehat{\varphi }} \right|&\lesssim \left| \left\langle {\widetilde{K}}\widehat{\varphi },\varDelta r^2 \right\rangle _{L^2(\varOmega )} \right| \lesssim \left| \left\langle \gamma _0^\mathrm{int}{\widetilde{K}}\widehat{\varphi },\partial _n r^2 \right\rangle _{L^2(\varGamma )} \right| + \left| \left\langle \gamma _1^\mathrm{int}{\widetilde{K}} \widehat{\varphi }, r^2 \right\rangle _{L^2(\varGamma )} \right| \nonumber \\ \nonumber&\lesssim \left| \left\langle \widehat{\varphi },(K'-1/2)\partial _n r^2 \right\rangle _{L^2(\varGamma )} \right| + \left| \left\langle W\widehat{\varphi }, r^2 \right\rangle _{L^2(\varGamma )} \right| \nonumber \\&\lesssim \left\| \widehat{\varphi } \right\| _{H^{-\alpha _N}(\varGamma )}+ \left\| W\widehat{\varphi } \right\| _{H^{-1-\alpha _N}(\varGamma )}, \end{aligned}$$

where the last step follows since $K'$ is a bounded operator mapping $H^{\alpha _N}(\varGamma )\rightarrow H^{\alpha _N}(\varGamma )$ by Lemma 3.2. Using the mapping properties of *W* of Lemma 3.5, (iii) and inserting (3.29) in (3.28) leads together with a facewise trace estimate to

$$\begin{aligned} \left\| \partial _n v \right\| _{H^{\alpha _N}(\varGamma )}\lesssim \left\| v \right\| _{H^{3/2+\alpha _N}(B_{R_{\varOmega }}(0)\backslash \varGamma )} \lesssim \left\| \widehat{\varphi } \right\| _{H^{-\alpha _N}(\varGamma )}. \end{aligned}$$

The computation

$$\begin{aligned} - {\mathcal {C}}^{\eta }_{\eta }\widehat{\varphi } = \partial _n \widetilde{{\mathcal {C}}}^{\eta }_{\eta }\widehat{\varphi } +K'((\partial _n \eta ) \eta \widehat{\varphi })-\eta K'((\partial _n\eta )\widehat{\varphi })+ 2(\partial _n \eta ) \gamma _0 \widetilde{{\mathcal {C}}}_{\eta }(\widehat{\varphi })+ (\partial _n \eta )V((\partial _n \eta )\widehat{\varphi }), \end{aligned}$$

the mapping properties of *V* and the commutator of $K'$ (as normal trace of the commutator $\widetilde{{C}}_{\eta }$ from Lemma 3.3, cf. (3.9)) prove the lemma. $\square$

## Proof of main results

With the consequences of the shift theorems from the previous section, we can prove our main results, the local error estimates for Symm’s integral equation and the hyper-singular integral equation.

### Symm’s integral equation (proof of Theorem 2.3)

The main tools in our proofs are the Galerkin orthogonality

4.1 $$\begin{aligned} \left\langle V(\phi -\phi _h),\psi _h \right\rangle = 0 \quad \forall \psi _h \in S^{0,0}(\mathcal {T}_h) \end{aligned}$$

and a Caccioppoli-type estimate for discrete harmonic functions that satisfy the orthogonality

4.2 $$\begin{aligned} \left\langle \gamma _0 v,\psi _h \right\rangle =0 \quad \forall \psi _h \in S^{0,0}(\mathcal {T}_h), \text { supp }\psi _h \subset D\cap \varGamma . \end{aligned}$$

More precisely, we employ the space of *discrete harmonic functions* on an open set $D\subset {\mathbb {R}}^d$ defined by

4.3 $$\begin{aligned} {\mathcal {H}}_{h}(D)&{:}{=}\{v\in H^1(D\backslash \varGamma ) :v \text { is harmonic on}\; D\backslash \varGamma ,\nonumber \\&\quad \quad \exists {\widetilde{v}} \in S^{0,0}({{\mathcal {T}}}_h) \;\text{ s.t. } \; [\partial _{n}v]|_{D \cap \varGamma } = {\widetilde{v}}|_{D \cap \varGamma }, \; v \text { satisfies}\; (4.2)\}. \end{aligned}$$

#### Proposition 4.1

[11, Lemma 3.9] For discrete harmonic functions $u \in {\mathcal H}_{h}(B')$, the interior regularity estimate

4.4 $$\begin{aligned} \left\| \nabla u \right\| _{L^2(B)} \lesssim \frac{h}{{\widehat{d}}}\left\| \nabla u \right\| _{L^2(B')} + \frac{1}{{\widehat{d}}}\left\| u \right\| _{L^2(B')} \end{aligned}$$

holds, where *B* and $B'$ are nested boxes and  satisfies $8h\le {\widehat{d}}$. The hidden constant depends only on $\varOmega , d$, and the $\gamma$-shape regularity of $\mathcal {T}_h$.

As a consequence of this interior regularity estimate and Lemma 3.1, we get an estimate for the jump of the normal derivative of a discrete harmonic single-layer potential.

#### Lemma 4.2

Let Assumption 2.1 hold and $B \subset B' \subset B_{R_{\varOmega }}(0)$ be nested boxes with . Let *h* be sufficiently small so that the assumption of Proposition 4.1 holds. Let $u{:}{=}{\widetilde{V}}\zeta _h$ with $\zeta _h \in S^{0,0}(\mathcal {T}_h)$ and assume $u \in {\mathcal H}_h(B')$. Let ${\widehat{\varGamma }}\subset B\cap \varGamma$ and $\eta \in C^{\infty }_0({\mathbb {R}}^d)$ be an arbitrary cut-off function satisfying $\eta \equiv 1$ on $B'\cap \varGamma$. Then,

4.5 $$\begin{aligned} \left\| [\partial _n u] \right\| _{L^2({\widehat{\varGamma }})} \le C \left( h^{\alpha _D/(1+2\alpha _D)}\left\| \eta \zeta _h \right\| _{L^2(\varGamma )} + h^{-1}\left\| \eta V\zeta _h \right\| _{H^{-\alpha _D}(\varGamma )}+ \left\| \zeta _h \right\| _{H^{-1/2}(\varGamma )}\right) .\nonumber \\ \end{aligned}$$

The constant $C>0$ depends only on $\varOmega ,d,{\widehat{d}}$, the $\gamma$-shape regularity of $\mathcal {T}_h$, $\Vert \eta \Vert _{W^{1,\infty }({\mathbb {R}}^d)}$, and the constants appearing in Assumption 2.1.

#### Proof:

We split the function $u = u_{\text {far}} + u_{\text {near}}$, where the near field $u_{\text {near}}$ and the far field $u_{\text {far}}$ solve the Dirichlet problems

$$\begin{aligned} -\varDelta u_{\text {near}}&= 0 \quad \text { in} \; B_{R_{\varOmega }}(0)\backslash \varGamma ,&\quad \gamma _0 u_{\text { near}}&= \eta V\zeta _h \quad \quad \;\; \text {on}\, \varGamma \cup \partial B_{R_{\varOmega }}(0), \\ -\varDelta u_{\text {far}}&= 0 \quad \text { in} \; B_{R_{\varOmega }}(0)\backslash \varGamma ,&\quad \gamma _0u_{\text {far}}&= (1-\eta ) V\zeta _h \quad \text {on}\, \varGamma \cup \partial B_{R_{\varOmega }}(0). \end{aligned}$$

We first consider $\gamma _1^\mathrm{int} u_\mathrm{near}$ - the case $\gamma _1^\mathrm{ext} u_\mathrm{near}$ is treated analogously.

Let ${\widehat{\eta }}$ be another cut-off function satisfying ${\widehat{\eta }} \equiv 1$ on ${\widehat{\varGamma }}$ and . The multiplicative trace inequality, see, e.g., [16, Thm. A.2], implies for any $\varepsilon \le 1/2$ that

4.6 $$\begin{aligned} \left\| \gamma _1^\mathrm{int} u_{\text {near}} \right\| _{L^2({\widehat{\varGamma }})}\lesssim & {} \left\| \gamma _1^\mathrm{int}({\widehat{\eta }} u_{\text {near}}) \right\| _{L^2(B\cap \varGamma )} \nonumber \\\lesssim & {} \left\| \nabla ({\widehat{\eta }} u_{\text {near}}) \right\| _{L^2(\varOmega )}^{2\varepsilon /(1+2\varepsilon )} \left\| \nabla ({\widehat{\eta }} u_{\text {near}}) \right\| _{H^{1/2+\varepsilon } (\varOmega )}^{1/(1+2\varepsilon )}\nonumber \\\lesssim & {} \left\| \nabla (\widehat{\eta }u_{\text {near}}) \right\| _{L^2(B)}^{2\varepsilon /(1+2\varepsilon )} \left\| {\widehat{\eta }}u_{\text {near}} \right\| _{H^{3/2 +\varepsilon }(B)}^{1/(1+2\varepsilon )}. \end{aligned}$$

Since $u_{\text {near}} \in {\mathcal H}_h(B')$, we use the interior regularity estimate (4.2) for the first term on the right-hand side of (4.6), and the second term of (4.6) can be estimated using (3.2). In total, we get for $\varepsilon \le \alpha _D<1/2$ that

4.7 $$\begin{aligned}&\left\| \nabla ({\widehat{\eta }}u_{\text {near}}) \right\| _{L^2(B)}^{2\varepsilon / (1+2\varepsilon )} \left\| {\widehat{\eta }}u_{\text {near}} \right\| _{H^{3/2+\varepsilon }(B)}^{1/ (1+2\varepsilon )}\nonumber \\&\quad \lesssim \left( h\left\| \nabla u_{\text {near}} \right\| _{L^2(B')} + \left\| u_{\text {near}} \right\| _{L^2(B')}\right) ^{2\varepsilon / (1+2\varepsilon )} \nonumber \\&\qquad \cdot \left( \left\| u_{\text {near}} \right\| _{H^1(B')}+\left\| \eta V\zeta _h \right\| _{H^{1+\varepsilon }(\varGamma )} \right) ^{1/(1+2\varepsilon )} \nonumber \\&\quad \lesssim h^{2\varepsilon /(1+2\varepsilon )}\left\| u_{\text {near}} \right\| _{H^1(B')} + \left\| u_{\text {near}} \right\| _{L^2(B')}^{2\varepsilon /(1+2\varepsilon )} \left\| u_{\text {near}} \right\| _{H^1(B')}^{1/(1+2\varepsilon )} \nonumber \\&\qquad + \left\| u_{\text {near}} \right\| _{L^2(B')}^{2\varepsilon /(1+2\varepsilon )} \left\| \eta V\zeta _h \right\| _{H^{1+\varepsilon }(\varGamma )}^{1/(1+2\varepsilon )} + h^{2\varepsilon /(1+2\varepsilon )}\left\| \nabla u_{\text {near}} \right\| _{L^2(B')}^{2\varepsilon /(1+2\varepsilon )} \left\| \eta V\zeta _h \right\| _{H^{1+\varepsilon }(\varGamma )}^{1/(1+2\varepsilon )} \nonumber \\&\quad =: T_1 + T_2 + T_3 + T_4. \end{aligned}$$

Let $\mathcal {I}_h:C(\varGamma ) \rightarrow S^{1,1}({{\mathcal {T}}}_h)$ be the nodal interpolation operator. The mapping properties of *V* from Lemma 3.2, (ii), the commutator $C_{\eta }$ from (3.5) as well as an inverse inequality, see, e.g., [13, Thm. 3.2, Thm. 3.6], lead to

4.8 $$\begin{aligned} \left\| \eta V\zeta _h \right\| _{H^{1+\varepsilon }(\varGamma )}\lesssim & {} \left\| V(\eta \zeta _h) \right\| _{H^{1+\varepsilon }(\varGamma )} + \left\| C_{\eta } \zeta _h \right\| _{H^{1+\varepsilon }(\varGamma )} \lesssim \left\| \eta \zeta _h \right\| _{H^{\varepsilon }(\varGamma )} + \left\| \zeta _h \right\| _{H^{-1+\varepsilon }(\varGamma )} \nonumber \\\lesssim & {} \left\| \mathcal {I}_h(\eta ) \zeta _h \right\| _{H^{\varepsilon }(\varGamma )} + \left\| (\eta - \mathcal {I}_h\eta )\zeta _h \right\| _{H^{\varepsilon }(\varGamma )} +\left\| \zeta _h \right\| _{H^{-1+\varepsilon }(\varGamma )} \nonumber \\\lesssim & {} h^{-\varepsilon }\left\| \mathcal {I}_h(\eta ) \zeta _h \right\| _{L^{2}(\varGamma )} + h\left\| \zeta _h \right\| _{H^{\varepsilon }(\varGamma )} + h^{1-\varepsilon } \left\| \zeta _h \right\| _{L^2(\varGamma )} +\left\| \zeta _h \right\| _{H^{-1+\varepsilon }(\varGamma )} \nonumber \\\lesssim & {} h^{-\varepsilon }\left( \left\| (\eta - \mathcal {I}_h \eta ) \zeta _h \right\| _{L^{2}(\varGamma )} + \left\| \eta \zeta _h \right\| _{L^{2}(\varGamma )}\right) +\left\| \zeta _h \right\| _{H^{-1+\varepsilon }(\varGamma )}\nonumber \\\lesssim & {} h^{-\varepsilon }\left( \left\| \eta \zeta _h \right\| _{L^2(\varGamma )} + \left\| \zeta _h \right\| _{H^{-1}(\varGamma )} \right) . \end{aligned}$$

With the classical *a priori* estimate for the inhomogeneous Dirichlet problem in the $H^1$-norm, the commutator $C_{\eta }$, and Lemma 3.3, we estimate

4.9 $$\begin{aligned} T_1&= h^{2\varepsilon /(1+\varepsilon )} \left\| u_{\text {near}} \right\| _{H^1(B')} \lesssim h^{2\varepsilon /(1+\varepsilon )} \left\| \eta V\zeta _h \right\| _{H^{1/2}(\varGamma )} \nonumber \\&\lesssim h^{2\varepsilon /(1+\varepsilon )} \left( \left\| V(\eta \zeta _h) \right\| _{H^{1/2}(\varGamma )} +\left\| C_{\eta }\zeta _h \right\| _{H^{1/2}(\varGamma )} \right) \nonumber \\&\lesssim h^{2\varepsilon /(1+\varepsilon )}\left( \left\| \eta \zeta _h \right\| _{L^{2}(\varGamma )} + \left\| \zeta _h \right\| _{H^{-1-\alpha _D}(\varGamma )}\right) , \end{aligned}$$

4.10 $$\begin{aligned} T_4&= h^{2\varepsilon /(1+2\varepsilon )}\left\| \nabla u_{\text {near}} \right\| _{L^2(B')}^{2\varepsilon /(1+2\varepsilon )} \left\| \eta V\zeta _h \right\| _{H^{1+\varepsilon }(\varGamma )}^{1/(1+2\varepsilon )}\nonumber \\&{\mathop {\lesssim }\limits ^{(4.8)}} h^{2\varepsilon /(1+2\varepsilon )} \left\| u_{\text {near}} \right\| _{H^1(B')}^{2\varepsilon /(1+2\varepsilon )} \left( h^{-\varepsilon }\left\| \eta \zeta _h \right\| _{L^2(\varGamma )} + h^{-\varepsilon }\left\| \zeta _h \right\| _{H^{-1}(\varGamma )}\right) ^{1/(1+2\varepsilon )} \nonumber \\&{\mathop {\lesssim }\limits ^{(4.9)}} h^{\varepsilon /(1+2\varepsilon )} \left( \left\| \eta \zeta _h \right\| _{L^2(\varGamma )} +\left\| \zeta _h \right\| _{H^{-1}(\varGamma )}\right) . \end{aligned}$$

We apply (3.1), (since $\eta \equiv 0$ on $\partial B_{R_{\varOmega }}(0)$ only the boundary terms for $\varGamma$ appear) together with Young’s inequality $ab \le a^p/p + b^q/q$ applied with $p=(1+2\varepsilon )/2\varepsilon$, $q=1+2\varepsilon$ to obtain

$$\begin{aligned}&T_3 = \left\| u_{\text {near}} \right\| _{L^2(B')}^{2\varepsilon /(1+2\varepsilon )} \left\| \eta V\zeta _h \right\| _{H^{1+\varepsilon }(\varGamma )}^{1/(1+2\varepsilon )}\\&\quad {\mathop {\lesssim }\limits ^{(3.1), (4.8)}} \left\| \eta V\zeta _h \right\| _{H^{-\alpha _D}(\varGamma )}^{2\varepsilon /(1+2\varepsilon )} \left( h^{-\varepsilon }\left\| \eta \zeta _h \right\| _{L^{2}(\varGamma )}+h^{-\varepsilon }\left\| \zeta _h \right\| _{H^{-1}(\varGamma )}\right) ^{1/(1+2\varepsilon )} \\&\qquad \quad \lesssim h^{-1}\left\| \eta V\zeta _h \right\| _{H^{-\alpha _D}(\varGamma )} + h^{\varepsilon }\left\| \eta \zeta _h \right\| _{L^{2}(\varGamma )} +h^{\varepsilon }\left\| \zeta _h \right\| _{H^{-1}(\varGamma )}. \end{aligned}$$

Similarly, we get for the second term in (4.7)

$$\begin{aligned} T_2&= \left\| u_{\text {near}} \right\| _{L^2(B')}^{2\varepsilon /(1+2\varepsilon )} \left\| u_{\text {near}} \right\| _{H^1(B')}^{1/(1+2\varepsilon )}\\&{\mathop {\lesssim }\limits ^{(3.1)}} h^{-2\varepsilon /(1+2\varepsilon )} \left\| \eta V\zeta _h \right\| _{H^{-\alpha _D}(\varGamma )}^{2\varepsilon /(1+2\varepsilon )} h^{2\varepsilon /(1+2\varepsilon )}\left\| u_{\text {near}} \right\| _{H^1(B')}^{1/(1+2\varepsilon )} \\&{\mathop {\lesssim }\limits ^{(4.9)}} h^{-1}\left\| \eta V\zeta _h \right\| _{H^{-\alpha _D}(\varGamma )} + h^{2\varepsilon }\left\| \eta \zeta _h \right\| _{L^{2}(\varGamma )} + h^{2\varepsilon }\left\| \zeta _h \right\| _{H^{-1-\alpha _D}(\varGamma )}. \end{aligned}$$

Inserting everything in (4.7) and using $h\lesssim 1$ gives

$$\begin{aligned}&\left\| \partial _n u_{\text {near}} \right\| _{L^2({\widehat{\varGamma }})}\\&\quad \lesssim h^{\varepsilon /(1+2\varepsilon )}\left( \left\| \eta \zeta _h \right\| _{L^2(\varGamma )}+ \left\| \zeta _h \right\| _{H^{-1}(\varGamma )}\right) + h^{-1}\left\| \eta V\zeta _h \right\| _{H^{-\alpha _D}(\varGamma )}. \end{aligned}$$

Applying the same argument for the exterior Dirichlet boundary value problem leads to an estimate for the jump of the normal derivative

$$\begin{aligned} \left\| [\partial _n u_{\text {near}}] \right\| _{L^2({\widehat{\varGamma }})} \lesssim h^{\varepsilon /(1+2\varepsilon )}\left( \left\| \eta \zeta _h \right\| _{L^2(\varGamma )}+ \left\| \zeta _h \right\| _{H^{-1}(\varGamma )}\right) + h^{-1}\left\| \eta V\zeta _h \right\| _{H^{-\alpha _D}(\varGamma )}. \end{aligned}$$

It remains to estimate the far field $u_{\text {far}}$, which can be treated similarly to the near field using a trace estimate and Lemma 3.1. Applying Lemma 3.1 with a cut-off function ${\widetilde{\eta }}$ satisfying ${\widetilde{\eta }} \equiv 1$ on $B\cap \varGamma$ and , the boundary term in (3.2) disappears since ${\widetilde{\eta }}(1-\eta )\equiv 0$, which simplifies the arguments:

$$\begin{aligned} \left\| [\partial _n u_{\text {far}}] \right\| _{L^2({\widehat{\varGamma }})}&\le \left\| [\partial _n({\widehat{\eta }}u_{\text {far}})] \right\| _{L^2({\widehat{\varGamma }})} \lesssim \left\| u_{\text {far}} \right\| _{H^{3/2+\varepsilon }(B)}\\&{\mathop {\lesssim }\limits ^{(3.2)}} \left\| u_{\text {far}} \right\| _{H^{1}(B')}+ \left\| {\widetilde{\eta }}(1-\eta )V\zeta _h \right\| _{H^{1+\varepsilon }(\varGamma )} =\left\| u_{\text {far}} \right\| _{H^{1}(B')} \\&\lesssim \left\| (1-\eta )V\zeta _h \right\| _{H^{1/2}(\varGamma \cup \partial B_{R_{\varOmega }}(0))} \lesssim \left\| \zeta _h \right\| _{H^{-1/2}(\varGamma )}, \end{aligned}$$

which proves the lemma. $\square$

We use the Galerkin projection $\varPi _V: H^{-1/2}(\varGamma ) \rightarrow S^{0,0}(\mathcal {T}_h)$, which is defined by

4.11 $$\begin{aligned} \left\langle V(\widehat{\phi } - \varPi _V\widehat{\phi }),\psi _h \right\rangle = 0 \quad \forall \psi _h \in S^{0,0}(\mathcal {T}_h). \end{aligned}$$

We denote by $I_h$ the $L^2(\varGamma )$-orthogonal projection given by

$$\begin{aligned} \left\langle I_h u ,v_h \right\rangle _{L^2(\varGamma )} = \left\langle u,v_h \right\rangle _{L^2(\varGamma )} \quad \forall v_h \in S^{0,0}(\mathcal {T}_h). \end{aligned}$$

The operator $I_h$ has the following super-approximation property, [18]: For any discrete function $\psi _h \in S^{0,0}(\mathcal {T}_h)$ and a cut-off function $\eta$, we have (with implied constants depending on $\Vert \eta \Vert _{W^{1,\infty }}$)4.12The following lemma provides an estimate for the local Galerkin error and includes the key steps to the proof of Theorem 2.3.

#### Lemma 4.3

Let the assumptions of Theorem 2.3 hold. Let $\widehat{\varGamma _0}$ be an open subset of $\varGamma$ with $\varGamma _0\subset \widehat{\varGamma _0} \subsetneq \varGamma$ and . Let *h* satisfy $\frac{h}{R}\le \frac{1}{12}$. Assume that $\phi \in L^{2}(\widehat{\varGamma _0})$. Then, we have for the Galerkin error $\phi - \phi _h = \phi - \varPi _V\phi$

$$\begin{aligned} \left\| \phi -\phi _h \right\| _{L^{2}(\varGamma _0)}\le & {} C \left( \inf _{\chi _h\in S^{0,0}(\mathcal {T}_h)}\left\| \phi - \chi _h \right\| _{L^{2}(\widehat{\varGamma _0})} +\,\left\| \phi -\phi _h \right\| _{H^{-1/2}(\widehat{\varGamma _0})}\right. \\&\quad +\left. h^{\alpha _D/(1+2\alpha _D)}\left\| \phi -\phi _h \right\| _{L^{2}(\widehat{\varGamma _0})}+ \left\| \phi -\phi _h \right\| _{H^{-1-\alpha _D}(\varGamma )}\right) . \end{aligned}$$

The constant $C>0$ depends only on $\varGamma ,\varGamma _0,d,R,$ and the $\gamma$-shape regularity of $\mathcal {T}_h$.

#### Proof:

We define $e{:}{=} \phi -\phi _h$, open subsets $\varGamma _0\subset \varGamma _1\subset \varGamma _2 \dots \subset \varGamma _5 \subset \widehat{\varGamma _0}$, and volume boxes $B_0 \subset B_1 \subset B_2 \dots \subset B_5 \subset {\mathbb {R}}^d$, where $B_i\cap \widehat{\varGamma }_0 = \varGamma _i$. Throughout the proof, we use cut-off functions $\eta _i \in C_0^{\infty }({\mathbb {R}}^d)$, $i=1,\dots ,5$, satisfying $\eta _i \equiv 1$ on $\varGamma _{i-1}$,  and $\left\| \nabla \eta _i \right\| _{L^{\infty }(B_i)}\lesssim \frac{1}{R}$. We write

4.13 $$\begin{aligned} \left\| e \right\| _{L^2(\varGamma _0)}^2\le \left\| \eta _1 e \right\| _{L^2(\varGamma )}^2 = \left\langle \eta _1 e, \eta _1 e \right\rangle = \left\langle e,\eta _1^2 e \right\rangle . \end{aligned}$$

With the Galerkin projection $\varPi _V$ from (4.11), we obtain

4.14 $$\begin{aligned} \left\| \eta _1 e \right\| ^2_{L^2(\varGamma )}= & {} \left\langle e,\eta _1^2 e \right\rangle = \left\langle \eta _5e,\eta _1^2 e \right\rangle \nonumber \\= & {} \left\langle \varPi _V(\eta _5 e),\eta _1^2 e \right\rangle + \left\langle \eta _5 e-\varPi _V(\eta _5 e),\eta _1^2 e \right\rangle . \end{aligned}$$

With an inverse inequality and the $L^2$-orthogonal projection $I_h$, which satisfies the super-approximation property (4.12) for $\eta _5 \phi _h$, we get

4.15 $$\begin{aligned}&\left\| \eta _5 \phi _h - \varPi _V(\eta _5\phi _h) \right\| _{L^2(\varGamma )} \lesssim \left\| \eta _5 \phi _h- I_h(\eta _5\phi _h) \right\| _{L^2(\varGamma )} + \left\| I_h(\eta _5 \phi _h)- \varPi _V(\eta _5\phi _h) \right\| _{L^2(\varGamma )}\nonumber \\&\quad \lesssim h\left\| \phi _h \right\| _{L^2(\widehat{\varGamma _0})} + h^{-1/2}\left\| I_h(\eta _5 \phi _h)- \varPi _V(\eta _5\phi _h) \right\| _{H^{-1/2}(\varGamma )}\nonumber \\&\quad \lesssim h\left\| \phi _h \right\| _{L^2(\widehat{\varGamma _0})} + h^{-1/2}\left\| I_h(\eta _5 \phi _h)- \eta _5\phi _h \right\| _{H^{-1/2}(\varGamma )}\nonumber \\&\qquad + h^{-1/2}\left\| \eta _5 \phi _h- \varPi _V(\eta _5\phi _h) \right\| _{H^{-1/2}(\varGamma )} \nonumber \\&\quad \lesssim h\left\| \phi _h \right\| _{L^2(\widehat{\varGamma _0})}, \end{aligned}$$

where the last estimate follows from Céa’s lemma and super-approximation. The same argument leads to

4.16 $$\begin{aligned} \left\| \eta _5 \phi - \varPi _V(\eta _5\phi ) \right\| _{L^2(\varGamma )}\lesssim & {} \left\| \eta _5 \phi - I_h(\eta _5\phi ) \right\| _{L^2(\varGamma )} + \left\| I_h(\eta _5 \phi )- \varPi _V(\eta _5\phi ) \right\| _{L^2(\varGamma )}\nonumber \\\lesssim & {} \left\| \eta _5 \phi \right\| _{L^2(\varGamma )} + h^{-1/2} \left\| I_h(\eta _5 \phi )- \varPi _V(\eta _5\phi ) \right\| _{H^{-1/2}(\varGamma )}\nonumber \\\lesssim & {} \left\| \eta _5 \phi \right\| _{L^2(\varGamma )}. \end{aligned}$$

In fact, this argument shows $L^2$-stability of $\varPi _V$:

4.17 $$\begin{aligned} \Vert \varPi _V\psi \Vert _{L^2(\varGamma )} \lesssim \Vert \psi \Vert _{L^2(\varGamma )} \qquad \forall \psi \in L^2(\varGamma ). \end{aligned}$$

The bounds (4.15), (4.16) together imply

4.18 $$\begin{aligned}&\left| \left\langle \eta _5 e-\varPi _V(\eta _5 e),\eta _1^2 e \right\rangle \right| \nonumber \\&\quad \le \left\| \eta _1^2 e \right\| _{L^2(\varGamma )}\left( \left\| \eta _5 \phi -\varPi _V(\eta _5 \phi ) \right\| _{L^2(\varGamma )} + \left\| \eta _5 \phi _h-\varPi _V(\eta _5 \phi _h) \right\| _{L^2(\varGamma )} \right) \nonumber \\&\quad \lesssim \left\| \eta _1 e \right\| _{L^2(\varGamma )}\left( \left\| \eta _5 \phi \right\| _{L^2(\varGamma )} + h\left\| \phi _h \right\| _{L^2(\widehat{\varGamma _0})}\right) \nonumber \\&\quad \lesssim \left\| \eta _1 e \right\| _{L^2(\varGamma )}\left( (1+h)\left\| \phi \right\| _{L^2(\widehat{\varGamma _0})} + h\left\| e \right\| _{L^2(\widehat{\varGamma _0})}\right) .\nonumber \\ \end{aligned}$$

For the first term on the right-hand side of (4.14), we want to use Lemma 4.2. Since $[\partial _n{\widetilde{V}}\zeta _h] = -\zeta _h \in S^{0,0}(\mathcal {T}_h)$ for any discrete function $\zeta _h \in S^{0,0}(\mathcal {T}_h)$, we need to construct a discrete function satisfying the orthogonality condition (4.2). Using the Galerkin orthogonality with test functions $\psi _h$ with support  and noting that $\eta _5 \equiv 1$ on , we obtain with the commutator $C_{\eta _5}$ defined in (3.5)

4.19 $$\begin{aligned} 0= & {} \left\langle Ve,\eta _5\psi _h \right\rangle = \left\langle \eta _5Ve,\psi _h \right\rangle = \left\langle V(\eta _5e)-C_{\eta _5}e,\psi _h \right\rangle \nonumber \\= & {} \left\langle V(\eta _5e)-\eta _5C_{\eta _5}e,\psi _h \right\rangle = \left\langle V(\eta _5e-V^{-1}(\eta _5C_{\eta _5}e)),\psi _h \right\rangle \nonumber \\= & {} \left\langle V(\varPi _V(\eta _5e)-\varPi _V(V^{-1}(\eta _5C_{\eta _5}e))),\psi _h \right\rangle . \end{aligned}$$

Thus, defining

4.20 $$\begin{aligned} \zeta _h {:}{=} \varPi _V(\eta _5e) - \xi _h \quad \text {with} \quad \xi _h{:}{=}\varPi _V(V^{-1}(\eta _5C_{\eta _5}e)), \end{aligned}$$

we get on the volume box $B_4\subset {\mathbb {R}}^d$ a discrete harmonic function

$$\begin{aligned} u{:}{=}{\widetilde{V}}\zeta _h \in {\mathcal H}_h(B_4). \end{aligned}$$

The correction $\xi _h$ can be estimated using the $L^2$-stability (4.17) of the Galerkin projection, the mapping properties of $V^{-1}$, $C_{\eta _5}$, $C_{\eta _5}^{\eta _5}$ from Lemma 3.3 by

4.21 $$\begin{aligned} \left\| \xi _h \right\| _{L^2(\varGamma )}= & {} \left\| \varPi _V(V^{-1}(\eta _5C_{\eta _5}e)) \right\| _{L^2(\varGamma )} \lesssim \left\| V^{-1}(\eta _5C_{\eta _5}e) \right\| _{L^2(\varGamma )} \lesssim \left\| \eta _5C_{\eta _5}e \right\| _{H^{1}(\varGamma )}\nonumber \\\lesssim & {} \left\| C_{\eta _5}(\eta _5e) \right\| _{H^{1}(\varGamma )}+\left\| C_{\eta _5}^{\eta _5}e \right\| _{H^{1}(\varGamma )} \lesssim \left\| \eta _5e \right\| _{H^{-1}(\varGamma )}+\left\| e \right\| _{H^{-1-\alpha _D}(\varGamma )}.\nonumber \\ \end{aligned}$$

We write

4.22 $$\begin{aligned} \left\langle \varPi _V(\eta _5 e),\eta _1^2 e \right\rangle= & {} \left\langle \varPi _V(\eta _5 e) -\xi _h,\eta _1^2 e \right\rangle + \left\langle \xi _h,\eta _1^2 e \right\rangle \nonumber \\= & {} \left\langle \zeta _h,\eta _1^2 e \right\rangle + \left\langle \xi _h,\eta _1^2e \right\rangle . \end{aligned}$$

For the second term in (4.22) we estimate

4.23 $$\begin{aligned}&\left| \left\langle \xi _h,\eta _1^2 e \right\rangle \right| \le \left\| \xi _h \right\| _{L^2(\varGamma )}\left\| \eta _1^2 e \right\| _{L^2(\varGamma )}\nonumber \\&\qquad \qquad \;\; {\mathop { \lesssim }\limits ^{(4.21)}} \left( \left\| \eta _5e \right\| _{H^{-1}(\varGamma )}+\left\| e \right\| _{H^{-1- \alpha _D}(\varGamma )}\right) \left\| \eta _1 e \right\| _{L^2(\varGamma )}. \end{aligned}$$

We treat the first term in (4.22) as follows: We apply Lemma 4.2 with the boxes $B_2$ and $B_3$ (note that, since we assumed $12 h\le R$, the condition  can be fulfilled) to the discrete harmonic function $u :={\widetilde{V}}\zeta _h \in {\mathcal H}_h(B_4)$ and the cut-off function $\eta _4$. The jump condition $[\partial _n u] = -\zeta _h$ leads to4.24The definition of $\zeta _h$, the bound (4.21), and the $H^{-1/2}$-stability of the Galerkin projection lead to

4.25 $$\begin{aligned} \left\| \zeta _h \right\| _{H^{-1/2}(\varGamma )}\lesssim & {} \left\| \eta _5e \right\| _{H^{-1/2}(\varGamma )} + \left\| \xi _h \right\| _{H^{-1/2}(\varGamma )}\nonumber \\\lesssim & {} \left\| \eta _5e \right\| _{H^{-1/2}(\varGamma )}+ \left\| e \right\| _{H^{-1-\alpha _D}(\varGamma )}. \end{aligned}$$

With the $L^2$-stability (4.17) of the Galerkin projection and (4.21) we get

4.26 $$\begin{aligned} \left\| \zeta _h \right\| _{L^{2}(\varGamma )}\lesssim & {} \left\| \eta _5e \right\| _{L^{2}(\varGamma )} + \left\| \xi _h \right\| _{L^{2}(\varGamma )}\nonumber \\\lesssim & {} \left\| \eta _5e \right\| _{L^{2}(\varGamma )} + \left\| e \right\| _{H^{-1-\alpha _D}(\varGamma )}. \end{aligned}$$

We use the orthogonality (4.19) satisfied by $\zeta _h$ on $\varGamma _4$, the $L^2$-orthogonal projection $I_h$ and the properties of the commutator $C_{\eta _5}$ given by Lemma 3.3 to arrive at

4.27 $$\begin{aligned}&\left\| \eta _4 V\zeta _h \right\| _{H^{-\alpha _D}(\varGamma )} = \sup _{w\in H^{\alpha _D}(\varGamma )}\frac{\left\langle V\zeta _h,\eta _4 w \right\rangle }{\left\| w \right\| _{H^{\alpha _D}(\varGamma )}} = \sup _{w\in H^{\alpha _D}(\varGamma )} \frac{\left\langle V\zeta _h,\eta _4w-I_h(\eta _4w) \right\rangle }{\left\| w \right\| _{H^{\alpha _D} (\varGamma )}}\nonumber \\&\quad \lesssim \sup _{w\in H^{\alpha _D}(\varGamma )}\frac{\left\| \eta _5V\zeta _h \right\| _{H^{1}(\varGamma )} \left\| \eta _4w-I_h(\eta _4w) \right\| _{H^{-1}(\varGamma )}}{\left\| w \right\| _{H^{\alpha _D}(\varGamma )}} \nonumber \\&\quad \lesssim h^{1+\alpha _D}\left( \left\| \eta _5\zeta _h \right\| _{L^{2}(\varGamma )} +\left\| C_{\eta _5}\zeta _h \right\| _{H^1(\varGamma )}\right) \nonumber \\&\quad \lesssim h^{1+\alpha _D}\left( \left\| \eta _5\zeta _h \right\| _{L^{2}(\varGamma )} +\left\| \zeta _h \right\| _{H^{-1}(\varGamma )}\right) . \end{aligned}$$

Inserting (4.25)–(4.27) in (4.24) and using $h\lesssim 1$, we arrive at4.28Combining (4.14), (4.22) with (4.18), (4.23), (4.28), and finally (4.13), we get

$$\begin{aligned}&\left\| \eta _1 e \right\| _{L^2(\varGamma )}^2 \\&\quad \lesssim \left( \left\| \phi \right\| _{L^2(\widehat{\varGamma _0})}+ \left\| e \right\| _{H^{-1/2}(\widehat{\varGamma _0})}+ h^{\alpha _D/(1+2\alpha _D)}\left\| e \right\| _{L^{2}(\widehat{\varGamma _0})} +\left\| e \right\| _{H^{-1-\alpha _D}(\varGamma )}\right) \left\| \eta _1 e \right\| _{L^2(\varGamma )}. \end{aligned}$$

Since we only used the Galerkin orthogonality as a property of the error $\phi -\phi _h$, we may write $\phi -\phi _h = (\phi -\chi _h)+(\chi _h - \phi _h)$ for arbitrary $\chi _h \in S^{0,0}(\mathcal {T}_h)$, and we have proven the inequality claimed in Lemma 4.3. $\square$

In order to prove Theorem 2.3, we need a lemma:

#### Lemma 4.4

For every $\delta > 0$ there is a bounded linear operator $J_\delta :H^{-1}(\varGamma ) \rightarrow L^{2}(\varGamma )$ with the following properties:

(i) (stability): For every $-1 \le s \le t \le 0$ there is $C_{s,t} > 0$ (depending only on *s*, *t*, $\varOmega$) such that $\Vert J_\delta u\Vert _{H^{t}(\varGamma )} \le \delta ^{s-t} C_s\Vert J_\delta u\Vert _{H^s(\varGamma )}$ for all $u \in H^s(\varGamma )$.
(ii) (locality): for $\omega \subset \varGamma$ the restriction $(J_\delta u)|_\omega$ depends only on $u|_{\omega _{\delta }}$ with $\omega _\delta {:}{=} \cup _{x \in \omega } B_\delta (x) \cap \varGamma$.
(iii) (approximation): For every $-1 \le t \le s \le 1$ there is $C_{s,t} > 0$ (depending only on *s*, *t*, $\varOmega$) such that $\Vert u - J_\delta u\Vert _{H^{t}(\varGamma )} \le C_{s,t} \delta ^{s-t} \Vert u\Vert _{H^s(\varGamma )}$ for all $u \in H^s(\varGamma )$.

#### Proof:

Operators with such properties are obtained by the usual mollification procedure (on a length scale $O(\delta )$ for domains in ${\mathbb {R}}^d$). This technique can be generalized to the present setting of surfaces with the aid of localization and charts. We also mention [1, 7] where similar operators mapping into $S^{1,1}({{\mathcal {T}}}_h)$ are constructed. $\square$

We are in position to prove our main result, a local estimate for the Galerkin-boundary element error for Symm’s integral equation in the $L^2$-norm.

#### Proof

(*of Theorem* 2.3): Starting with Lemma 4.3, it remains to estimate the two terms $h^{\alpha _D/(1+2\alpha _D)}\left\| e \right\| _{L^{2}(\widehat{\varGamma _0})}$ and $\left\| e \right\| _{H^{-1/2}(\widehat{\varGamma _0})}$, where $e{:}{=} \phi -\phi _h$.

We start with the latter. Let ${\widehat{\eta }} \in C^{\infty }({\mathbb {R}}^d)$ be a cut-off-function with ${\widehat{\eta }} \equiv 1$ on $\widehat{\varGamma _0}$,  and $\left\| \nabla {\widehat{\eta }} \right\| _{L^{\infty }}\lesssim \frac{1}{R}$. Let ${\widetilde{\eta }}$ be another cut-off function with ${\widetilde{\eta }}=1$ on $B^{\widehat{\varGamma _0}}_{R/2+h}\cap \varGamma$ and , where . Select $\delta = ch$ with a constant $c = O(1)$ such that the operator $J_{ch}$ of Lemma 4.4 has the support property . We will employ the operator $I_h \circ J_{ch}:H^{-1}(\varGamma ) \rightarrow S^{0,0}(\varGamma )$ with the $L^2$-orthogonal projection $I_h$. It is easy to see that we may assume that4.29Concerning the approximation properties, we have

4.30 $$\begin{aligned}&\Vert u - I_h \circ J_{ch} u\Vert _{H^{-1}(\varGamma )} \le \Vert u - J_{ch} u\Vert _{H^{-1}(\varGamma )} + \Vert J_{ch} u - I_h \circ J_{ch} u\Vert _{H^{-1}(\varGamma )} \nonumber \\&\qquad \lesssim (ch)^{1/2} \Vert u\Vert _{H^{-1/2}(\varGamma )} + h \Vert J_{ch} u\Vert _{L^2(\varGamma )} \lesssim h^{1/2} \Vert u\Vert _{H^{-1/2}(\varGamma )}. \end{aligned}$$

With the definition of the commutators $C_{{\widehat{\eta }}}$, $C_{\widehat{\eta }}^{{\widehat{\eta }}}$, the Galerkin orthogonality satisfied by *e*, and the fact that $V:H^{-1/2}(\varGamma ) \rightarrow H^{1/2}(\varGamma )$ is an isomorphism, we get

4.31 $$\begin{aligned}&\left\| {\widehat{\eta }}e \right\| _{H^{-1/2}(\varGamma )} = \sup _{w\in H^{1/2}(\varGamma )} \frac{\left\langle {\widehat{\eta }}e,w \right\rangle }{\left\| w \right\| _{H^{1/2}(\varGamma )}} \lesssim \sup _{\psi \in H^{-1/2}(\varGamma )}\frac{\left\langle {\widehat{\eta }}e,V\psi \right\rangle }{\left\| \psi \right\| _{H^{-1/2}(\varGamma )}} \nonumber \\&\quad = \sup _{\psi \in H^{-1/2}(\varGamma )}\frac{\left\langle Ve,{\widehat{\eta }}\psi \right\rangle +\left\langle C_{\widehat{\eta }}e,\psi \right\rangle }{\left\| \psi \right\| _{H^{-1/2}(\varGamma )}} \nonumber = \sup _{\psi \in H^{-1/2}(\varGamma )}\frac{\left\langle Ve,{\widehat{\eta }}\psi -I_h\circ J_{ch} ({\widehat{\eta }}\psi ) \right\rangle +\left\langle C_{\widehat{\eta }}e,\psi \right\rangle }{\left\| \psi \right\| _{H^{-1/2}(\varGamma )}} \nonumber \\&\quad {\mathop {=}\limits ^{(4.29)}} \sup _{\psi \in H^{-1/2}(\varGamma )}\frac{\left\langle {\widetilde{\eta }}Ve,{\widehat{\eta }} \psi -I_h\circ J_{ch}({\widehat{\eta }}\psi ) \right\rangle + \left\langle C_{{\widehat{\eta }}}e,\psi \right\rangle }{\left\| \psi \right\| _{H^{-1/2}(\varGamma )}} \nonumber \\&\quad = \sup _{\psi \in H^{-1/2}(\varGamma )}\frac{\left\langle V({\widetilde{\eta }}e),{\widehat{\eta }}\psi -I_h\circ J_{ch}({\widehat{\eta }}\psi ) \right\rangle - \left\langle C_{{\widetilde{\eta }}}e,{\widehat{\eta }}\psi -I_h\circ J_{ch}({\widehat{\eta }}\psi ) \right\rangle -\left\langle e,C_{{\widehat{\eta }}}\psi \right\rangle }{\left\| \psi \right\| _{H^{-1/2}(\varGamma )}}\nonumber \\&\quad \lesssim \sup _{\psi \in H^{-1/2}(\varGamma )}\frac{\left( \left\| {\widetilde{\eta }}e \right\| _{L^{2}(\varGamma )} +\left\| e \right\| _{H^{-1-\alpha _D}(\varGamma )} \right) \left\| {\widehat{\eta }}\psi -I_h\circ J_{ch}({\widehat{\eta }}\psi ) \right\| _{H^{-1}(\varGamma )}+ \left\| e \right\| _{H^{-1-\alpha _D}(\varGamma )}\left\| \psi \right\| _{H^{-1+\alpha _D} (\varGamma )}}{\left\| \psi \right\| _{H^{-1/2}(\varGamma )}} \nonumber \\&\quad \lesssim h^{1/2}\left\| e \right\| _{L^{2} (\widehat{\varGamma _1})}+\left\| e \right\| _{H^{-1-\alpha _D}(\varGamma )}. \end{aligned}$$

The first term on the right-hand side of (4.31) can be treated in the same way as the term $h^{\alpha _D/(1+2\alpha _D)}\left\| e \right\| _{L^{2}(\widehat{\varGamma _1})}$ on the right-hand side of Lemma 4.3, which is treated by iterating the $L^2$-estimate of the statement of Theorem 4.3. That is, we set $m{:}{=}\lceil \frac{(1+\alpha _D)(1+2\alpha _D)}{\alpha _D} \rceil$. The assumption $C_{\alpha _D}\frac{h}{R}\le \frac{1}{12}$ allows us to define *m* nested domains $\widehat{\varGamma _i}$, $i=0,\dots ,m-1$ such that , $\widehat{\varGamma }_m\subset {\widehat{\varGamma }}$. Since the term $h^{\alpha _D/(1+2\alpha _D)}\left\| e \right\| _{L^{2}(\widehat{\varGamma _1})}$ again contains a local $L^2$-norm, we may use Lemma 4.3 and (4.31) again on the larger set $\widehat{\varGamma _2}\subsetneq \varGamma$ to estimate

$$\begin{aligned} h^{\alpha _D/(1+2\alpha _D)}\left\| e \right\| _{L^{2}(\widehat{\varGamma _1})}\lesssim & {} h^{\alpha _D/(1+2\alpha _D)}\left( \inf _{\chi _h\in S^{0,0}(\mathcal {T}_h)}\left\| \phi - \chi _h \right\| _{L^{2}(\widehat{\varGamma _2})}\right. \\&\qquad \qquad \qquad +\left. h^{\alpha _D/(1+2\alpha _D)}\left\| e \right\| _{L^{2}(\widehat{\varGamma _2})}+ \left\| e \right\| _{H^{-1-\alpha _D}(\varGamma )}\right) . \end{aligned}$$

Inserting this in the initial estimate of Lemma 4.3 (using $h\lesssim 1$) leads to

$$\begin{aligned} \left\| e \right\| _{L^{2}(\varGamma _0)}\le & {} C \Big ( \inf _{\chi _h\in S^{0,0}(\mathcal {T}_h)} \left\| \phi - \chi _h \right\| _{L^{2}(\widehat{\varGamma _2})} \\&+ h^{2\alpha _D/(1+2\alpha _D)}\left\| \phi -\phi _h \right\| _{L^{2}(\widehat{\varGamma _2})}+ \left\| \phi -\phi _h \right\| _{H^{-1-\alpha _D}(\varGamma )}\Big ). \end{aligned}$$

Now, the $L^2$-term on the right-hand side is multiplied by $h^{2\alpha _D/(1+2\alpha _D)}$, i.e., the square of the initial factor. Iterating this argument $m-2$-times, provides the factor $h^{m\alpha _D/(1+2\alpha _D)}$, and by the choice of *m*, we have $h^{1+\alpha _D}\le h^{m\alpha _D/(1+2\alpha _D)}$. Together with an inverse estimate, we obtain

$$\begin{aligned} h^{1+\alpha _D}\left\| e \right\| _{L^2({\widehat{\varGamma }})}\le & {} h^{1+\alpha _D}\left\| \phi -\chi _h \right\| _{L^2({\widehat{\varGamma }})} + h^{1+\alpha _D}\left\| \phi _h-\chi _h \right\| _{L^2({\widehat{\varGamma }})}\\\lesssim & {} h^{1+\alpha _D}\left\| \phi -\chi _h \right\| _{L^2({\widehat{\varGamma }})} + \left\| \phi _h-\chi _h \right\| _{H^{-1-\alpha _D}({\widehat{\varGamma }})}\\\lesssim & {} h^{1+\alpha _D}\left\| \phi -\chi _h \right\| _{L^2({\widehat{\varGamma }})} + \left\| e \right\| _{H^{-1-\alpha _D}({\widehat{\varGamma }})}+\left\| \phi -\chi _h \right\| _{H^{-1-\alpha _D}({\widehat{\varGamma }})} \\\lesssim & {} \left\| \phi -\chi _h \right\| _{L^{2}({\widehat{\varGamma }})} + \left\| e \right\| _{H^{-1-\alpha _D}(\varGamma )}, \end{aligned}$$

which proves the theorem. $\square$

#### Proof

(*of Corollary* 2.4): The assumption $\phi \in H^{-1/2+\alpha }(\varGamma ) \cap H^{\beta }(\widetilde{\varGamma })$ leads to

$$\begin{aligned} \inf _{\chi _h \in S^{0,0}(\mathcal {T}_h)}\left\| \phi -\chi _h \right\| _{L^{2}({\widehat{\varGamma }})}\lesssim & {} h^{\beta }\left\| \phi \right\| _{H^{\beta }(\widetilde{\varGamma })}, \\ \left\| e \right\| _{H^{-1/2}(\varGamma )}\lesssim & {} h^{\alpha }\left\| \phi \right\| _{H^{-1/2+\alpha }(\varGamma )}, \end{aligned}$$

where the second estimate is the standard global error estimate for the BEM, see [22].

It remains to estimate $\left\| e \right\| _{H^{-1-\alpha _D}(\varGamma )}$, which is treated with a duality argument: We note that Assumption 2.1 and the jump relations imply the following shift theorem for *V*: If $w \in H^{1+\alpha _D}(\varGamma )$ and $\psi$ solves $V\psi = w \in H^{1+\alpha _D}(\varGamma )$, then $\psi \in H^{\alpha _D}(\varGamma )$ and $\Vert \psi \Vert _{H^{\alpha _D}(\varGamma )} \lesssim \Vert w\Vert _{H^{1+\alpha _D}(\varGamma )}$. Hence, with the Galerkin projection $\varPi _V$, we estimate

$$\begin{aligned} \left\| e \right\| _{H^{-1-\alpha _D}(\varGamma )}= & {} \sup _{w\in H^{1+\alpha _D}(\varGamma )}\frac{\left\langle e,w \right\rangle }{\left\| w \right\| _{H^{1+\alpha _D}(\varGamma )}} \lesssim \sup _{\psi \in H^{\alpha _D}(\varGamma )}\frac{\left| \left\langle e,V\psi \right\rangle \right| }{\left\| \psi \right\| _{H^{\alpha _D}(\varGamma )}} \nonumber \\= & {} \sup _{\psi \in H^{\alpha _D}(\varGamma )}\frac{\left| \left\langle Ve,\psi -\varPi _V\psi \right\rangle \right| }{\left\| \psi \right\| _{H^{\alpha _D}(\varGamma )}} \\\lesssim & {} \sup _{\psi \in H^{\alpha _D}(\varGamma )}\frac{\left\| Ve \right\| _{H^{1/2}(\varGamma )} \left\| \psi -\varPi _V\psi \right\| _{H^{-1/2}(\varGamma )}}{\left\| \psi \right\| _{H^{\alpha _D}(\varGamma )}} \lesssim h^{1/2+\alpha _D} \left\| e \right\| _{H^{-1/2}(\varGamma )} \\\lesssim & {} h^{1/2+\alpha +\alpha _D} \left\| \phi \right\| _{H^{-1/2+\alpha }(\varGamma )}. \end{aligned}$$

Therefore, the term of slowest convergence is of order $\mathcal {O}(h^{\min \{1/2+\alpha +\alpha _D,\beta \}})$, which proves the corollary. $\square$

#### Remark 4.5

The term of slowest convergence in the case of high local regularity is the global error in the negative $H^{-1-\alpha _D}(\varGamma )$-norm, which is treated with a duality argument that uses the maximum amount of additional regularity on the polygonal/polyhedral domain. Therefore, further improvements of the convergence rate cannot be achieved with our method of proof. In fact, the numerical examples in the next section confirm the sharpness of this observation, i.e., that the best possible convergence is $\mathcal {O}(h^{1/2+\alpha +\alpha _D})$.

The trivial estimate $\left\| \eta e \right\| _{H^{-1/2}(\varGamma )} \lesssim \left\| \eta e \right\| _{L^{2}(\varGamma )}$ immediately implies that the local convergence in the energy norm is at least of order $\mathcal {O}(h^{1/2+\alpha +\alpha _D})$ as well. Again, analyzing the proof of Lemma 4.3, we observe that an improvement is impossible, since the limiting term is once more the error in the negative $H^{-1-\alpha _D}(\varGamma )$-norm. $\square$

#### Remark 4.6

Remark 4.5 states that the local rate of convergence is limited by the shift theorem of Assumption 2.1. If the geometry $\varOmega$ is smooth, then elliptic shift theorems for the Dirichlet problem hold in a wider range, e.g., if $f\in H^{1/2}(\varOmega )$, we may get $u \in H^{5/2}(\varOmega )$. It can be checked that in this setting, an estimate of the form

$$\begin{aligned} \Vert \phi - \phi _h\Vert _{L^2(\varGamma _0)} \lesssim \inf _{\chi _h \in S^{0,0}({{\mathcal {T}}}_h)} \Vert \phi - \chi \Vert _{L^2({\widehat{\varGamma }})} + \Vert \phi - \phi _h\Vert _{H^{-2}(\varGamma )} \end{aligned}$$

is possible since the commutator $C_{\eta _5}^{\eta _5}$ in (4.21) then maps $H^{-2}(\varGamma )\rightarrow H^1(\varGamma )$. If an even better shift theorem holds, then the $H^{-2}$-norm can be further weakened by using commutators of higher order. The best possible achievable local rates are then $O(h^\beta )$ in $L^2(\varGamma _0)$ for $\phi \in H^\beta ({\widehat{\varGamma }})$, $\beta \in [0,1]$ and $O(h^{1/2+\beta })$ in the $H^{-1/2}(\varGamma _0)$-norm.

$$\square $$

### The hyper-singular integral equation (proof of Theorem 2.8)

We start with the Galerkin orthogonality

4.32 $$\begin{aligned} \left\langle W(\varphi -\varphi _h),\psi _h \right\rangle + \left\langle \varphi -\varphi _h,1 \right\rangle \left\langle \psi _h,1 \right\rangle = 0 \quad \forall \psi _h \in S^{1,1}(\mathcal {T}_h) \end{aligned}$$

and a Caccioppoli-type estimate on $D\subset {\mathbb {R}}^d$ for functions characterized by the orthogonality4.33for some $\mu \in {\mathbb {R}}$. Here, we define the space of discrete harmonic functions ${\mathcal H}^{{\mathcal {N}}}_{h}(D,\mu )$ for an open set $D\subset {\mathbb {R}}^d$ and $\mu \in {\mathbb {R}}$ as

4.34 $$\begin{aligned}&{\mathcal H}^{{\mathcal {N}}}_{h}(D,\mu ){:}{=}\{v\in H^1(D \backslash \varGamma ) :v \text { is harmonic on}\; D\backslash \varGamma , [\partial _n v] = 0,\nonumber \\&\quad \quad \exists \widetilde{v} \in S^{1,1}({{\mathcal {T}}}_h) \;\text{ s.t. } \; [\gamma _0 v]|_{D \cap \varGamma } = \widetilde{v}|_{D \cap \varGamma }, \; v \text { satisfies}\; (4.33)\}. \end{aligned}$$

#### Proposition 4.7

[12, Lemma 3.8] For discrete harmonic functions $u \in {\mathcal H}^{{\mathcal {N}}}_{h}(B',\mu )$, we have the interior regularity estimate

4.35 $$\begin{aligned} \left\| \nabla u \right\| _{L^2(B\backslash \varGamma )} \lesssim \frac{h}{{\widehat{d}}}\left\| \nabla u \right\| _{L^2(B'\backslash \varGamma )} + \frac{1}{{\widehat{d}}}\left\| u \right\| _{L^2(B'\backslash \varGamma )} + \left| \mu \right| , \end{aligned}$$

where *B* and $B'$ are nested boxes and  satisfies $8h\le {\widehat{d}}$. The hidden constant depends only on $\varOmega , d$, and the $\gamma$-shape regularity of $\mathcal {T}_h$.

We use the Galerkin projection $\varPi _W: H^{1/2}(\varGamma ) \rightarrow S^{1,1}(\mathcal {T}_h)$, now defined by

4.36 $$\begin{aligned} \left\langle W(\varphi - \varPi _W\varphi ),\psi _h \right\rangle + \left\langle \varphi -\varPi _W\varphi ,1 \right\rangle \left\langle \psi _h,1 \right\rangle = 0 \quad \forall \psi _h \in S^{1,1}(\mathcal {T}_h). \end{aligned}$$

The following lemma collects approximation properties of the Galerkin projection that will be applied in both Lemmas 4.10 and 4.11 below.

#### Lemma 4.8

Let $\varPi _W$ be the Galerkin projection defined in (4.36), and let $\eta$, $\widehat{\eta } \in C_0^{\infty }(\mathbb {R}^d)$ be with $\widehat{\eta } \equiv 1$ on . For $\varphi \in H^1(\varGamma )$, we have for $s \in [1/2,1]$

4.37 $$\begin{aligned} \left| \eta \varphi - \varPi _W(\eta \varphi ) \right| _{H^{s}(\varGamma )} \le C \left| \eta \varphi \right| _{H^{s}(\varGamma )}. \end{aligned}$$

For $\varphi _h \in S^{1,1}(\mathcal {T}_h)$, we have for $s \in [1/2,1]$

4.38 $$\begin{aligned} \left| \eta \varphi _h - \varPi _W(\eta \varphi _h) \right| _{H^s(\varGamma )} \le C h\left\| \widehat{\eta } \varphi _h \right\| _{H^s(\varGamma )}. \end{aligned}$$

The constant $C>0$ depends only on $\varOmega$, the $\gamma$-shape regularity of  $\mathcal {T}_h$, and $\Vert \eta \Vert _{W^{2,\infty }(\mathbb {R}^d)}$.

#### Proof:

Let $\mathcal {J}_h$ be a quasi-interpolation operator with approximation properties in the $H^s$-seminorm, e.g., the Scott-Zhang-projection, [24]. We use super-approximation similarly to (4.12). Since $\varphi _h \in S^{1,1}(\mathcal {T}_h)$, we have to use the piecewise $H^2$-norm, and an inverse inequality leads towhere, in the last step, the assumption on $\widehat{\eta }$ was used. Similarly, the $H^1$-norm estimate $\left\| \eta \varphi _h - \mathcal {J}_h(\eta \varphi _h) \right\| _{H^1(\varGamma )} \lesssim h^{s}\left\| \widehat{\eta }\varphi _h \right\| _{H^s(\varGamma )}$ holds. Interpolation finally leads to a super-approximation result in $H^s$

$$\begin{aligned}&\left| \eta \varphi _h - \mathcal {J}_h(\eta \varphi _h) \right| _{H^s(\varGamma )} \lesssim h\left\| \widehat{\eta }\varphi _h \right\| _{H^s(\varGamma )}. \end{aligned}$$

With an inverse inequality, see, e.g., [13, Thm. 3.2], as well as Céa’s lemma this implies

$$\begin{aligned}&\left| \eta \varphi _h- \varPi _W(\eta \varphi _h) \right| _{H^{s}(\varGamma )} \lesssim \left| \eta \varphi _h- \mathcal {J}_h(\eta \varphi _h) \right| _{H^{s}(\varGamma )} + \left| \mathcal {J}_h(\eta \varphi _h)- \varPi _W(\eta \varphi _h) \right| _{H^{s}(\varGamma )} \\&\qquad \quad \lesssim h\left\| \widehat{\eta } \varphi _h \right\| _{H^{s}(\varGamma )} + h^{1/2-s}\left| \mathcal {J}_h(\eta \varphi _h)- \varPi _W(\eta \varphi _h) \right| _{H^{1/2}(\varGamma )}\nonumber \\&\qquad \quad \lesssim h\left\| \widehat{\eta } \varphi _h \right\| _{H^{s}(\varGamma )} + h^{1/2-s}\left| \mathcal {J}_h(\eta \varphi _h)- \eta \varphi _h \right| _{H^{1/2}(\varGamma )} \\ {}&\qquad \quad \quad + h^{1/2-s}\left| \eta \varphi _h- \varPi _W(\eta \varphi _h) \right| _{H^{1/2}(\varGamma )} \\&\qquad \quad \lesssim h\left\| \widehat{\eta } \varphi _h \right\| _{H^{s}(\varGamma )} + h^{1/2-s}\left| \mathcal {J}_h(\eta \varphi _h)- \eta \varphi _h \right| _{H^{1/2}(\varGamma )} \lesssim h\left\| \widehat{\eta } \varphi _h \right\| _{H^{s}(\varGamma )}. \end{aligned}$$

A similar argument leads to

$$\begin{aligned} \left| \eta \varphi - \varPi _W(\eta \varphi ) \right| _{H^{s}(\varGamma )}\lesssim & {} \left| \eta \varphi \right| _{H^{s}(\varGamma )}, \end{aligned}$$

and consequently to the $H^1$-stability of the Galerkin-projection. $\square$

In the following, we need stability and approximation properties of the Scott-Zhang projection ${\mathcal {J}}_h$ in the space $H^{1+\alpha _N}(\varGamma )$ provided by the following lemma.

#### Lemma 4.9

Let ${\mathcal {J}}_h$ be the Scott-Zhang projection defined in [24]. Then, for $s \in [0,3/2)$ we have

4.39 $$\begin{aligned} \Vert {{\mathcal {J}}}_h u\Vert _{H^{s}(\varGamma )} \le C_s \Vert u\Vert _{H^{s}(\varGamma )} \qquad \forall u \in H^s(\varGamma ), \end{aligned}$$

and therefore, for every $0 \le t \le s < 3/2$

4.40 $$\begin{aligned} \Vert u-{\mathcal {J}}_h u\Vert _{H^{t}(\varGamma )} \le C_{s,t} h^{s-t}\Vert u\Vert _{H^{s}(\varGamma )}. \end{aligned}$$

The constants $C_s$, $C_{s,t}>0$ depend only on $\varOmega$, the $\gamma$-shape regularity of $\mathcal {T}_h$, and *s*, *t*.

#### Proof:

We start with the proof of (4.39). The stability for the case $s = 1$ is given in [24] and the stability for the case $s = 0$ (note that $\varGamma$ is a closed surface without boundary) is discussed in [3, Lemma 7]. By interpolation, (4.39) follows for $0< s < 1$. The starting point for the proof of (4.39) for $s \in (1,3/2)$ is that, by Remark 1.1, (iii), we may focus on a single affine piece $\varGamma _i$ of $\varGamma$ and can exploit that the notion of $H^s(\varGamma _i)$ coincides with the standard notion on intervals (in 1D) and polygons (in 2D). In particular, $H^s(\varGamma _i)$ can be defined as the interpolation space between $H^1(\varGamma _i)$ and $H^2(\varGamma _i)$. Since ${{\mathcal {J}}}_h u \in C^0(\varGamma )$, Remark 1.1, (iii) implies for $s \in (1,3/2)$

$$\begin{aligned} \Vert {{{\mathcal {J}}}}_h u\Vert _{H^s(\varGamma )} \sim \sum _{i=1}^N \Vert {{{\mathcal {J}}}}_h u\Vert _{H^s(\varGamma _i)} \qquad \text{ and } \qquad \Vert u\Vert _{H^s(\varGamma )} \sim \sum _{i=1}^N \Vert u\Vert _{H^s(\varGamma _i)}. \end{aligned}$$

It therefore suffices to show $\Vert {{{\mathcal {J}}}}_h u\Vert _{H^s(\varGamma _i)} \le C \Vert u\Vert _{H^s(\varGamma _i)}$. Since $H^s(\varGamma _i)$ is an interpolation space between $H^1(\varGamma _i)$ and $H^2(\varGamma _i)$, we can find (cf. [4]), for every $t > 0$, a function $u_t \in H^2(\varGamma _i)$ with

4.41 $$\begin{aligned} \Vert u_t\Vert _{H^2(\varGamma _i)}\lesssim & {} t^{s-2} \Vert u\Vert _{H^{s}(\varGamma _i)}, \quad \Vert u_t\Vert _{H^{s}(\varGamma _i)} \lesssim \Vert u\Vert _{H^{s}(\varGamma _i)}\nonumber \\ \Vert u-u_t\Vert _{H^1(\varGamma _i)}\lesssim & {} t^{s-1}\Vert u\Vert _{H^{s}(\varGamma _i)}. \end{aligned}$$

Let $I_h'$ be an approximation operator with the simultaneous approximation property

4.42 $$\begin{aligned} \Vert u_t-I_h' u_t\Vert _{H^{s}(\varGamma _i)}+ h^{-(s-1)}\Vert u_t-I_h' u_t\Vert _{H^{1}(\varGamma _i)}\lesssim & {} h^{2-s} \Vert u_t\Vert _{H^2(\varGamma _i)}, \end{aligned}$$

see, e.g., [4, 6, Thm. 14.4.2]. With an inverse inequality, cf. [8, Appendix], the $H^1$-stability of the Scott-Zhang projection, and (4.41), (4.42), we estimate

$$\begin{aligned}&\Vert u-{\mathcal {J}}_h u\Vert _{H^{s}(\varGamma _i)} \\&\quad \lesssim \Vert u-u_t\Vert _{H^{s}(\varGamma _i)} + \Vert u_t- I_h' u_t\Vert _{H^{s}(\varGamma _i)} + \Vert {\mathcal {J}}_h(I_h'(u_t)- u_t)\Vert _{H^{s}(\varGamma _i)} \\&\qquad + \Vert {\mathcal {J}}_h(u- u_t)\Vert _{H^{s}(\varGamma _i)} \\&\quad \lesssim \Vert u-u_t\Vert _{H^{s}(\varGamma _i)} + \Vert u_t- I_h' u_t\Vert _{H^{s}(\varGamma _i)} + h^{-(s-1)}\Vert u_t- I_h' u_t\Vert _{H^{1}(\varGamma _i)} \nonumber \\&\qquad + h^{-(s-1)} \Vert u- u_t\Vert _{H^{1}(\varGamma _i)} \\&\quad {\mathop {\lesssim }\limits ^{(4.42)}} \Vert u-u_t\Vert _{H^{s}(\varGamma _i)} +h^{2-s}\Vert u_t\Vert _{H^2(\varGamma _i)} + h^{-(s-1)} \Vert u - u_t\Vert _{H^{1}(\varGamma _i)} \\&\quad {\mathop {\lesssim }\limits ^{(4.41)}} \Vert u\Vert _{H^{s}(\varGamma _i)} +h^{2-s}t^{s-2}\Vert u\Vert _{H^{s}(\varGamma _i)} + h^{-(s-1)}t^{s-1} \Vert u\Vert _{H^{s}(\varGamma _i)}. \end{aligned}$$

Choosing $t=\mathcal {O}(h)$, we get the $H^{s}(\varGamma _i)$-stability of ${\mathcal {J}}_h$ and thus also the $H^s(\varGamma )$-stability of ${{\mathcal {J}}}_h$.

We only prove the approximation property (4.40) for $s \in (1,3/2)$ as the case $s \in [0,1]$ is covered by standard properties of the Scott-Zhang operator.

*Case *$1 \le t \le s <3/2$: we observe with the stability properties of ${{{\mathcal {J}}}}_h$ and the approximation properties of $I_h'$

4.43 $$\begin{aligned} \Vert u - {{{\mathcal {J}}}}_h u\Vert _{H^t(\varGamma )}&\sim \sum _{i=1}^N \Vert u - {{{\mathcal {J}}}}_h u\Vert _{H^t(\varGamma _i)} \lesssim h^{s-t} \sum _{i=1}^N \Vert u\Vert _{H^s(\varGamma _i)} \sim h^{s-t} \Vert u\Vert _{H^s(\varGamma )}. \end{aligned}$$

*Case *$t=0$: we observe with the stability properties of ${{{\mathcal {J}}}}_h$ and the approximation properties of $I_h'$

4.44 $$\begin{aligned} \Vert u - {{{\mathcal {J}}}}_h u\Vert _{L^2(\varGamma )}&\sim \sum _{i=1}^N \Vert u - {{{\mathcal {J}}}}_h u\Vert _{L^2(\varGamma _i)} \lesssim h^{s} \sum _{i=1}^N \Vert u\Vert _{H^s(\varGamma _i)} \sim h^{s} \Vert u\Vert _{H^s(\varGamma )}. \end{aligned}$$

*Case *$0< t < 1$: The remaining cases are obtained with the aid of an interpolation inequality:

$$\begin{aligned}&\Vert u - {{{\mathcal {J}}}}_h u\Vert _{H^t(\varGamma )} \lesssim \Vert u - {{{\mathcal {J}}}}_h u\Vert ^{1-t}_{L^2(\varGamma )} \Vert u - {{{\mathcal {J}}}}_h u\Vert ^{t}_{H^1(\varGamma )}\\&\qquad \qquad {\mathop { \lesssim }\limits ^{(4.44), (4.43)}} h^{s(1-t)} h^{(s-1)t} \Vert u\Vert _{H^s(\varGamma )} = h^{s-t} \Vert u\Vert _{H^s(\varGamma )}, \end{aligned}$$

which concludes the proof. $\square$

The following lemma is similar to Lemma 4.2. Here, we obtain an estimate for the jump of the trace of a discrete harmonic double-layer potential.

#### Lemma 4.10

Let Assumption 2.7 hold and let $B \subset B' \subset B''$ be nested boxes with . Let *h* be sufficiently small so that the assumption of Proposition 4.7 holds. Let $u{:}{=}{\widetilde{K}}\zeta _h$ with $\zeta _h \in S^{1,1}(\mathcal {T}_h)$ and assume $u \in {\mathcal H}^{{\mathcal {N}}}_h(B'',\mu )$ for the box $B'' \subset B_{R_{\varOmega }}(0)$ and some $\mu \in {\mathbb {R}}$. Let ${\widehat{\varGamma }}\subset B\cap \varGamma$. Then,

4.45 $$\begin{aligned} \left| [\gamma _0 u] \right| _{H^1({\widehat{\varGamma }})} \le C \left( h^{\alpha _N}\left| \zeta _h \right| _{H^1(\varGamma )} + \left\| \zeta _h \right\| _{H^{1/2}(\varGamma )}+ \left| \mu \right| \right) . \end{aligned}$$

The constant $C>0$ depends only on $\varOmega ,{\widehat{d}}$, the $\gamma$-shape regularity of $\mathcal {T}_h$, and the constants appearing in Assumption 2.7.

#### Proof:

*Step 1 (Splitting into near and far-field):* Let $\eta \in C^{\infty }_0({\mathbb {R}}^d)$ satisfy $\eta \equiv 1$ on $B'\cap \varGamma$ and . Define the near-field $u_{\text {near}}$ and the far field $u_{\text {far}}$ as potentials $u_{\text {near}}{:}{=} {\widetilde{K}}v_h - \overline{{\widetilde{K}}v_h}$ with $\overline{{\widetilde{K}}v_h}{:}{=}\frac{1}{\left| \varOmega \right| }\int _{\varOmega }{{\widetilde{K}}v_h}$ and $u_{\text {far}}{:}{=}{\widetilde{K}}\nu _h-\overline{{\widetilde{K}}\nu _h}$, where $v_h, \nu _h \in S^{1,1}(\mathcal {T}_h)$ are BEM solutions of

$$\begin{aligned} \left\langle Wv_h,\psi _h \right\rangle= & {} \left\langle \eta W\zeta _h - \eta z, \psi _h \right\rangle \quad \forall \psi _h \in S^{1,1}(\mathcal {T}_h), \\ \left\langle W\nu _h,\psi _h \right\rangle= & {} \left\langle (1-\eta ) W\zeta _h + \eta z, \psi _h \right\rangle \quad \forall \psi _h \in S^{1,1}(\mathcal {T}_h), \end{aligned}$$

with $\left\langle v_h,1 \right\rangle =0=\left\langle \nu _h,1 \right\rangle$. Here, *z* is a function with $z\equiv \mu$ on $\varGamma \cap B'$ such that the compatibility condition $\left\langle \eta W\zeta _h - \eta z,1 \right\rangle =\left\langle (\eta -1)W\zeta _h - \eta z,1 \right\rangle = 0$ holds. Since $\left\langle W\zeta _h,1 \right\rangle =0$ such a function exists. More precisely, we choose $z \in L^2(\varGamma )$ to be the piecewise constant function

$$\begin{aligned} z{:}{=} \left\{ \begin{array}{ll} \mu &{}\quad \text {on } \varGamma \cap B' , \\ \frac{\left\langle \eta W\zeta _h,1 \right\rangle -\mu \int _{\varGamma \cap B'}\eta }{\int _{(B''\backslash B')\cap \varGamma }\eta } &{}\quad \text {otherwise}. \end{array} \right. \end{aligned}$$

The function $v_h+\nu _h$ solves

$$\begin{aligned} \left\langle W(v_h+\nu _h),\psi _h \right\rangle = \left\langle W\zeta _h, \psi _h \right\rangle \quad \forall \psi _h \in S^{1,1}(\mathcal {T}_h), \end{aligned}$$

which implies $v_h+\nu _h = \zeta _h + c$ for a constant *c*. Therefore, $v{:}{=}u_{\text {near}}+u_{\text {far}} = u + {\widetilde{K}}c - \overline{{\widetilde{K}}(v_h+\nu _h)}$. Since $[\gamma _0 {\widetilde{K}}c] = c$ this implies

$$\begin{aligned} \left| [\gamma _0u] \right| _{H^1({\widehat{\varGamma }})} = \left| [\gamma _0v] \right| _{H^1({\widehat{\varGamma }})} \lesssim \left| [\gamma _0 u_{\text {near}}] \right| _{H^1({\widehat{\varGamma }})}+\left| [\gamma _0u_{\text {far}}] \right| _{H^1({\widehat{\varGamma }})}. \end{aligned}$$

The definition of *z* and $\eta \equiv 1$ on $B' \cap \varGamma$ lead to

$$\begin{aligned} \left\| \eta (z-\mu ) \right\| _{L^2(\varGamma )}^2= & {} \int _{(B''\backslash B')\cap \varGamma }\eta ^2\left( \frac{\left\langle \eta W\zeta _h,1 \right\rangle -\mu \int _{\varGamma \cap B'}\eta }{\int _{(B''\backslash B')\cap \varGamma }\eta }-\mu \right) ^2\\= & {} \int _{(B''\backslash B')\cap \varGamma }\eta ^2\left( \frac{\left\langle \eta (W\zeta _h-\mu ),1 \right\rangle }{\int _{(B''\backslash B')\cap \varGamma }\eta }\right) ^2 \\\lesssim & {} \left| \left\langle \eta (W\zeta _h-\mu ),1 \right\rangle \right| ^2. \end{aligned}$$

Consequently, we obtain

4.46 $$\begin{aligned} \left\| \eta (z-\mu ) \right\| _{L^2(\varGamma )}\lesssim & {} \left| \left\langle \eta (W\zeta _h-\mu ),1 \right\rangle \right| \lesssim \left\| \eta (W\zeta _h-\mu ) \right\| _{H^{-1-\alpha _N}(\varGamma )} \nonumber \\\lesssim & {} h^{1+\alpha _N} \left( \left\| W\zeta _h \right\| _{L^2(\varGamma )}+\left| \mu \right| \right) . \end{aligned}$$

The last inequality follows from the orthogonality of $W\zeta _h$ to discrete functions in $S^{1,1}(\mathcal {T}_h)$ on $B''$ and the arguments shown in (4.47) below (specifically: go through the arguments of (4.47) with $z \equiv \mu$).

*Step 2 (Approximation of the near field):*

Let ${\mathcal {J}}_h$ denote the Scott–Zhang projection. The ellipticity of *W* on $H^{1/2}(\varGamma )/{\mathbb {R}}$ and the orthogonality (4.33) of $W\zeta _h = -\partial _n {\widetilde{K}}\zeta _h$ imply

4.47 $$\begin{aligned}&\left\| v_h \right\| _{H^{1/2}(\varGamma )} \lesssim \left\| \eta W\zeta _h - \eta z \right\| _{H^{-1/2}(\varGamma )} = \sup _{w\in H^{1/2}(\varGamma )} \frac{\left\langle \eta W\zeta _h - \eta z,w \right\rangle }{\left\| w \right\| _{H^{1/2}(\varGamma )}}\nonumber \\&= \sup _{w\in H^{1/2}(\varGamma )} \frac{\left\langle W\zeta _h,\eta w- {\mathcal {J}}_h(\eta w) \right\rangle -\left\langle \eta z,w \right\rangle +\mu \left\langle {\mathcal {J}}_h(\eta w),1 \right\rangle }{\left\| w \right\| _{H^{1/2}(\varGamma )}} \nonumber \\&= \sup _{w\in H^{1/2}(\varGamma )} \frac{\left\langle W\zeta _h,\eta w- {\mathcal {J}}_h(\eta w) \right\rangle -\left\langle \eta (z-\mu ),w \right\rangle -\mu \left\langle \eta w- {\mathcal {J}}_h(\eta w),1 \right\rangle }{\left\| w \right\| _{H^{1/2}(\varGamma )}} \nonumber \\&\lesssim \sup _{w\in H^{1/2}(\varGamma )} \frac{\left( \left\| W\zeta _h \right\| _{L^2(\varGamma )}+\left| \mu \right| \right) \left\| \eta w- {\mathcal {J}}_h(\eta w) \right\| _{L^2(\varGamma )}+ \left\| \eta (z-\mu ) \right\| _{H^{-1/2}(\varGamma )}\left\| w \right\| _{H^{1/2}(\varGamma )}}{\left\| w \right\| _{H^{1/2}(\varGamma )}} \nonumber \\&\lesssim h^{1/2}\left( \left\| W\zeta _h \right\| _{L^2(\varGamma )}+\left| \mu \right| \right) + \left\| \eta (z-\mu ) \right\| _{H^{-1/2}(\varGamma )} \nonumber \\&{\mathop {\lesssim }\limits ^{(4.46)}} h^{1/2}\left( \left\| W\zeta _h \right\| _{L^2(\varGamma )}+\left| \mu \right| \right) . \end{aligned}$$

With the same arguments and Lemma 4.9 we may estimate

4.48 $$\begin{aligned} \left\| \eta W\zeta _h - \eta z \right\| _{H^{-1-\alpha _N}(\varGamma )} \lesssim h^{1+\alpha _N}\left( \left\| W\zeta _h \right\| _{L^2(\varGamma )} + \left| \mu \right| \right) + \left\| \eta (z-\mu ) \right\| _{H^{-1-\alpha _N}(\varGamma )}.\nonumber \\ \end{aligned}$$

Let $\psi$ solve $W\psi = w-\overline{w}$ for $w \in H^{\alpha _N}(\varGamma )$. Then $\psi \in H^{1+\alpha _N}(\varGamma )$. Together with the mapping properties of *W* from Lemma 3.5, $\left\langle v_h,1 \right\rangle = 0$, the definition of $v_h$, and the stability and approximation properties of ${\mathcal {J}}_h$ from Lemma 4.9, we obtain

4.49 $$\begin{aligned}&\left\| Wv_h \right\| _{H^{-1-\alpha _N}(\varGamma )} \lesssim \left\| v_h \right\| _{H^{-\alpha _N}(\varGamma )} = \sup _{w\in H^{\alpha _N}(\varGamma )}\frac{\left\langle v_h,w \right\rangle }{\left\| w \right\| _{H^{\alpha _N}(\varGamma )}} = \sup _{w\in H^{\alpha _N}(\varGamma )}\frac{\left\langle v_h,w-\overline{w} \right\rangle }{\left\| w \right\| _{H^{\alpha _N}(\varGamma )}} \nonumber \\&\qquad \le \sup _{\psi \in H^{1+\alpha _N}(\varGamma )}\frac{\left| \left\langle v_h,W\psi \right\rangle \right| }{\left\| \psi \right\| _{H^{1+\alpha _N}(\varGamma )}} =\sup _{\psi \in H^{1+\alpha _N}(\varGamma )}\frac{\left| \left\langle Wv_h,\psi -{\mathcal {J}}_h \psi \right\rangle + \left\langle Wv_h,{\mathcal {J}}_h\psi \right\rangle \right| }{\left\| \psi \right\| _{H^{1+\alpha _N}(\varGamma )}} \nonumber \\&\qquad =\sup _{\psi \in H^{1+\alpha _N}(\varGamma )}\frac{\left| \left\langle Wv_h,\psi -{\mathcal {J}}_h \psi \right\rangle + \left\langle \eta (W\zeta _h-z),{\mathcal {J}}_h\psi \right\rangle \right| }{\left\| \psi \right\| _{H^{1+\alpha _N}(\varGamma )}} \nonumber \\&\qquad \lesssim \sup _{\psi \in H^{1+\alpha _N}(\varGamma )}\frac{\left\| Wv_h \right\| _{H^{-1/2}(\varGamma )}\left\| \psi -{\mathcal {J}}_h \psi \right\| _{H^{1/2}(\varGamma )} + \left\| \eta (W\zeta _h-z) \right\| _{H^{-1-\alpha _D}(\varGamma )}\left\| {\mathcal {J}}_h\psi \right\| _{H^{1+\alpha _D}(\varGamma )}}{\left\| \psi \right\| _{H^{1+\alpha _N}(\varGamma )}} \nonumber \\&\qquad {\mathop {\lesssim }\limits ^{(4.47), (4.48),(4.46)}} h^{1+\alpha _N}\left( \left\| W\zeta _h \right\| _{L^2(\varGamma )} + \left| \mu \right| \right) . \end{aligned}$$

With the mapping properties of *W* from Lemma 3.5, an inverse estimate, and (4.47) we obtain for $0\le \varepsilon \le \alpha _N$

4.50 $$\begin{aligned} \left\| Wv_h \right\| _{H^{\varepsilon }(\varGamma )}&\lesssim \left\| v_h \right\| _{H^{1+\varepsilon }(\varGamma )}\lesssim h^{-\varepsilon -1/2}\left\| v_h \right\| _{H^{1/2}(\varGamma )}\nonumber \\&{\mathop {\lesssim }\limits ^{(4.47)}} h^{-\varepsilon }\left( \left\| W\zeta _h \right\| _{L^2(\varGamma )}+\left| \mu \right| \right) \lesssim h^{-\varepsilon }\left( \left\| \zeta _h \right\| _{H^1(\varGamma )}+\left| \mu \right| \right) . \end{aligned}$$

We first consider $\gamma _0^\mathrm{int} u_\mathrm{near}$; the case $\gamma _0^\mathrm{ext} u_\mathrm{near}$ is treated analogously. By construction of $u_\mathrm{near}$, we have4.51since $z\equiv \mu$, $\eta \equiv 1$ on . Therefore, $u_{\text {near}} \in {\mathcal H}^{{\mathcal {N}}}_h(B',0)$.

Let ${\widehat{\eta }}$ be another cut-off function satisfying ${\widehat{\eta }} \equiv 1$ on ${\widehat{\varGamma }}$ and . The multiplicative trace inequality, see, e.g., [16, Thm. A.2], implies for any $\varepsilon \le 1/2$ that

4.52 $$\begin{aligned} \left| \gamma _0^\mathrm{int}u_{\text {near}} \right| _{H^1({\widehat{\varGamma }})}\lesssim & {} \left\| \nabla ({\widehat{\eta }} u_{\text {near}}) \right\| _{L^2(B\cap \varGamma )} \lesssim \left\| \nabla ({\widehat{\eta }} u_{\text {near}}) \right\| _{L^2(\varOmega )}^{2\varepsilon /(1+2\varepsilon )} \left\| \nabla ({\widehat{\eta }} u_{\text {near}}) \right\| _{H^{1/2+\varepsilon } (\varOmega )}^{1/(1+2\varepsilon )}\nonumber \\\lesssim & {} \left\| \nabla (\widehat{\eta }u_{\text {near}}) \right\| _{L^2(B)}^{2\varepsilon /(1+2\varepsilon )} \left\| {\widehat{\eta }}u_{\text {near}} \right\| _{H^{3/2+\varepsilon }(B)}^{1/ (1+2\varepsilon )}. \end{aligned}$$

Since $u_{\text {near}} \in {\mathcal H}^{{\mathcal {N}}}_h(B',0)$, we may use the interior regularity estimate (4.35) with $\mu = 0$ for the first term on the right-hand side of (4.52). The second factor of (4.52) can be estimated using (3.16) of Lemma 3.4. In total, we get for $\varepsilon \le \alpha _N<1/2$ that

4.53 $$\begin{aligned}&\left\| \nabla ({\widehat{\eta }}u_{\text {near}}) \right\| _{L^2(B)}^{2\varepsilon / (1+2\varepsilon )} \left\| {\widehat{\eta }}u_{\text {near}} \right\| _{H^{3/2+\varepsilon }(B)}^{1/ (1+2\varepsilon )}\nonumber \\&\quad \lesssim \left( h\left\| \nabla u_{\text {near}} \right\| _{L^2(B')} + \left\| u_{\text {near}} \right\| _{L^2(B')}\right) ^{2\varepsilon /(1+2\varepsilon )} \cdot \left( \left\| u_{\text {near}} \right\| _{H^1(B')} +\left\| \partial _n u_{\text {near}} \right\| _{H^{\varepsilon }(\varGamma )} \right) ^{1/(1+2\varepsilon )} \nonumber \\&\quad \lesssim h^{2\varepsilon /(1+2\varepsilon )} \left\| u_{\text {near}} \right\| _{H^1(B')} + \left\| u_{\text {near}} \right\| _{L^2(B')}^{2\varepsilon / (1+2\varepsilon )}\left\| u_{\text {near}} \right\| _{H^1(B')}^{1/(1+2\varepsilon )} \nonumber \\&\quad + \left\| u_{\text {near}} \right\| _{L^2(B')}^{2\varepsilon /(1+2\varepsilon )} \left\| Wv_h \right\| _{H^{\varepsilon }(\varGamma )}^{1/(1+2\varepsilon )} + h^{2\varepsilon /(1+2\varepsilon )}\left\| \nabla u_{\text {near}} \right\| _{L^2(B')}^{2\varepsilon /(1+2\varepsilon )} \left\| W v_h \right\| _{H^{\varepsilon }(\varGamma )}^{1/(1+2\varepsilon )} \nonumber \\&\quad =: T_1 + T_2 + T_3 + T_4. \end{aligned}$$

The mapping properties of ${\widetilde{K}}$ imply with (4.47) and (4.50)

4.54 $$\begin{aligned}&T_1 = h^{2\varepsilon /(1+2\varepsilon )} \left\| u_{\text {near}} \right\| _{H^1(B')}\lesssim h^{2\varepsilon /(1+2\varepsilon )} \left\| v_h \right\| _{H^{1/2}(\varGamma )}\nonumber \\&\,{\mathop {\lesssim }\limits ^{(4.47)}} h^{2\varepsilon /(1+2\varepsilon )+1/2}\left( \left\| \zeta _h \right\| _{H^{1}(\varGamma )} +\left| \mu \right| \right) ,\nonumber \\&T_4 = h^{2\varepsilon /(1+2\varepsilon )}\left\| \nabla u_{\text {near}} \right\| _{L^2(B')}^{2\varepsilon /(1+2\varepsilon )} \left\| Wv_h \right\| _{H^{\varepsilon }(\varGamma )}^{1/(1+2\varepsilon )}\nonumber \\&\,{\mathop {\lesssim }\limits ^{(4.50)}} h^{2\varepsilon /(1+2\varepsilon )}\left( \left\| \zeta _h \right\| _{H^{1}(\varGamma )} +\left| \mu \right| \right) . \end{aligned}$$

We apply (3.15) (note: $u_{\text {near}}$ has mean zero) and since ${\widetilde{K}} v_h$ is smooth on $\partial B_{R_{\varOmega }}(0)$, we can estimate $\left\| {\widetilde{K}}v_h \right\| _{H^{-\alpha _N}(\partial B_{R_{\varOmega }}(0))} \lesssim \left\| v_h \right\| _{H^{-\alpha _N}(\varGamma )}$. Together with (4.50), (4.49), and Young’s inequality this leads to

$$\begin{aligned} T_3&= \left\| u_{\text {near}} \right\| _{L^2(B')}^{2\varepsilon /(1+2\varepsilon )} \left\| Wv_h \right\| _{H^{\varepsilon }(\varGamma )}^{1/(1+2\varepsilon )} \\&{\mathop {\lesssim }\limits ^{(3.15), (4.50)}} h^{-\varepsilon /(1+2\varepsilon )} \left( \left\| Wv_h \right\| _{H^{-1-\alpha _N}(\varGamma )} +\left\| {\widetilde{K}}v_h \right\| _{H^{-\alpha _N}(\partial B_{R_{\varOmega }}(0))}\right) ^{2\varepsilon /(1+2\varepsilon )}\\&\qquad \qquad \qquad \qquad \cdot \left( \left\| \zeta _h \right\| _{H^{1}(\varGamma )} +\left| \mu \right| \right) ^{1/(1+2\varepsilon )} \\&\lesssim h^{-1}\left( \left\| Wv_h \right\| _{H^{-1-\alpha _N}(\varGamma )} + \left\| v_h \right\| _{H^{-\alpha _N}(\varGamma )}\right) + h^{\varepsilon }\left( \left\| \zeta _h \right\| _{H^{1}(\varGamma )} +\left| \mu \right| \right) \\&{\mathop {\lesssim }\limits ^{(4.49)}} \left( h^{\alpha _N} + h^{\varepsilon } \right) \left( \left\| \zeta _h \right\| _{H^{1}(\varGamma )} +\left| \mu \right| \right) . \end{aligned}$$

Similarly, with (4.54) we get for the second term in (4.53)

$$\begin{aligned} T_2&= \left\| u_{\text {near}} \right\| _{L^2(B')}^{2\varepsilon /(1+2\varepsilon )} \left\| u_{\text {near}} \right\| _{H^1(B')}^{1/(1+2\varepsilon )} \\ {}&{\mathop {\lesssim }\limits ^{(3.15)}} h^{-\varepsilon /(1+2\varepsilon )} \left( \left\| Wv_h \right\| _{H^{-1-\alpha _N}(\varGamma )} +\left\| {\widetilde{K}}v_h \right\| _{H^{-\alpha _N}(\partial B_{R_{\varOmega }}(0))}\right) ^{2\varepsilon /(1+2\varepsilon )} \\&\qquad \qquad \cdot h^{(\varepsilon +1/2)/(1+2\varepsilon )} \left( \left\| \zeta _h \right\| _{H^{1}(\varGamma )}+\left| \mu \right| \right) ^{1/(1+2\varepsilon )} \\&\lesssim h^{-1/2}\left( \left\| Wv_h \right\| _{H^{-1-\alpha _N}(\varGamma )}+ \left\| v_h \right\| _{H^{-\alpha _N}(\varGamma )} \right) + h^{1/2+\varepsilon }\left( \left\| \zeta _h \right\| _{H^{1}(\varGamma )} +\left| \mu \right| \right) \\ {}&\lesssim \left( h^{1/2+\alpha _N} + h^{1/2+\varepsilon } \right) \left( \left\| \zeta _h \right\| _{H^{1}(\varGamma )} +\left| \mu \right| \right) . \end{aligned}$$

Inserting everything in (4.53) and choosing $\varepsilon = \alpha _N$ gives

$$\begin{aligned} \left| \gamma _0^\mathrm{int} u_{\text {near}} \right| _{H^1({\widehat{\varGamma }})}\lesssim & {} (h^{2\alpha _N/(1+2\alpha _N)+1/2} + h^{1/2+\alpha _N} +h^{\alpha _N}\\&+\,h^{2\alpha _N/(1+2\alpha _N)})\left( \left\| \zeta _h \right\| _{H^1(\varGamma )}+\left| \mu \right| \right) \\\lesssim & {} h^{\alpha _N}\left( \left\| \zeta _h \right\| _{H^1(\varGamma )}+\left| \mu \right| \right) . \end{aligned}$$

Applying the same argument for the exterior trace leads to an estimate for the jump of the trace

$$\begin{aligned} \left| [\gamma _0 u_{\text {near}}] \right| _{H^1({\widehat{\varGamma }})} \lesssim h^{\alpha _N}\left( \left\| \zeta _h \right\| _{H^1(\varGamma )}+\left| \mu \right| \right) . \end{aligned}$$

*Step 3 (Approximation of the far field):*

We define the function $\nu \in H^{1/2}(\varGamma )$ as the solution of

$$\begin{aligned} W\nu = (1-\eta ) W\zeta _h + \eta z, \qquad \left\langle \nu ,1 \right\rangle =0. \end{aligned}$$

Then, we have

$$\begin{aligned} \left\langle W(\nu -\nu _h),\psi _h \right\rangle = 0 \qquad \forall \psi _h \in S^{1,1}(\mathcal {T}_h). \end{aligned}$$

Let $\widehat{u}_\mathrm{far} {:}{=} {\widetilde{K}}\nu -\overline{{\widetilde{K}}\nu }$ where $\overline{{\widetilde{K}}\nu }{:}{=}\frac{1}{\left| \varOmega \right| }\left\langle {\widetilde{K}}\nu ,1 \right\rangle _{L^2(\varOmega )}$ and ${\widehat{\eta }}$ be another cut-off function with ${\widehat{\eta }} \equiv 1$ on ${\widehat{\varGamma }}$ and . Then, with the Galerkin projection $\varPi _W$, the triangle inequality and the jump conditions of ${\widetilde{K}}$ imply

4.55 $$\begin{aligned} \left| [\gamma _0 u_{\text {far}}] \right| _{H^1({\widehat{\varGamma }})} = \left| {\widehat{\eta }}\nu _h \right| _{H^1({\widehat{\varGamma }})} \le \left| {\widehat{\eta }}\nu _h-\varPi _W(\widehat{\eta }\nu ) \right| _{H^1({\widehat{\varGamma }})} + \left| \varPi _W(\widehat{\eta }\nu ) \right| _{H^1({\widehat{\varGamma }})}.\qquad \end{aligned}$$

The smoothness of ${\widetilde{K}}\nu$ on $\partial B_{R_{\varOmega }}(0)$ and the coercivity of *W* on $H^{1/2}(\varGamma )/{\mathbb {R}}$ lead to

$$\begin{aligned} \left\| {\widetilde{K}}\nu -\overline{{\widetilde{K}}\nu } \right\| _{H^{1/2}(\partial B_{R_{\varOmega }}(0))} \lesssim \left\| \nu \right\| _{H^{1/2}(\varGamma )} \lesssim \left\| W\nu \right\| _{H^{-1/2}(\varGamma )}. \end{aligned}$$

We apply Lemma 3.4 with a cut-off function ${\widetilde{\eta }}$ satisfying ${\widetilde{\eta }} \equiv 1$ on $B\cap \varGamma$ and . Then $\eta \equiv 1$ and $z \equiv \mu$ on $B'\cap \varGamma$ imply ${\widetilde{\eta }}(1-\eta )\equiv 0$ and ${\widetilde{\eta }} \eta z = {\widetilde{\eta }} \mu$. The $H^1$-stability of the Galerkin projection from Lemma 4.8, a facewise trace estimate, and similar estimates as for the near field imply

4.56 $$\begin{aligned} \left| \varPi _W(\widehat{\eta }\nu ) \right| _{H^1({\widehat{\varGamma }})}\lesssim & {} \left| \widehat{\eta }\nu \right| _{H^1(\varGamma )} \lesssim \left\| \widehat{u}_{\text {far}} \right\| _{H^{3/2+\alpha _N}(B\backslash \varGamma )}\nonumber \\&{\mathop {\lesssim }\limits ^{(3.16)}} \left\| \widehat{u}_{\text {far}} \right\| _{H^{1}(B'\backslash \varGamma )}+ \left\| {\widetilde{\eta }}((1-\eta )W\zeta _h + \eta z) \right\| _{H^{\alpha _N}(\varGamma )}\nonumber \\&\lesssim \left\| \widehat{u}_{\text {far}} \right\| _{H^{1}(B'\backslash \varGamma )} + \left| \mu \right| \left\| {\widetilde{\eta }} \right\| _{H^{\alpha _N}(\varGamma )}\nonumber \\&\lesssim \left\| (1-\eta )W\zeta _h + \eta z \right\| _{H^{-1/2}(\varGamma )}+ \left\| {\widetilde{K}}\nu -\overline{{\widetilde{K}}\nu } \right\| _{H^{1/2}(\partial B_{R_{\varOmega }}(0))}+\left| \mu \right| \nonumber \\&\lesssim \left\| (1-\eta )W\zeta _h + \eta z \right\| _{H^{-1/2}(\varGamma )}+\left| \mu \right| \nonumber \\&\lesssim \left\| \zeta _h \right\| _{H^{1/2}(\varGamma )}+\left\| \eta (z -\mu ) \right\| _{H^{-1/2}(\varGamma )} + \left| \mu \right| \nonumber \\&{\mathop {\lesssim }\limits ^{(4.46)}} \left\| \zeta _h \right\| _{H^{1/2}(\varGamma )}+ \left| \mu \right| . \end{aligned}$$

It remains to estimate the first term on the right-hand side of (4.55). With an inverse estimate and Lemma 4.8 we get

4.57 $$\begin{aligned}&\left| {\widehat{\eta }}\nu _h-\varPi _W(\widehat{\eta }\nu ) \right| _{H^1({\widehat{\varGamma }})} \lesssim \left| {\widehat{\eta }}\nu _h-\varPi _W(\widehat{\eta }\nu _h ) \right| _{H^{1}({\widehat{\varGamma }})} + h^{-1/2}\left| \varPi _W({\widehat{\eta }}\nu _h-\widehat{\eta }\nu ) \right| _{H^{1/2}(\varGamma )} \nonumber \\&\qquad \qquad \qquad \lesssim h\left| \nu _h \right| _{H^{1}(\varGamma )} + h^{-1/2}\left| \varPi _W({\widehat{\eta }}\nu _h-\widehat{\eta }\nu ) \right| _{H^{1/2}(\varGamma )}\nonumber \\&\qquad \qquad \qquad \lesssim h^{1/2}\left\| \nu _h \right\| _{H^{1/2}(\varGamma )} + h^{-1/2}\left| \varPi _W({\widehat{\eta }}\nu _h-\widehat{\eta }\nu ) \right| _{H^{1/2}(\varGamma )}. \end{aligned}$$

We use the abbreviation $e_{\nu } {:}{=} \nu - \nu _h$. The ellipticity of *W* on $H^{1/2}(\varGamma )/{\mathbb {R}}$ and the definition of the Galerkin projection $\varPi _W$ imply

$$\begin{aligned}&\left\| \varPi _W(\widehat{\eta }e_{\nu } ) \right\| _{H^{1/2}(\varGamma )}^2 \lesssim \left\langle W(\varPi _W(\widehat{\eta }e_{\nu } )),\varPi _W(\widehat{\eta }e_{\nu }) \right\rangle + \left| \left\langle \varPi _W(\widehat{\eta }e_{\nu } ),1 \right\rangle \right| ^2 \nonumber \\&\qquad = \left\langle W(\varPi _W(\widehat{\eta }e_{\nu } )-\widehat{\eta }e_{\nu }),\varPi _W(\widehat{\eta }e_{\nu }) \right\rangle + \left\langle W(\widehat{\eta }e_{\nu }),\varPi _W(\widehat{\eta }e_{\nu }) \right\rangle + \left| \left\langle \varPi _W(\widehat{\eta }e_{\nu } ),1 \right\rangle \right| ^2\nonumber \\&\quad {\mathop {=}\limits ^{(4.36)}} \left\langle W(\widehat{\eta }e_{\nu }),\varPi _W(\widehat{\eta }e_{\nu }) \right\rangle + \left\langle \widehat{\eta }e_{\nu } ,1 \right\rangle \left\langle \varPi _W(\widehat{\eta }e_{\nu } ),1 \right\rangle \nonumber \\&\qquad \lesssim \left| \left\langle W(\widehat{\eta }e_{\nu }),\varPi _W(\widehat{\eta }e_{\nu }) \right\rangle \right| + \left\| \widehat{\eta }e_{\nu } \right\| _{H^{-1/2}(\varGamma )}\left\| \varPi _W(\widehat{\eta }e_{\nu } ) \right\| _{H^{1/2}(\varGamma )}. \end{aligned}$$

With the commutator ${\mathcal {C}}_{\widehat{\eta }}$ we get

4.58 $$\begin{aligned} \left\langle W(\widehat{\eta }e_{\nu }),\varPi _W(\widehat{\eta }e_{\nu }) \right\rangle = \left\langle \widehat{\eta }W(e_{\nu })+{\mathcal {C}}_{{\widehat{\eta }}}e_{\nu },\varPi _W(\widehat{\eta }e_{\nu }) \right\rangle . \end{aligned}$$

The definition of the Galerkin projection and the super-approximation properties of the Scott-Zhang projection ${\mathcal {J}}_h$ lead to

$$\begin{aligned} \left\langle W(e_{\nu }),\widehat{\eta }\varPi _W(\widehat{\eta }e_{\nu }) \right\rangle= & {} \left\langle W(e_{\nu }),\widehat{\eta }\varPi _W(\widehat{\eta }e_{\nu }) -{\mathcal {J}}_h(\widehat{\eta }\varPi _W(\widehat{\eta }e_{\nu })) \right\rangle \\\lesssim & {} \left\| W(e_{\nu }) \right\| _{H^{-1/2}(\varGamma )} \left\| \widehat{\eta }\varPi _W(\widehat{\eta }e_{\nu }) -{\mathcal {J}}_h(\widehat{\eta }\varPi _W(\widehat{\eta }e_{\nu })) \right\| _{H^{1/2}(\varGamma )} \\\lesssim & {} h\left\| \nu -\nu _h \right\| _{H^{1/2}(\varGamma )}\left\| \varPi _W(\widehat{\eta }e_{\nu }) \right\| _{H^{1/2}(\varGamma )}. \end{aligned}$$

For the term involving $\mathcal {C}_{\widehat{\eta }}$ in (4.58), we get with Lemma 3.6

$$\begin{aligned} \left| \left\langle {\mathcal {C}}_{{\widehat{\eta }}}(e_{\nu }),\varPi _W(\widehat{\eta }e_{\nu } ) \right\rangle \right|\lesssim & {} \left\| {\mathcal {C}}_{{\widehat{\eta }}}(\nu -\nu _h ) \right\| _{H^{-1/2}(\varGamma )} \left\| \varPi _W(\widehat{\eta }e_{\nu } ) \right\| _{H^{1/2}(\varGamma )} \\\lesssim & {} \left\| \nu -\nu _h \right\| _{H^{-\alpha _N}(\varGamma )} \left\| \varPi _W(\widehat{\eta }e_{\nu }) \right\| _{H^{1/2}(\varGamma )}. \end{aligned}$$

A duality argument implies $\left\| e_{\nu } \right\| _{H^{-\alpha _N}(\varGamma )}\lesssim h^{1/2+\alpha _N}\left\| \nu \right\| _{H^{1/2}(\varGamma )}$, for details we refer to the proof of Corollary 2.9. Inserting everything in (4.57) leads to

$$\begin{aligned} \left| {\widehat{\eta }}\nu _h-\varPi _W(\widehat{\eta }\nu ) \right| _{H^1({\widehat{\varGamma }})}\lesssim & {} h^{1/2}\left\| \nu _h \right\| _{H^{1/2}(\varGamma )} + h^{1/2}\left\| \nu -\nu _h \right\| _{H^{1/2}(\varGamma )} + h^{\alpha _N}\left\| \nu \right\| _{H^{1/2}(\varGamma )} \\\lesssim & {} h^{\alpha _N} \left\| (1-\eta )W\zeta _h + \eta z \right\| _{H^{-1/2}(\varGamma )} \lesssim h^{\alpha _N}\left( \left\| \zeta _h \right\| _{H^{1/2}(\varGamma )} + \left| \mu \right| \right) . \end{aligned}$$

Finally, this implies with (4.55) and (4.56) that

$$\begin{aligned} \left| [\gamma _0 u_{\text {far}}] \right| _{H^1({\widehat{\varGamma }})} \lesssim (1+h^{\alpha _N})\left( \left\| \zeta _h \right\| _{H^{1/2}(\varGamma )} + \left| \mu \right| \right) , \end{aligned}$$

which proves the lemma. $\square$

#### Lemma 4.11

Let $\varphi ,\varphi _h$ be solutions of (2.8), (2.9) and let $\varGamma _0, {\widehat{\varGamma }}$ be subsets of $\varGamma$ with $\varGamma _0\subset {\widehat{\varGamma }} \subsetneq \varGamma$ and . Let *h* be such that $\frac{h}{R}\le \frac{1}{12}$, and let $\eta \in C_0^{\infty }({\mathbb {R}}^d)$ satisfy $\eta \equiv 1$ on $\varGamma _0$, . Then, we have

$$\begin{aligned} \left\| \varphi -\varphi _h \right\| _{H^{1}(\varGamma _0)}\le & {} C \Big ( \inf _{\chi _h\in S^{1,1}(\mathcal {T}_h)} \left\| \varphi - \chi _h \right\| _{H^{1}({\widehat{\varGamma }})} + h^{\alpha _N}\left| \varphi -\varphi _h \right| _{H^{1}({\widehat{\varGamma }})} + \\&+ \left\| \eta (\varphi -\varphi _h) \right\| _{H^{1/2}(\varGamma )}+ \left\| \varphi -\varphi _h \right\| _{H^{-\alpha _N}(\varGamma )}\Big ) \end{aligned}$$

with a constant $C>0$ depending only on $\varGamma ,\varGamma _0,{\widehat{\varGamma }},d,R$, and the $\gamma$-shape regularity of $\mathcal {T}_h$.

#### Proof:

We define $e{:}{=} \varphi -\varphi _h$, subsets $\varGamma _0\subset \varGamma _1\subset \varGamma _2 \subset \varGamma _3\subset \varGamma _4 \subset {\widehat{\varGamma }}$, and volume boxes $B_0 \subset B_1 \subset B_2 \subset B_3 \subset B_4\subset {\mathbb {R}}^d$, where $B_i\cap \widehat{\varGamma } = \varGamma _i$. Throughout the proof, we use cut-off functions $\eta _i \in C_0^{\infty }({\mathbb {R}}^d)$, $i=1,\dots ,4$. These smooth functions $\eta _i$ should satisfy $\eta _i \equiv 1$ on $\varGamma _{i-1}$, .

We want to use Lemma 4.10. Since $[\gamma _0{\widetilde{K}}\zeta _h] = \zeta _h \in S^{1,1}(\mathcal {T}_h)$ for any discrete function $\zeta _h \in S^{1,1}(\mathcal {T}_h)$, we need to construct a discrete function satisfying the orthogonality (4.33). Using the Galerkin orthogonality with test functions with  and noting that $\eta _3 \equiv 1$ on , we obtain with the commutator ${\mathcal {C}}_{\eta _3}$ defined in (3.18), the abbreviation $\overline{\eta _3{\mathcal {C}}_{\eta _3}e}=\frac{1}{\left| \varGamma \right| }\left\langle \eta _3 {\mathcal {C}}_{\eta _3}e,1 \right\rangle$, and the Galerkin projection $\varPi _W$ from (4.36)

4.59 $$\begin{aligned} 0= & {} \left\langle We,\eta _3\psi _h \right\rangle + \left\langle e,1 \right\rangle \left\langle \psi _h,1 \right\rangle = \left\langle \eta _3We,\psi _h \right\rangle + \left\langle e,1 \right\rangle \left\langle \psi _h,1 \right\rangle \nonumber \\= & {} \left\langle W(\eta _3e)-{\mathcal {C}}_{\eta _3}e,\psi _h \right\rangle + \left\langle e,1 \right\rangle \left\langle \psi _h,1 \right\rangle \nonumber \\= & {} \left\langle W(\eta _3e)-(\eta _3{\mathcal {C}}_{\eta _3}e-\overline{\eta _3{\mathcal {C}}_{\eta _3}e}),\psi _h \right\rangle - \left\langle \overline{\eta _3{\mathcal {C}}_{\eta _3}e},\psi _h \right\rangle +\left\langle e,1 \right\rangle \left\langle \psi _h,1 \right\rangle \nonumber \\= & {} \left\langle W(\eta _3e-W^{-1}(\eta _3{\mathcal {C}}_{\eta _3}e-\overline{\eta _3{\mathcal {C}}_{\eta _3}e})),\psi _h \right\rangle \nonumber \\&- \frac{1}{\left| \varGamma \right| }\left\langle \eta _3 {\mathcal {C}}_{\eta _3}e,1 \right\rangle \left\langle \psi _h,1 \right\rangle +\left\langle e,1 \right\rangle \left\langle \psi _h,1 \right\rangle \nonumber \\= & {} \left\langle W(\varPi _W(\eta _3e)-\varPi _W(W^{-1}(\eta _3{\mathcal {C}}_{\eta _3}e-\overline{\eta _3 {\mathcal {C}}_{\eta _3}e}))),\psi _h \right\rangle \nonumber \\&- \frac{1}{\left| \varGamma \right| }\left\langle \eta _3 {\mathcal {C}}_{\eta _3}e,1 \right\rangle \left\langle \psi _h,1 \right\rangle + \left\langle e,1 \right\rangle \left\langle \psi _h,1 \right\rangle -\left\langle \eta _3e-\varPi _W(\eta _3e),1 \right\rangle \left\langle \psi _h,1 \right\rangle .\nonumber \\ \end{aligned}$$

Here and below, we understand the inverse $W^{-1}$ as the inverse of the bijective operator $W:H^{1/2}_{*}(\varGamma ){:}{=}\{v\in H^{1/2}(\varGamma ):\left\langle v,1 \right\rangle = 0\}\rightarrow H_{*}^{-1/2}(\varGamma ){:}{=}\{v\in H^{-1/2}(\varGamma ):\left\langle v,1 \right\rangle = 0\}$. Since $W^{-1}$ maps into $H_{*}^{1/2}(\varGamma )$ no additional terms in the orthogonality (4.59) appear. Thus, defining

$$\begin{aligned} \zeta _h {:}{=} \varPi _W(\eta _3e) - \xi _h \quad \text {with} \; \xi _h{:}{=}\varPi _W(W^{-1}(\eta _3{\mathcal {C}}_{\eta _3}e-\overline{\eta _3{\mathcal {C}}_{\eta _3}e})), \end{aligned}$$

we get on a volume box $B_2\subset {\mathbb {R}}^d$ a discrete harmonic function

$$\begin{aligned} u{:}{=}{\widetilde{K}}\zeta _h \in {\mathcal H}^{{\mathcal {N}}}_{h}(B_2,\mu ), \end{aligned}$$

where $$\mu = \left\langle e,1 \right\rangle -\frac{1}{\left| \varGamma \right| }\left\langle \eta _3 {\mathcal {C}}_{\eta _3}e,1 \right\rangle -\left\langle \eta _3e -\varPi _W(\eta _3e),1 \right\rangle $$.

With the Galerkin projection $\varPi _W$ from (4.36) and $\eta _3 \equiv 1$ on , we write

4.60 $$\begin{aligned} \left\| e \right\| _{H^{1}(\varGamma _0)}\lesssim & {} \left\| \eta _1 e \right\| _{H^{1}(\varGamma )}\nonumber \\\lesssim & {} \left\| \eta _1(\eta _3 e - \varPi _W(\eta _3 e)) \right\| _{H^{1}(\varGamma )} +\left\| \eta _1\zeta _h \right\| _{H^{1}(\varGamma )} + \left\| \eta _1 \xi _h \right\| _{H^{1}(\varGamma )}.\qquad \qquad \end{aligned}$$

Lemma 4.8 leads to

4.61 $$\begin{aligned} \left\| \eta _3 e - \varPi _W(\eta _3 e) \right\| _{H^{1}(\varGamma )}\lesssim & {} h \left\| \eta _4\varphi _h \right\| _{H^{1}(\varGamma )} + \left\| \eta _4\varphi \right\| _{H^{1}(\varGamma )} \nonumber \\\lesssim & {} h \left\| \eta _4 e \right\| _{H^{1}(\varGamma )} + (h+1)\left\| \eta _4\varphi \right\| _{H^{1}(\varGamma )}. \end{aligned}$$

Using the $H^1$-stability of the Galerkin projection $\varPi _W$, the mapping properties of $W^{-1}$ and ${\mathcal {C}}_{\eta _3}$ as well as Lemma 3.6, the correction $\xi _h$ can be estimated by

4.62 $$\begin{aligned}&\left\| \varPi _W(W^{-1}(\eta _3{\mathcal {C}}_{\eta _3}e -\overline{\eta _3{\mathcal {C}}_{\eta _3}e})) \right\| _{H^{1}(\varGamma )}\nonumber \\&\quad \lesssim \left\| W^{-1}(\eta _3{\mathcal {C}}_{\eta _3}e -\overline{\eta _3{\mathcal {C}}_{\eta _3}e}) \right\| _{H^{1}(\varGamma )}\nonumber \\&\quad \lesssim \left\| \eta _3 {\mathcal {C}}_{\eta _3}e -\overline{\eta _3{\mathcal {C}}_{\eta _3}e} \right\| _{L^{2}(\varGamma )}\nonumber \\&\quad \lesssim \left\| \eta _3 {\mathcal {C}}_{\eta _3}e \right\| _{L^{2}(\varGamma )} \lesssim \left\| {\mathcal {C}}_{\eta _3}(\eta _3e) \right\| _{L^2(\varGamma )} + \left\| {\mathcal {C}}_{\eta _3}^{\eta _3}e \right\| _{L^2(\varGamma )} \nonumber \\&\quad \lesssim \left\| \eta _3e \right\| _{L^2(\varGamma )} + \left\| e \right\| _{H^{-\alpha _N}(\varGamma )}. \end{aligned}$$

For the second term on the right-hand side of (4.60) we have $\left\| \eta _1\zeta _h \right\| _{H^{1}(\varGamma )}\lesssim \left\| \eta _1\nabla \zeta _h \right\| _{L^{2}(\varGamma )} + \left\| \zeta _h \right\| _{L^{2}(\varGamma )}$. We apply Lemma 4.10 to $u = {\widetilde{K}}\zeta _h \in \mathcal {H}_h^{{\mathcal {N}}}(B_2,\mu )$ and obtain

4.63 $$\begin{aligned} \left\| \eta _1\nabla \zeta _h \right\| _{L^{2}(\varGamma )}\lesssim & {} \left| \zeta _h \right| _{H^{1}(\varGamma _1)} = \left| [\gamma _0 u] \right| _{H^{1}(\varGamma _1)} \nonumber \\\lesssim & {} h^{\alpha _N}\left| \zeta _h \right| _{H^1(\varGamma )} + \left\| \zeta _h \right\| _{H^{1/2}(\varGamma )} + \left| \mu \right| . \end{aligned}$$

The $H^1$-stability of the Galerkin-projection from Lemma 4.8 and (4.62) lead to

4.64 $$\begin{aligned} \left\| \zeta _h \right\| _{H^{1}(\varGamma )} \lesssim \left\| \eta _3e \right\| _{H^1(\varGamma )} + \left\| e \right\| _{H^{-\alpha _N}(\varGamma )} \end{aligned}$$

as well as

4.65 $$\begin{aligned} \left\| \zeta _h \right\| _{H^{1/2}(\varGamma )} \lesssim \left\| \eta _3e \right\| _{H^{1/2}(\varGamma )} + \left\| e \right\| _{H^{-\alpha _N}(\varGamma )}. \end{aligned}$$

With the estimate $\left| \left\langle e,1 \right\rangle \right| \lesssim \left\| e \right\| _{H^{-\alpha _N}(\varGamma )}$ and previous arguments (using (4.62), Lemma 4.8, and Lemma 3.6), we get

4.66 $$\begin{aligned} \left| \mu \right| \lesssim \left\| e \right\| _{H^{-\alpha _N}(\varGamma )} +\left\| \eta _3 e \right\| _{H^{1/2}(\varGamma )}+ \left\| \eta _3 e \right\| _{L^{2}(\varGamma )}. \end{aligned}$$

Inserting (4.64)–(4.66) in (4.63), we arrive at

4.67 $$\begin{aligned} \left\| \eta _1\zeta _h \right\| _{H^{1}(\varGamma )}&\lesssim \left\| \eta _1\nabla \zeta _h \right\| _{L^{2}(\varGamma )} + \left\| \zeta _h \right\| _{L^{2}(\varGamma )} \nonumber \\&\lesssim h^{\alpha _N} \left( \left\| \eta _3 e \right\| _{H^{1}(\varGamma )}+ \left\| e \right\| _{H^{-\alpha _N}(\varGamma )}\right) + \left\| \eta _3 e \right\| _{H^{1/2}(\varGamma )}+ \left\| e \right\| _{H^{-\alpha _N} (\varGamma )}\nonumber \\&\lesssim h^{\alpha _N}\left| e \right| _{H^{1}({\widehat{\varGamma }})}+ \left\| \eta _4 e \right\| _{H^{1/2}(\varGamma )}+ \left\| e \right\| _{H^{-\alpha _N}(\varGamma )}. \end{aligned}$$

Combining (4.61), (4.62), and (4.67) in (4.60), we finally obtain

$$\begin{aligned} \left\| e \right\| _{H^{1}(\varGamma _0)}\lesssim & {} h \left\| \eta _4 e \right\| _{H^{1}(\varGamma )} + \left\| \eta _4\varphi \right\| _{H^{1}(\varGamma )}+ h^{\alpha _N}\left| e \right| _{H^{1}({\widehat{\varGamma }})}\\&+\left\| \eta _4 e \right\| _{H^{1/2}(\varGamma )}+ \left\| e \right\| _{H^{-\alpha _N}(\varGamma )} \\\lesssim & {} \left\| \varphi \right\| _{H^{1}({\widehat{\varGamma }})}+ h^{\alpha _N}\left| e \right| _{H^{1}({\widehat{\varGamma }})}+\left\| \eta _4 e \right\| _{H^{1/2}(\varGamma )}+ \left\| e \right\| _{H^{-\alpha _N}(\varGamma )}. \end{aligned}$$

Since we only used the Galerkin orthogonality as a property of the error *e*, we may write $\varphi -\varphi _h = (\varphi -\chi _h)+(\chi _h - \varphi _h)$ for arbitrary $\chi _h \in S^{1,1}(\mathcal {T}_h)$ with  and we have proven the claimed inequality. $\square$

#### Proof

(*of Theorem* 2.8): Starting from Lemma 4.11, it remains to estimate the terms $h^{\alpha _N}\left| \varphi -\varphi _h \right| _{H^{1}({\widehat{\varGamma }})}$ and $\left\| \eta (\varphi -\varphi _h) \right\| _{H^{1/2}({\widehat{\varGamma }})}$. The terms are treated as in the proof of Theorem 2.3. Rather than using the operator $I_h\circ J_{ch}$ we may use the Scott-Zhang projection. $\square$

#### Proof

(*of Corollary* 2.9): The assumption $\varphi \in H^{1/2+\alpha }(\varGamma ) \cap H^{1+\beta }(\widetilde{\varGamma })$ leads to

$$\begin{aligned} \inf _{\chi _h \in S^{1,1}(\mathcal {T}_h)}\left\| \varphi -\chi _h \right\| _{H^{1}({\widehat{\varGamma }})}\lesssim & {} h^{\beta }\left\| \varphi \right\| _{H^{1+\beta }(\widetilde{\varGamma })}, \\ \left\| e \right\| _{H^{1/2}(\varGamma )}\lesssim & {} h^{\alpha }\left\| \varphi \right\| _{H^{1/2+\alpha }(\varGamma )}, \end{aligned}$$

where the second estimate is the standard global error estimate for the Galerkin BEM applied to the hyper-singular integral equation, see [22].

For the remaining term in Theorem 2.8, we use a duality argument. Let $\psi$ solve $W\psi = w- \overline{w} \in H^{\alpha _N}(\varGamma )$, $\left\langle \psi ,1 \right\rangle = 0$, where $\overline{w} = \frac{1}{\left| \varGamma \right| }\left\langle w,1 \right\rangle$. Then $\psi \in H^{1+\alpha _N}(\varGamma )$, and since $\left\langle e,1 \right\rangle = 0$, we get with the Scott-Zhang projection ${\mathcal {J}}_h$ and Lemma 4.9

$$\begin{aligned} \left\| e \right\| _{H^{-\alpha _N}(\varGamma )}&= \sup _{w\in H^{\alpha _N}(\varGamma )}\frac{\left\langle e,w \right\rangle }{\left\| w \right\| _{H^{\alpha _N}(\varGamma )}} = \sup _{w\in H^{\alpha _N}(\varGamma )}\frac{\left\langle e,w-\overline{w} \right\rangle }{\left\| w \right\| _{H^{\alpha _N}(\varGamma )}}\\&\lesssim \sup _{\psi \in H^{1+\alpha _N}(\varGamma )}\frac{\left| \left\langle e,W\psi \right\rangle \right| }{\left\| \psi \right\| _{H^{1+\alpha _N}(\varGamma )}} = \sup _{\psi \in H^{1+\alpha _N}(\varGamma )}\frac{\left| \left\langle We,\psi -{\mathcal {J}}_h\psi \right\rangle \right| }{\left\| \psi \right\| _{H^{1+\alpha _N}(\varGamma )}} \\&\lesssim \sup _{\psi \in H^{1+\alpha _N}(\varGamma )}\frac{\left\| We \right\| _{H^{-1/2}(\varGamma )} \left\| \psi -{\mathcal {J}}_h\psi \right\| _{H^{1/2}(\varGamma )}}{\left\| \psi \right\| _{H^{1+\alpha _N}(\varGamma )}} \lesssim h^{1/2+\alpha _N} \left\| e \right\| _{H^{1/2}(\varGamma )} \\&\lesssim h^{1/2+\alpha +\alpha _N} \left\| \varphi \right\| _{H^{1/2+\alpha }(\varGamma )}. \end{aligned}$$

Therefore, the term of slowest convergence is of order $\mathcal {O}(h^{\min \{1/2+\alpha +\alpha _N,\beta \}})$, which proves the corollary. $\square$

## Numerical examples

In this section we provide numerical examples to illustrate the theoretical results of Sect. 2 and indicate their sharpness. We only consider Symm’s integral equation on quasi-uniform meshes. Provided the right-hand side and the geometry are sufficiently smooth, it is well-known that the lowest order boundary element method in two dimensions converges in the energy norm as $O(N^{-3/2})$, where *N* denotes the degrees of freedom. In our examples we will consider problems, where the rate of convergence with uniform refinement is reduced due to singularities.

In order to compute the error between the exact solution and the Galerkin approximation, we prescribe the solution $u(r,\theta ) = r^{\alpha }\cos (\alpha \theta )$ of Laplace’s equation in polar coordinates. Then, the normal derivative $\phi = \partial _n u$ of *u* is the solution of

$$\begin{aligned} V\phi = (K+1/2)\gamma _0 u. \end{aligned}$$

The regularity of $\phi$ is determined by the choice of $\alpha$. In fact, we have $\phi \in H^{-1/2+\alpha -\varepsilon }(\varGamma )$, $\varepsilon >0$, and locally $\phi \in H^{1}(\widetilde{\varGamma })$ for all subsets $\widetilde{\varGamma } \subset \varGamma$ that are a positive distance away from the singularity at the origin.

The lowest order Galerkin approximation to $\phi$ is computed using the MATLAB-library HILBERT [2], where the errors in the $L^2$-norm are computed using two point Gauss-quadrature. The error in the local $H^{-1/2}$-norm is computed as $\left\| \chi e \right\| _{H^{-1/2}(\varGamma )}^2\sim \left\langle V(\chi e),\chi e \right\rangle$, where $\chi$ is the characteristic function for a union of elements $\varGamma _0\subset \varGamma$.

**Fig. 1:**
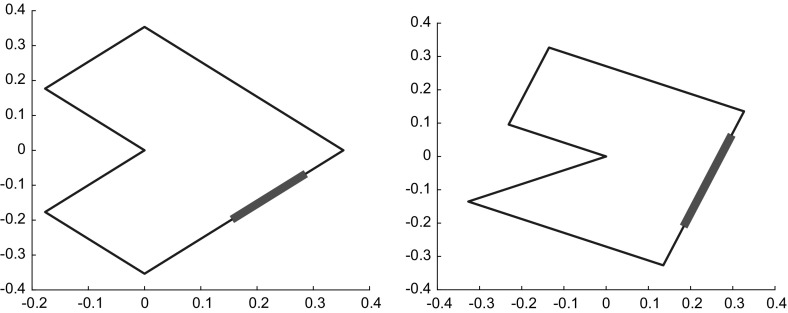
L-shaped and Z-shaped domain, local error computed on fat, red part

### Example 1: L-shaped domain

On the L-shaped domain depicted in Fig. 1 (left), the dual problem permits solutions of regularity $H^{1/6-\varepsilon }(\varGamma )$ for arbitrary $\varepsilon >0$; that is, we have $\alpha _D = \frac{1}{6}-\varepsilon$.

**Fig. 2:**
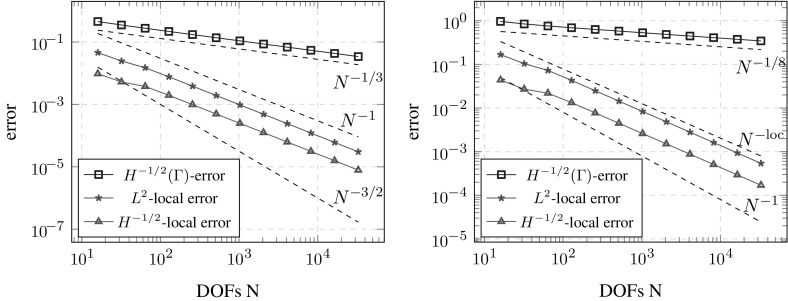
Local and global convergence of Galerkin-BEM for Symm’s equation, L-shaped domain, left: $\alpha = \frac{1}{3}$, right: $\alpha = \frac{1}{8}$, $\mathrm{loc} = \frac{19}{24}$

Figure 2 shows the global convergence in the energy norm (blue squares) as well as the local convergence on the fat, red part of the boundary ($\varGamma _0$, union of elements) in the $L^2$-norm (red stars) as well as the $H^{-1/2}$-norm (brown triangles). The black dotted lines mark the reference curves of order $N^{-\beta }$ for various $\beta > 0$.

In the left plot of Fig. 2 we chose $\alpha = \frac{1}{3}$, which leads to $\alpha + \alpha _D = \frac{1}{2}-\varepsilon$ and, indeed, we observe convergence in the local $L^2$-norm of almost order 1, which coincides with the theoretical rate obtained in Corollary 2.4. The error in the local $H^{-1/2}$-norm is smaller than the error in the $L^2$-norm, but does converge with the same rate, i.e., an improvement of Theorem 2.3 in the energy norm is not possible. The right plot in Fig. 2 shows the same quantities for the choice $\alpha =\frac{1}{8}$. Obviously, in this case the rates of convergence are lower, and the local $L^2$-error does not converge with the best possible rate of one, but rather with the expected rate of $N^{-19/24}=N^{-1/2-\alpha -\alpha _D}$, as predicted by Corollary 2.4.

**Fig. 3:**
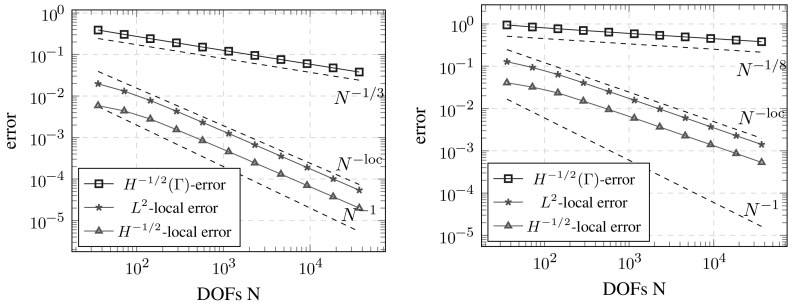
Local and global convergence of Galerkin-BEM for Symm’s equation, Z-shaped domain, left: $\alpha = \frac{1}{3}$, $\mathrm{loc} = \frac{19}{21}$, right: $\alpha = \frac{1}{8}$, $\mathrm{loc} = \frac{39}{56}$

### Example 2: Z-shaped domain

We consider the Z-shaped geometry depicted in Fig. 1 (right). Here, the dual problem permits solutions of regularity $H^{\alpha _D}(\varGamma )$ with $\alpha _D = \frac{1}{14}-\varepsilon$. Again, we observe the expected convergence $O(N^{-\alpha })$ for the global error in the energy norm in Fig. 3. However, in contrast to the previous example on the L-shaped domain, we do not obtain a rate of 1 for the local error in the $L^2$-norm for the case $\alpha =\frac{1}{3}$, but rather a rate of ${-19/21}$, since $\frac{1}{2}+\alpha _D + \alpha = \frac{19}{21}-\varepsilon$. For the choice $\alpha =\frac{1}{8}$, we observe convergence $\mathcal {O}(N^{-1/2-1/14-1/8}) = \mathcal {O}(N^{-39/56})$, which once more matches the theoretical convergence $N^{-1/2-\alpha -\alpha _D}$.
